# Hydrodeoxygenation of isocyanates: snapshots of a magnesium-mediated C=O bond cleavage†
†Electronic supplementary information (ESI) available: Experimental procedures and full characterisation data of compounds **2–7**, along with the catalytic protocols and NMR spectra. CCDC 1504016–1504019 contain the supplementary crystallographic data for compounds **2**, **3**, **4** and **6**, respectively, while the data for compound **5** and **7** have respective codes of CCDC 1526366–1526367. Computed Cartesian coordinates of all of the molecules reported in this study. For ESI and crystallographic data in CIF or other electronic format see DOI: 10.1039/c7sc00117g


**DOI:** 10.1039/c7sc00117g

**Published:** 2017-03-01

**Authors:** Yan Yang, Mathew D. Anker, Jian Fang, Mary F. Mahon, Laurent Maron, Catherine Weetman, Michael S. Hill

**Affiliations:** a Key Laboratory of Nonferrous Metal Chemistry and Resources Utilization of Gansu Province , School of Chemistry and Chemical Engineering , Lanzhou University , Lanzhou 730000 , PR China . Email: fangj@lzu.edu.cn; b Department of Chemistry , University of Bath , Bath BA2 7AY , UK . Email: msh27@bath.ac.uK; c LPCNO , Université de Toulouse , INSA Toulouse 135 , Avenue de Rangueil , 31077 Toulouse cedex , France

## Abstract

Organic isocyanates are readily converted to methyl amine products through their hydroboration with HBpin in the presence of a β-diketiminato magnesium catalyst. Although borylated amide and *N*-,*O*-bis(boryl)hemiaminal species have been identified as intermediates during the reductive catalysis, the overall reduction and C–O activation is metal-mediated and proposed to occur through the further intermediacy of well-defined magnesium formamidato, formamidatoborate and magnesium boryloxide derivatives. Examples of all these species have been identified and fully characterised through stoichiometric reactivity studies and the stability of the borate species leads us to suggest that, under catalytic conditions, the onward progress of the deoxygenation reaction is crucially dependent on the further activation provided by the Lewis acidic HBpin substrate. These deductions have been explored and ratified through a DFT study.

## Abstract 2

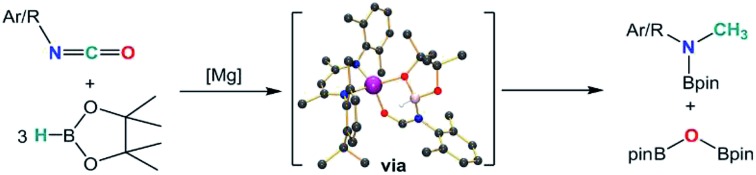
Organic isocyanates are readily converted to methyl amine products through their hydroboration with HBpin in the presence of a β-diketiminato magnesium catalyst.

## Introduction

**Scheme 1:**
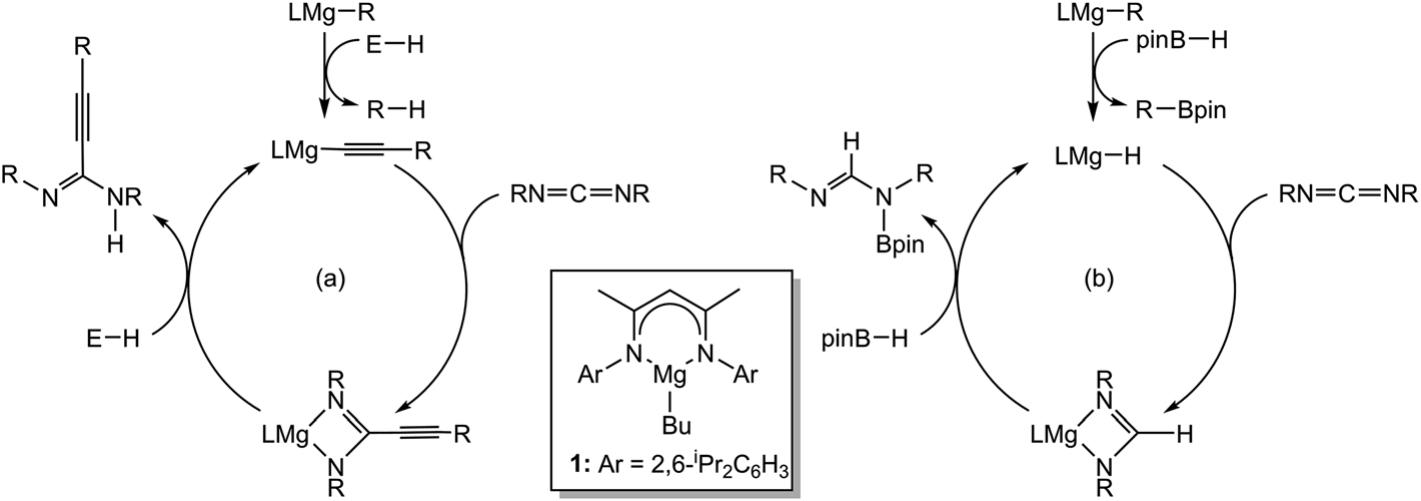
Magnesium-catalysed reactivity of carbodiimides.

The utility of reagents derived from magnesium in homogeneous catalysis has advanced rapidly during the last decade.^1^ Our own investigations have centred on the readily available and easily synthesised β-diketiminato magnesium *n*-butyl derivative [HC{(Me)CN(Dipp)}_2_Mg-*n*-Bu] (Dipp = 2,6-di-isopropylphenyl) (**1**) as a convenient prototype reagent for the development of a number of molecular catalytic transformations. We have shown that use of compound **1** enables the heterofunctionalisation and hydroboration of an assortment of multiply bonded and heteroatomic C=E (E = O,^2^ NR^3^,^4^), and C≡E (E = O,^5^ N^6^) substrates,^7^ for example, in the hydroacetylenation and hydroboration of carbodiimides (Scheme 1).3c,d

Hydrodeoxygenation of carbonyl-containing compounds (*e.g.* aldehydes, ketones, amides) has attracted increasing attention given its potential applications in biofuels and fine chemical syntheses.^8^ Although a number of stoichiometric protocols for the deoxygenation of ketones are known,^9^–^11^ these methods typically require harsh reaction conditions. In a catalytic context, Stephan has very recently reported a mild protocol for the deoxygenation of ketones in the presence of silanes in conjunction with a highly electrophilic phosphonium cation.^12^ Similarly, the reduction of primary, secondary and tertiary amides to amines with silane hydride sources may be achieved under either transition metal-catalysed^13^ or ‘metal-free’ conditions through the use of catalytic amounts of electrophilic boranes.^14^ Of greater relevance to the current work are a number of Mg-based processes that have also been described. In an initial report, we observed that compound **1** was able to effect ester cleavage during the hydroboration of 3-methylnicotinate with pinacolborane (HBpin).^15^ This reactivity was subsequently extended by Sadow and co-workers to a variety of ester hydroboration and amide reduction reactions mediated by a magnesium tris(oxazolinyl)phenylborate catalyst.^16^ Importantly, mechanistic analysis of these latter reactions implicated the intermediacy of zwitterionic alkoxyborate intermediates rather than a conventional σ-bond metathesis pathway. In related work, we have shown that carbon dioxide may be subjected to the complete and selective rupture of a C=O bond and the formation of the methanol-equivalent H_3_COBpin through the use of a modified catalyst system derived from **1** and a B(C_6_F_5_)_3_ co-catalyst.^17^ With this reactivity in mind, we speculated that analogous hydrodeoxygenation could be applied to the isoelectronic isocyanate function (eqn (1)) to provide a more amenable system from which to develop a detailed understanding of the molecular basis for such Mg-centred C=O activation. Although a Schwartz reagent-mediated reduction of isocyanates has been described as a straightforward and direct route to otherwise inaccessible formamides and the *in situ* hydroboration of *t*-BuN=C=O with HBpin catalysed by [Mg(THF)_6_][HBPh_3_] has been very recently reported to provide the bis-borylated hemiaminal, *t*-BuN(Bpin)CH_2_OBpin,^18^,^19^ the successful development of the reactivity shown in eqn (1) would also enable a viable and, to the best of our knowledge, unprecedented catalytic route to methyl amines.
1

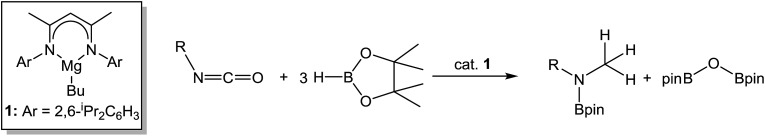




## Results and discussion

### Catalytic reactivity

An initial reaction between i-PrNCO and three molar equivalents of HBpin performed at 60 °C in C_6_D_6_ evidenced no observable reaction after 12 hours by ^1^H and ^11^B NMR spectroscopy. In contrast, similar monitoring of an otherwise identical reaction performed at 60 °C in the presence of 10 mol% of compound **1** for 1.5 hours indicated the consumption of *ca.* 76% of the isocyanate and borane starting materials. More notably, the formation of a predominant new organic product was apparent from the appearance of a singlet resonance at 2.56 ppm. Analysis of the corresponding ^11^B NMR spectrum revealed the presence of unreacted HBpin along with two further singlet resonances in a 1 : 2 ratio by integration at *δ* 27.3 and 24.9 ppm respectively and a further broad but low intensity signal at *δ* 7.8 ppm, which was observed to persist throughout the course of the reaction. The resonance centred at *δ* 24.9 ppm was identified as the bis(boryl)oxide, O(Bpin)_2_, through comparison with literature data^20^ indicating that HBpin reduction of i-PrNCO had resulted in cleavage of the C=O bond within the substrate with consequent formation of the *N*-borylated *N*-methyl isopropylamine (Table 1, entry 1).

**Table 1:** Magnesium-catalysed hydroboration and reductive C=O cleavage of organic isocyanates

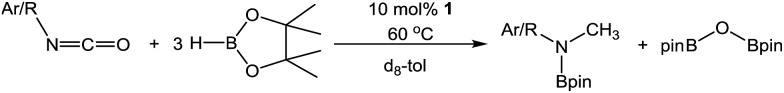					
Entry	Ar/R	Catalyst (mol%)	Time (h)	Temp (°C)	NMR conv.^*a*^ (%)
1	i-Pr	10	1.5	60	76
2	Ad	10	4.5	60	75
3	Et	10	21	60	70
4	*n*-Pr	10	21	60	75
5	*t*-Bu	10	3.5	60	81
6	Cy	10	4.5	60	90
7	Ph	10	24	25	86
8	Mes	10	24	60	90
9	Dipp	10	24	60	50

^*a*^To *N*-methyl amine product.

Extension of this reactivity to other commercially available alkyl isocyanates provided similar reactivity to i-PrNCO. Entries 1 to 6 in Table 1 present the outcome of these reactions performed with a range of alkyl isocyanates of varying steric bulk. All the reactions were characterised by the appearance of a singlet methyl ^1^H NMR resonance in the range *δ* 2.5–3.0 ppm and a single new *N*-B resonance at *ca. δ* 27 ppm, which appeared along with the signal associated with the O(Bpin)_2_ by-product in the ^11^B NMR spectra. Contrary to expectation, increased RNCO substituent steric demands provided generally faster reaction times; R = i-Pr (entry 1) and *t*-Bu (entry 5) evidenced complete consumption of the isocyanate in less than 4 hours compared to Et (entry 3) and *n*-Pr (entry 4) both of which required 21 hours. The increased reactivity associated with enhanced alkyl substituent steric demands also coincided with the onset of secondary side reactions. Although attempts to identify the products of these side reactions were unsuccessful it is likely that oligomerisation of the isocyanates is competitive with the reductive C=O cleavage.^21^ Notably, further reactions performed with *N*-phenyl and *N*-mesityl (Mes) isocyanate substitution provided *ca.* 90% conversion in 24 hours (entries 7 and 8), while an increase of the *N*-aryl substituent's steric demands to 2,6-di-isopropylphenyl (Dipp) resulted in a significantly reduced conversion during the same time period (entry 9).

### Mechanistic investigations

**Fig. 1:**
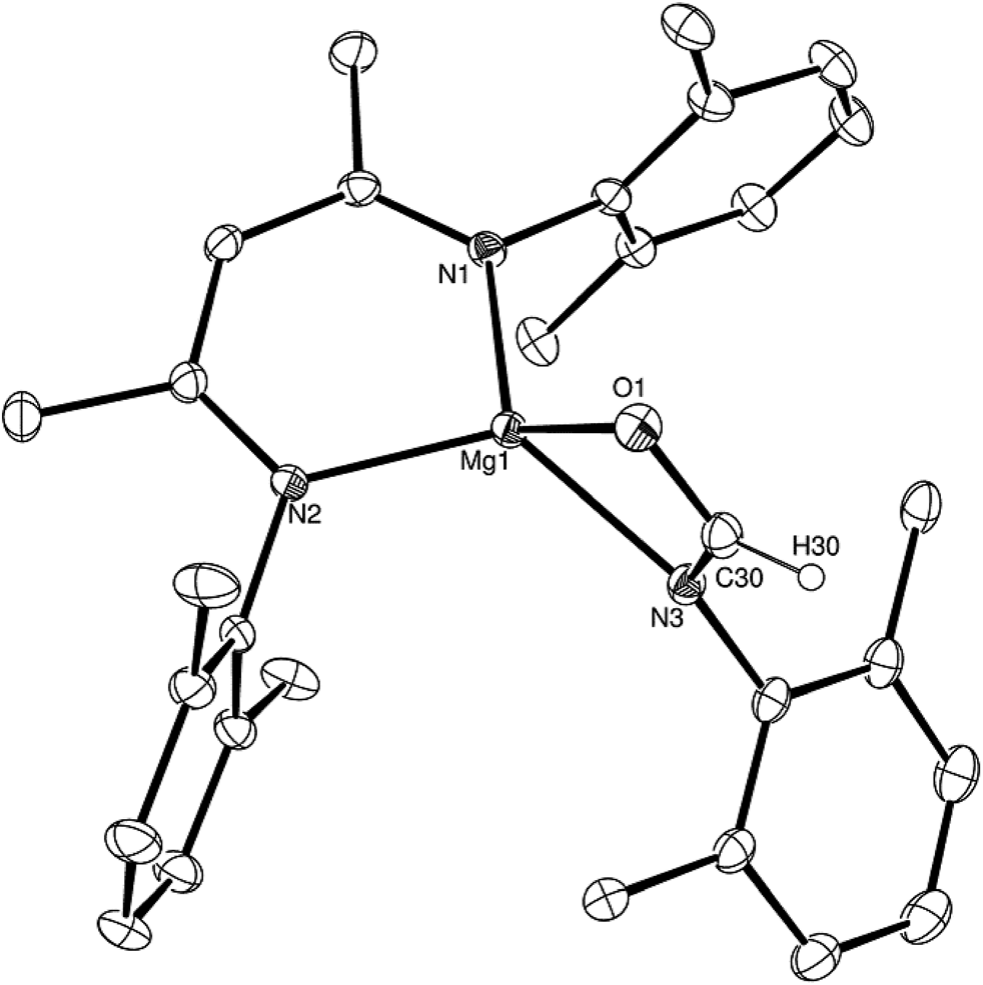
ORTEP representation (25% probability ellipsoids) of compound **2**. Hydrogen atoms except for H(30) and iso-propyl methyl groups are removed for clarity. Selected bond lengths (Å) and angles (°): Mg(1)–O(1) 2.0118(10), Mg(1)–N(1) 2.0179(11), Mg(1)–N(2) 2.0156(11), Mg(1)–N(3) 2.0983(11), Mg(1)–C(30) 2.3719(14), O(1)–C(30) 1.2806(17); O(1)–Mg(1)–N(1) 121.64(5), O(1)–Mg(1)–N(2) 122.69(4), O(1)–Mg(1)–N(3) 65.86(4), O(1)–Mg(1)–C(30) 32.68(4), N(1)–Mg(1)–N(3) 128.04(4), N(1)–Mg(1)–C(30) 132.71(5), N(2)–Mg(1)–N(1) 95.09(4), N(2)–Mg(1)–N(3) 125.20(5), N(2)–Mg(1)–C(30) 131.76(5), N(3)–Mg(1)–C(30) 33.18(5).

**Fig. 2:**
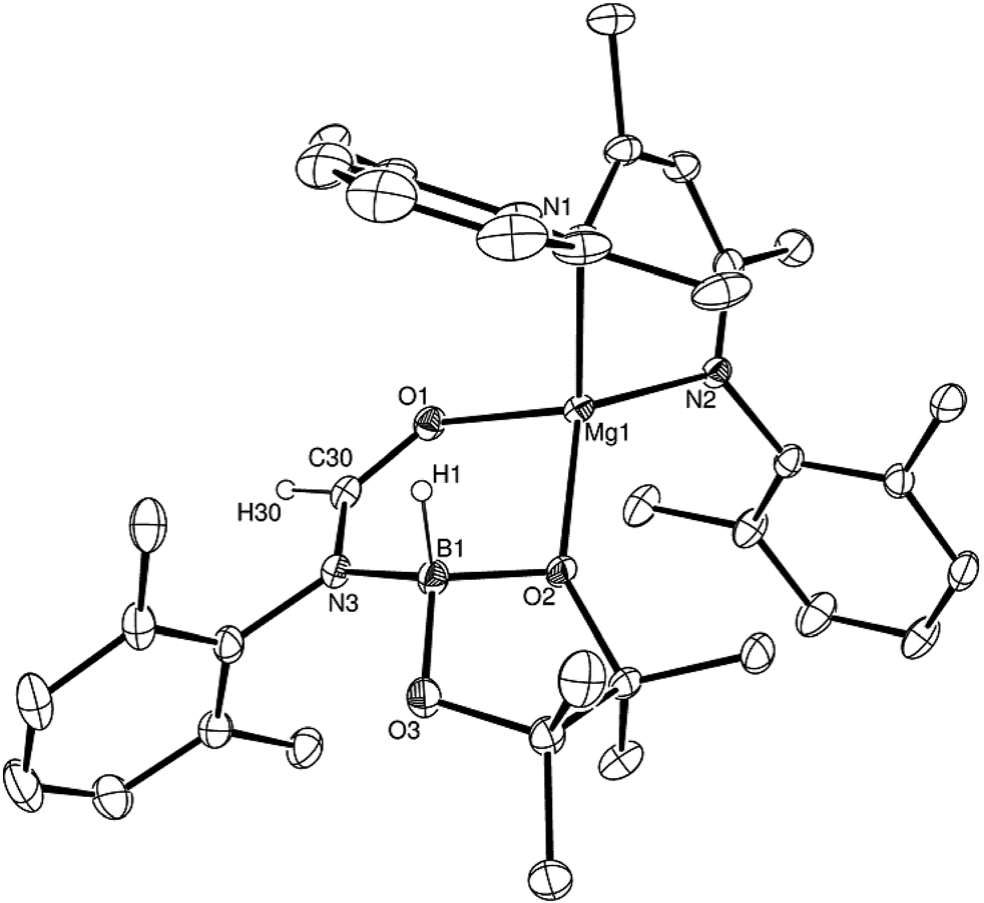
ORTEP representation (25% probability ellipsoids) of compound **3**. Hydrogen atoms except for H(1), H(30) and iso-propyl methyl groups are removed for clarity. Selected bond lengths (Å) and angles (°): Mg(1)–O(1) 1.9631(12), Mg(1)–O(2) 1.9734(12), Mg(1)–N(1) 2.0547(14), Mg(1)–N(2) 2.0320(14), N(3)–B(1) 1.590(2), O(2)–B(1) 1.516(2), O(3)–B(1) 1.427(2); O(1)–Mg(1)–O(2) 91.39(5), O(1)–Mg(1)–N(1) 106.55(6), O(1)–Mg(1)–N(2) 115.27(5), O(2)–Mg(1)–N(1) 131.39(6), O(2)–Mg(1)–N(2) 120.02(5).

Having established the viability of this magnesium-centred hydrodeoxygenation, a series of stoichiometric reactions were undertaken to inform our understanding of the elementary processes operant during catalysis. We have previously described that addition of HBpin to a solution of compound **1** at room temperature leads to stoichiometric formation of *n*-BuBpin and a may act as a source of a heteroleptic magnesium hydride species.^2^ Addition of a single equivalent of the sterically encumbered DippNCO to a solution prepared in this manner provided stoichiometric conversion to the magnesium formamidate species, compound **2**, the formation of which was clearly apparent from the appearance of a new downfield (1H by relative integration) singlet resonance at *δ* 7.92 ppm in the resultant ^1^H NMR spectrum. Crystallisation of compound **2** direct from the reaction solution provided crystals suitable for a single crystal X-ray diffraction analysis. The results of this analysis (Fig. 1) confirmed that compound **2** was a magnesium formamidate, the mononuclear constitution of which is most likely imposed by the steric demands of the three *N*-Dipp substituents (Fig. 2).

Addition of one equivalent of HBpin to a solution of compound **2** in *d*_8_-toluene resulted in the formation of a single species, compound **3**, which was characterised by the appearance of a new resonance at *δ* 4.3 ppm in the ^11^B NMR spectrum. A subsequent single crystal X-ray diffraction analysis confirmed compound **3** to be a borate species formed by the formal insertion of HBpin into the Mg–N bond of the magnesium amidate. The boron-bound hydride (H1) was readily located and freely refined, clearly demonstrating that there was no significant Mg–H interaction in the solid state. The structure of compound **3** is reminiscent of a previously described borate species resulting from addition of HBpin to a magnesium formamidinate supported by the same β-diketiminate ligand.3*c*

**Fig. 3:**
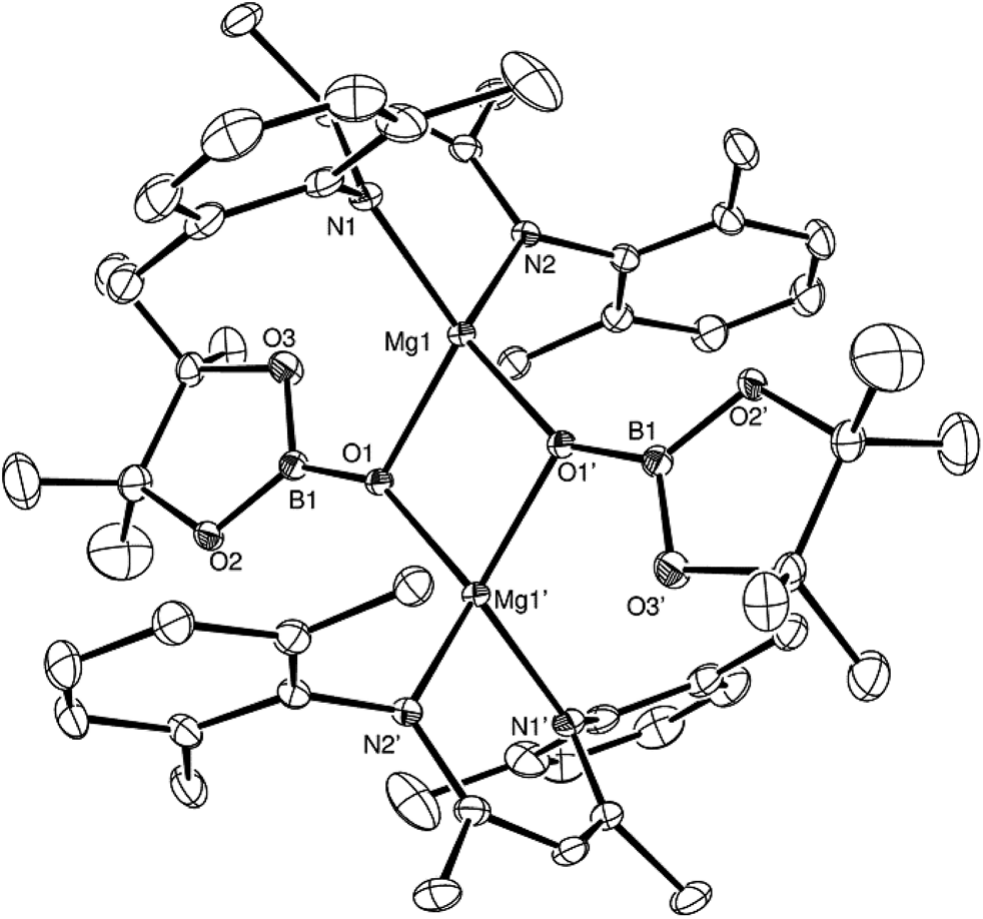
ORTEP representation (25% probability ellipsoids) of compound **4**. Hydrogen atoms and iso-propyl methyl groups are removed for clarity. Selected bond lengths (Å) and angles (°): Mg(1)–O(1) 2.0312(12), Mg(1)–N1 2.0811(14), Mg(1)–N(2) 2.0770(14), Mg(1)–O(1′) 1.9798(11), O(1)′–Mg(1)–O(1) 81.84(5), O(1)–Mg(1)–N(1) 118.84(6), O(1)′–Mg(1)–N(1) 123.32(6), O(1)–Mg(1)–N(2) 119.82(5), O(1)′–Mg(1)–N(2) 122.50(5), O(1)–Mg(1)–B(1) 25.45(5), O(1)′–Mg(1)–B(1) 107.30(5), N(1)–Mg(1)–Mg(1)′ 133.00(5), N(1)–Mg(1)–B(1) 103.32(6), N(2)–Mg(1)–Mg(1)′ 133.18(5), N(2)–Mg(1)–N(1) 93.55(6), N(2)–Mg(1)–B(1) 104.05(5). Symmetry transformations used to generate equivalent atoms –*x*, –*y*, –*z*.

Attempts to induce the extrusion of the *N*-borylated formamidine, DippN(Bpin)HC(O) (compound **7**, *vide infra*), by prolonged (>24 hours) heating of samples of compound **3** in *d*_8_-toluene either at 60 °C or 80 °C failed to provide any evidence of onward reactivity. Similar treatment of **3** at the higher temperature of 100 °C for 16 hours, however, resulted in the production of a single new β-diketiminato species (**4**) along with the methylene imine, DippN=CH_2_, which was tentatively identified through the appearance of an AB spin system in the ^1^H NMR spectrum with signals at *δ* 6.90 and 7.25 ppm. The identity of compound **4** was confirmed through the isolation of single crystals suitable for an X-ray diffraction experiment. The results of this analysis revealed that **4** was a dimeric magnesium boryloxide species (Fig. 3) arising from the cleavage of the C–O bond of the amidato moiety of compound **3**.

While the formation of compound **4** implicates the direct extrusion of DippN=CH_2_ as the mode of C–O activation, the elevated temperature required for this reaction militates against its operation during the catalytic reactions summarised in Table 1. A further reaction between compound **3** and a single equivalent of HBpin was, thus, undertaken. Although no consumption of **3** took place at room temperature, heating of this *d*_8_-toluene solution at 60 °C for 8 hours resulted in the predominant formation of DippN(Me)Bpin along with a variety of β-diketiminato magnesium species which could not be identified with any meaningful level of confidence. In an attempt to shed further light on this reactivity, compound **1** was reacted with 3 molar equivalents of HBpin and 2 equivalents of DippNCO. This reaction at room temperature surprisingly resulted in the generation of the amidate derivative (**2**) along with the concomitant production of a single new compound (**5**). This latter species was identified as the bis-borylated hemiaminal, Dipp(pinB)NCH_2_OBpin, through the appearance of a distinctive methylene singlet resonance at *δ* 5.35 ppm in the ^1^H NMR spectrum and two broad signals of equal intensity at *δ* 28.3 and 24.8 ppm in the corresponding ^11^B NMR experiment. Furthermore, continued treatment of this reaction mixture with additional equivalents of HBpin and DippNCO in a 2 : 1 ratio provided for the catalytic production of compound **5**.

**Fig. 4:**
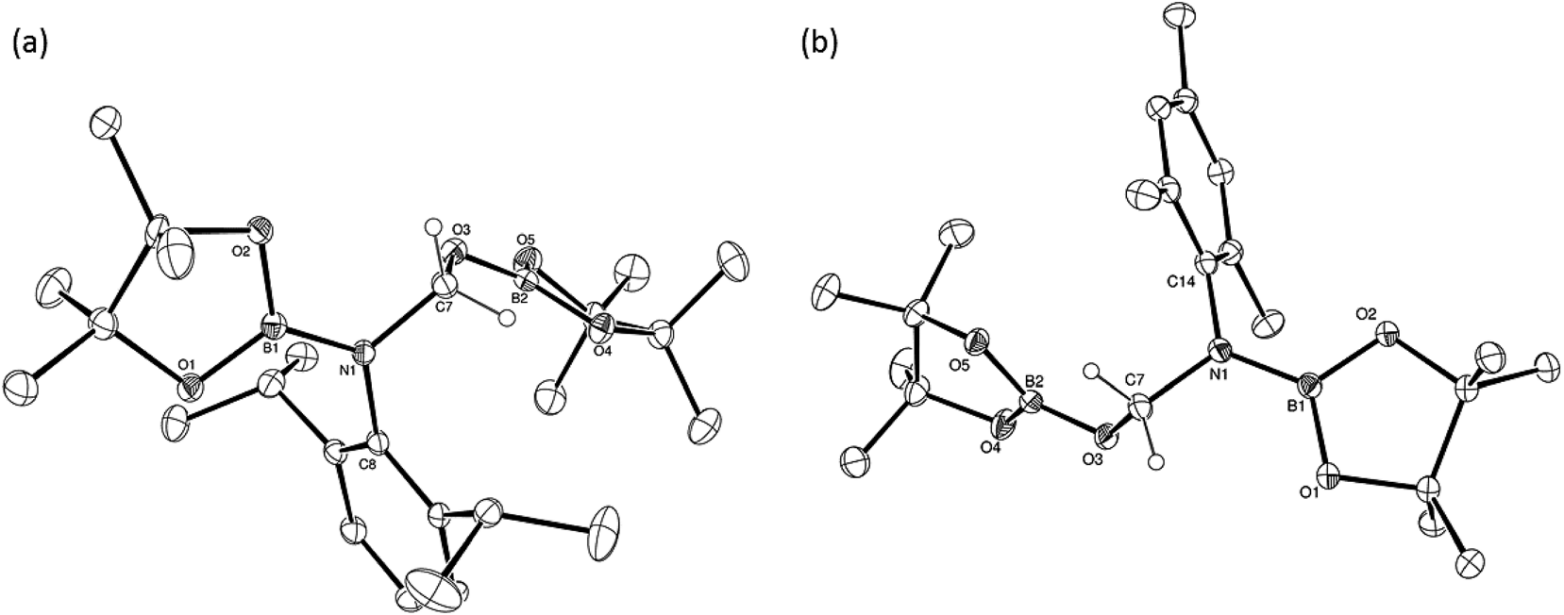
ORTEP representations (25% probability ellipsoids) of (a) compound **5** and (b) compound **6**. The B(1)-containing molecule of **6** and hydrogen atoms except those attached to C(7) are removed for clarity in both **5** and **6**. Selected bond lengths (Å) and angles (°): **5**: N(1)–B(1) 1.411(4), N(1)–C(7) 1.440(3), O(3)–C(7) 1.427(3), O(3)–B(2) 1.353(4), B(1)–N(1)–C(7) 119.5(2), B(1)–N(1)–C(8) 124.5(2), B(2)–O(3)–C(7) 119.0(2). **6**: N(1)–B(1) 1.414(2), N(1)–C(7) 1.4392(18), O(3)–C(7) 1.4404(18), O(3)–B(2) 1.3571(19), B(1)–N(1)–C(7) 120.81(12), B(1)–N(1)–C(14) 121.42(12), B(2)–O(3)–C(7) 119.12(12).

Subsequent investigation revealed that this catalytic reactivity could be straightforwardly replicated through the reactions of either DippNCO or MesNCO with two molar equivalents of HBpin performed in the presence of 10 mol% **1** to provide compound **5** and the analogous *N*-mesityl species (**6**) within 10 minutes at room temperature (Table 2, entries 1 and 2). Repetition of the latter reaction with half the catalyst loading (5 mol%) also provided efficient turnover at room temperature with similar full conversion being observed after 15 minutes (Table 2, entry 3). Subsequent preparative scale reactions provided single crystals of compounds **5** and **6** suitable for X-ray diffraction analysis which confirmed the formation of the bis-borylated hemiaminal species (Fig. 4).

**Table 2:** Magnesium-catalysed di-hydroboration of aryl isocyanates

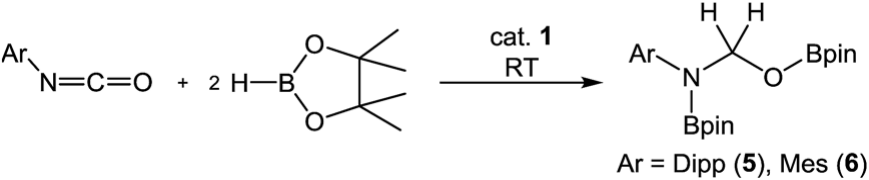					
Entry	Ar	Catalyst (mol%)	Time (min)	Temp. (°C)	NMR conv. (%)
1	Dipp	10	15	25	>99
2	Mes	10	<5	25	>99
3	Mes	5	10	25	>99

Attempted extension of this bis-borylation protocol to PhNCO and the range of *N*-aliphatic isocyanates shown in Table 1 was unsuccessful with no evidence of any reactivity observed at room temperature beyond the initial activation of the pre-catalyst. Although subsequent heating of these reactions at 60 °C induced consumption of the borane reagent, this procedure also resulted in C–O cleavage and *ca.* 50% conversion of the isocyanate to the *N*-methylated amine product and the bis(boryl)oxide, O(Bpin)_2_, by-product.

To provide further insight into the role of such hemiaminal species as potential intermediates during the course of the C–O activation catalysis, a reaction was carried out between compound **6** and Jones' β-diketiminato magnesium hydride complex, [CH{C(Me)NDipp}_2_MgH]_2_.^22^ Monitoring of this reaction by ^1^H NMR spectroscopy demonstrated that heating to 60 °C resulted in the generation of the *N*-borylated methyl amine, pinBN(Me)Mes, along with the magnesium boryloxide (**4**) (eqn (2)). A further reaction performed by addition of compound **6** to a *d*_8_-toluene solution of the pre-catalyst (**1**) and HBpin provided a similar result. Subsequent addition of HBpin to either of these reaction mixtures or to a solution of an isolated sample of compound **4** in *d*_8_-THF resulted in the consumption of the magnesium boryloxide with concomitant production of (pinB)_2_O and the regeneration of [CH{C(Me)NDipp}_2_MgH]_2_ (eqn (3)). Although these routes to compound **4** and its reactivity with HBpin suggest that the C–O cleavage reaction is primarily magnesium mediated, it is notable that heating of a solution of compound **6** at 60 °C for 12 hours in the absence of any other reagents also resulted in the consumption of *ca.* 50% of the hemiaminal and production of (pinB)_2_O and the methylene imine, MesN=CH_2_.
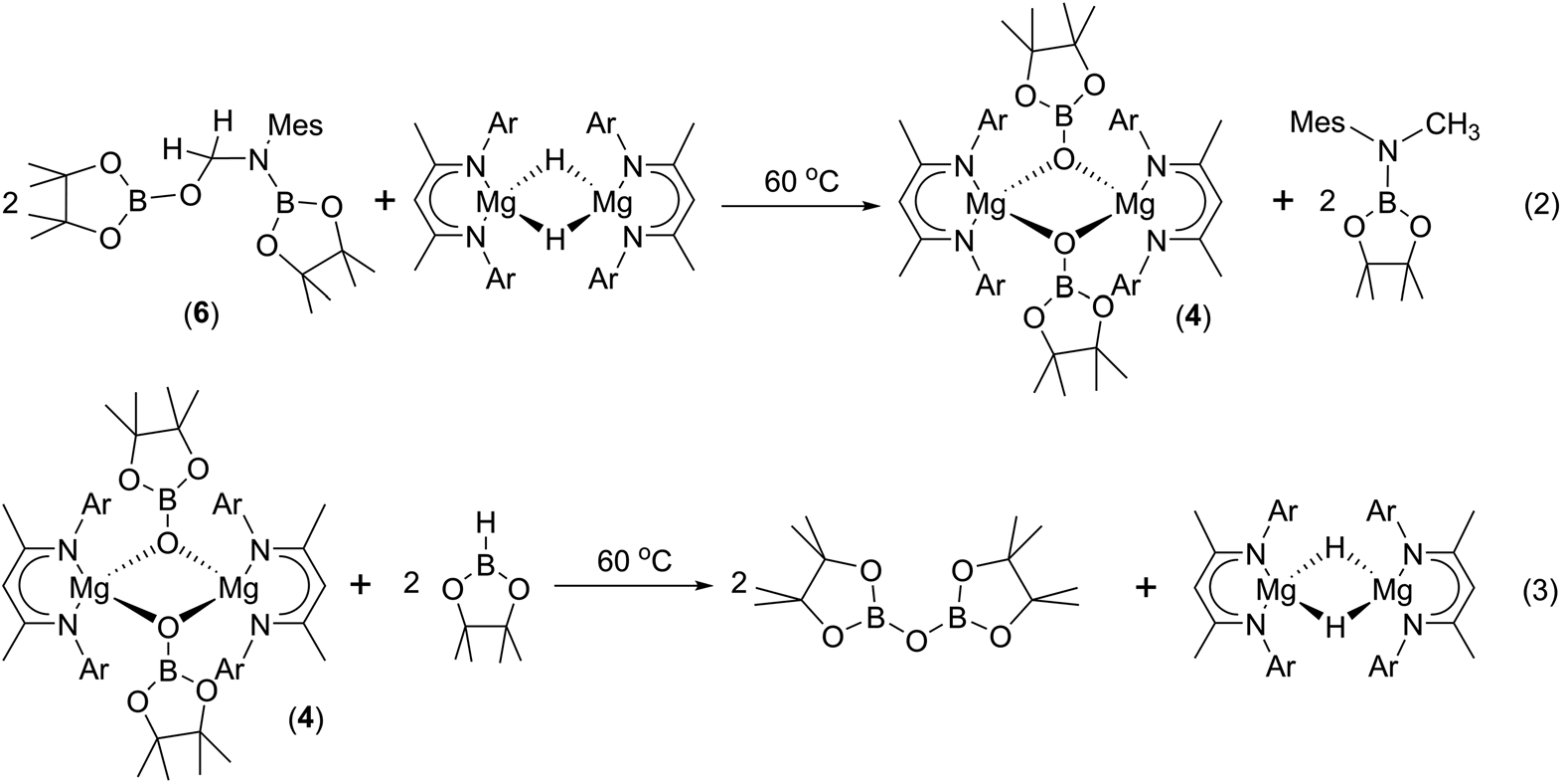


**Fig. 5:**
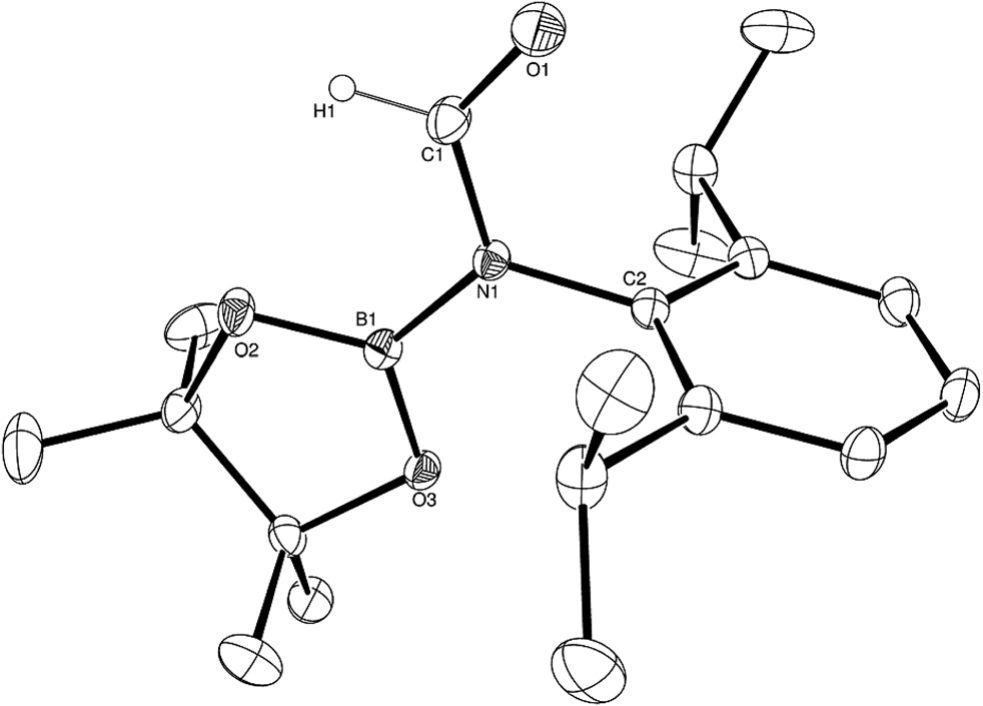
ORTEP representation of compound **7** with thermal ellipsoids set at 25% level of probability. Hydrogen atoms have been removed for clarity except the hydrogen attached to C1. Selected bond lengths (Å) and bond angles (°): O1–C1 1.206(4), N1–C1 1.376(3), N1–B1 1.443(3), N1–C2 1.444(3). O1–C1–N1 124.9(2), C1–N1–B1 120.7(2), C1–N1–C2 117.4(2).

An additional intermediate was identified during our investigation of the catalytic production of compound **5**. Although a ^1^H NMR spectrum recorded five minutes after the initiation of the catalytic reduction of DippNCO with two equivalents of HBpin catalysed by **1** demonstrated the facile production of compound **5**, the formation of this species was accompanied by a new compound (**7**) characterised by the appearance of a high frequency singlet resonance at *δ* 9.04 ppm in the ^1^H NMR spectrum. Compound **7** was assigned as the *N*-borylated formamidine, DippN(Bpin)HC(O), which was completely consumed upon completion of the reaction. Several attempts to generate compound **7** by the reaction of a single equivalent of HBpin and DippNCO in the presence of 10 mol% **1** resulted in the consumption of only 50% of the isocyanate starting material and the production of compound **5**. A similar reaction performed at lower catalyst loading (0.1 mol%) and in the minimum amount of toluene solvent, however, resulted in the formation of a significant quantity of crystalline material. Mechanical separation of single crystals formed within this material enabled a series of single crystal X-ray diffraction analyses which showed it to be primarily a mixture of compound **5** and compound **7**. The results of the analysis of compound **7** are shown in Fig. 5, which demonstrates the O(1)–C(1) bond distance [1.206(4) Å] and O(1)–C(1)–N(1) angle [24.9(2)°] to be consistent with the formation of the formamide function.

**Fig. 6:**
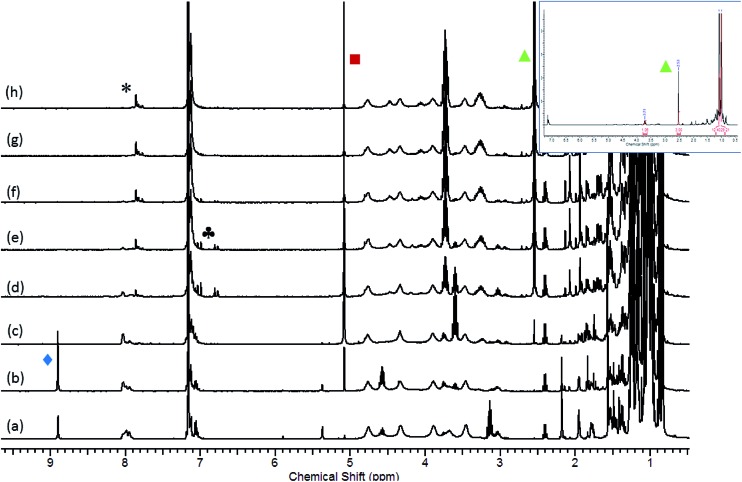
Stacked ^1^H NMR spectra (400 MHz) for the reaction of HBpin and i-PrNCO catalysed by 10 mol% **1** at 60 °C. Spectra recorded at 15 minute intervals between (a) *t* = 0 min and (h) *t* = 105 min. Inset spectrum illustrates the ^1^H NMR spectrum of the same reaction after 180 minutes. (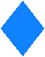 = i-PrN(Bpin)HC(O), 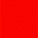 = i-PrN(Bpin)CH_2_OBpin; 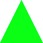 = i-PrN(Bpin)CH_3_; 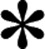 borate intermediate similar to compound **3**.

The significance of these observations with respect to the catalytic reactivity was assessed by *in situ* monitoring (^1^H NMR spectroscopy) of a reaction of HBpin and i-PrNCO catalysed by 10 mol% **1** at 60 °C. Fig. 6 illustrates the evolution of this reaction over 105 minutes and highlights a complex profile *en route* to the ultimate methyl amine product (inset spectrum, Fig. 6). In the earliest stages of the reaction a new compound was characterised by the appearance of a high frequency singlet resonance at *δ* 8.90 ppm (indicated by the 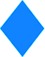 identifier in Fig. 6). This species, which we assign as an *N*-borylated formamidine, i-PrN(Bpin)HC(O), analogous to compound **7**, was observed to accumulate during the initial 15 minutes of the reaction prior to any observable methyl amine formation (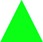). After this time, however, it was consumed simultaneously with the appearance of significant quantities of a bis-borylated hemiaminal derivative, i-Pr(pinB)NCH_2_OBpin, analogous to compounds **5** and **6**, which was apparent as a persistent singlet resonance at *δ* 5.08 ppm (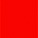). A further low field singlet was observed at *δ* 7.80 ppm (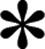). Once evolved, this latter resonance persisted at an effectively constant intensity throughout the course of the catalysis. This chemical shift is reminiscent of the signal associated with the amidato methine proton observed for compound **3** (*δ* 7.69 ppm) and leads us to postulate that a borate species with a comparable constitution is also formed in a steady state concentration as a key intermediate during the reduction of i-PrNCO. The initial appearance of this species was accompanied by the emergence of a clear AB spin system (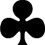), which may be cautiously assigned to the methylene imine, i-PrN=CH_2_. This small molecule is evidently consumed, however, at more mature phases of the catalysis.

**Fig. 7:**
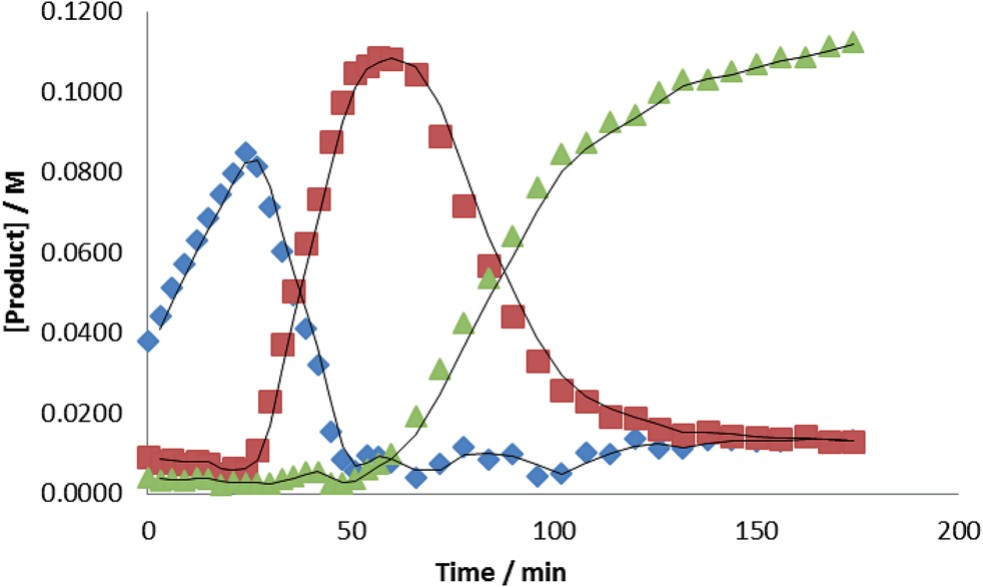
Plot of [product] *versus* time for the hydroboration of i-PrNCO catalysed by 10 mol% **1** at 60 °C. (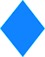 = i-PrN(Bpin)HC(O), 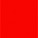 = i-PrN(Bpin)CH_2_OBpin; 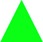 = i-PrN(Bpin)CH_3_).

Evidence for a sequential mechanism was provided by examination of the temporal evolution of the signals assigned to i-PrN(Bpin)HC(O) (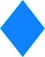), i-Pr(pinB)NCH_2_OBpin (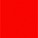) and i-PrN(Bpin)Me (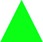) during the reduction of i-PrNCO. Fig. 7 illustrates that the observed intermediate products accumulate during the course of the reactions in quantities greater than that of the catalyst loading, suggesting that the formamidine and hemiaminal species are released but can re-insert into the catalytic cycle to undergo further reduction. Notably, the accumulation of the ultimate methyl amine product describes a sigmoidal curve reminiscent that observed during *N*-borylated amidine production in our recent report of carbodiimide hydroboration catalysed by **1**.3*c* Similar sigmoidal profiles are also typical of enzyme kinetics and generally associated with the positive cooperative binding of substrate molecules, which is implied by the thermal stability of compound **3** in the absence of further HBpin.

Although these observations attest to a complex mechanistic landscape, we postulate that the gross features of this reactivity may be rationalised by the generic mechanism for the reduction of RNCO illustrated in Scheme 3. No attempt is made to delineate potentially dimeric or monomeric intermediates. In common with our previous mechanistic studies of a variety of reactions catalysed by β-diketiminato magnesium centres, however, we suggest that a necessity for dimer to monomer pre-equilibration is likely to exert a crucial influence on the kinetics of the individual reactions.^6^ Although we also suggest that the persistence of any magnesium hydride is doubtful under catalytic conditions, catalysis is initiated through borane activation of the pre-catalyst (**1**, not shown) to provide the transient magnesium hydride (or borohydride) necessary to generate formamidate and borate intermediates analogous to compounds **2** and **3**. The apparent stability of this latter compound mitigated against its direct thermal conversion to boron-containing small molecules at temperatures relevant to the catalysis. We, thus, suggest that the most kinetically accessible cycle necessitates breakdown of a similar borate species to produce RN(Bpin)HC(O).

We postulate that this process occurs by the necessary activation of the borate intermediate through its interaction with a further equivalent of Lewis acidic HBpin prior to intramolecular boron to magnesium hydride transfer. This process is, thus, reminiscent of our recent reports on related magnesium borate species identified during the hydroboration of organic carbodiimides and nitriles.3c,6b Although viable concentrations of the *N*-borylated formamidine were observed to accumulate during the early stages of the catalytic reduction of i-PrNCO, this species is apparently consumed through its onward reaction with magnesium hydride. We have not identified any resultant magnesium hemiaminalate species but suggest that compounds of this type will be rapidly consumed through further B–H/Mg–O metathesis to yield R(pinB)NCH_2_OBpin. The ultimate production of RN(Bpin)Me and closure of the catalytic cycle are then predicated upon a sequence of C–O/Mg–H and Mg–O/B–H metathesis steps reminiscent of the stoichiometric reactivity summarised for the isolable species **6** and **4** in eqn (2) and (3).

Although our observations suggest that this reaction sequence predominates under the mild conditions applied during catalysis, the mechanism must also reconcile the production and consumption of RN=CH_2_. It is notable that appreciable quantities of i-PrN=CH_2_ were only observed after the generation of significant amounts of i-Pr(pinB)NCH_2_OBpin during the reduction of i-PrNCO. We infer, therefore, that this small molecule is generated by the direct decomposition of i-Pr(pinB)NCH_2_OBpin rather than any higher energy magnesium-centered process and implicates a second plausible pathway for the production of i-PrN(Bpin)CH_3_.

### Computational investigation

**Scheme 2:**
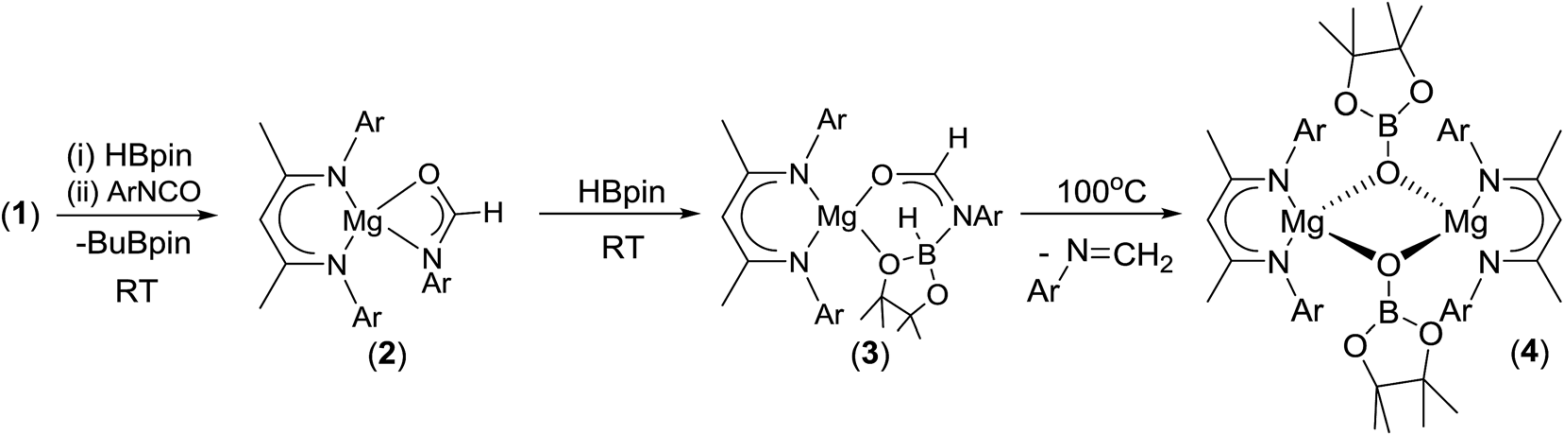
Synthesis of compounds **2–4** (Ar = 2,6-di-isopropylphenyl).

**Fig. 8:**
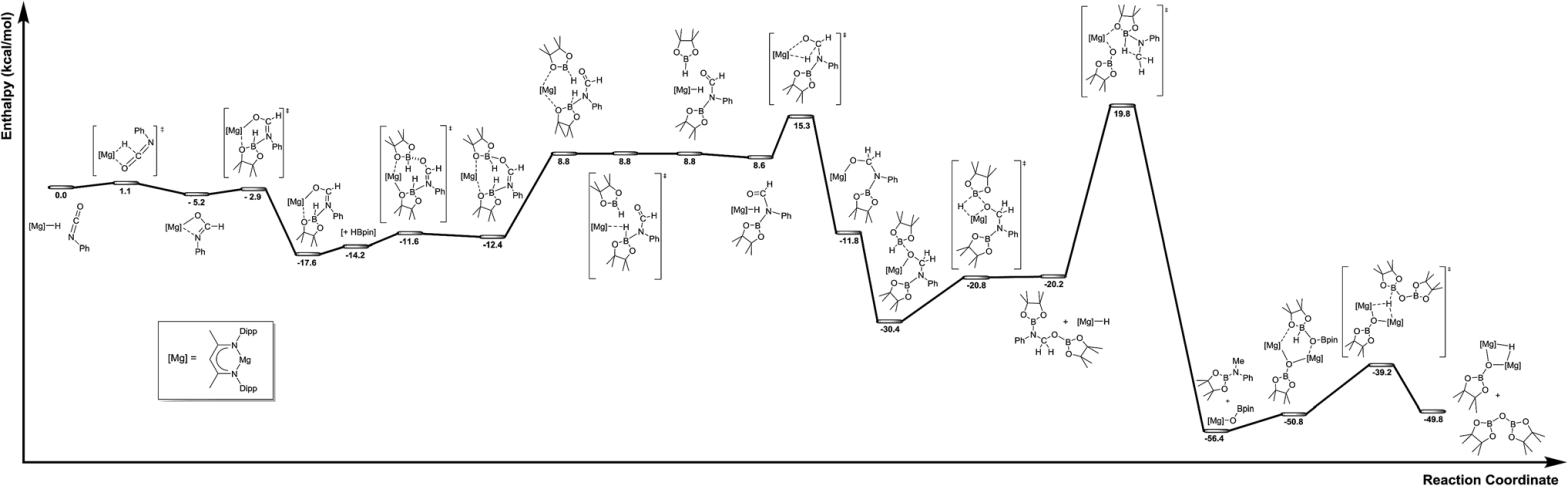
Calculated reaction pathway for the magnesium-catalysed hydrodeoxygenation of organic isocyanates.

The mechanism proposed in Scheme 2 was investigated by density functional theory (DFT) for the magnesium-catalysed hydroboration of PhNCO. Calculations (B3PW91, see ESI†) validated all the individual reaction steps for the complete structures shown in Fig. 8. The effect of dispersion corrections (see ESI†) did not change the gross features of the reaction sequence but resulted in a significant decrease in the various activation barriers, especially when bimetallic species are involved. The following discussion refers solely to the dispersion corrected values. Species akin to compounds **2** and **3** were found to be formed by an overall exothermic (Δ*H* = –17.6 kcal mol^–1^) sequence of isocyanate Mg–H insertion and subsequent reaction of the as-formed magnesium amidate with HBpin. As deduced from the stability of compound **3**, the production of a borylated formamide analogous to compound **7** entails the interaction of the magnesium amidatoborate with a second equivalent of HBpin to enable the necessary boron to magnesium hydride transfer. While the production of compounds such as **7** requires the traversal of a several higher energy transition states and intermediates, mainly associated with the HBpin insertion into the Mg–O bond of **3** at a cost of 5.0 kcal mol^–1^ and the need for B–O bond disruption (21.6 kcal mol^–1^), these processes occur prior to a kinetically facile (barrier of 6.7 kcal mol^–1^) and highly exothermic re-insertion of the carbonyl function (Δ*H* = –39.0 kcal mol^–1^) into the magnesium hydride bond. The subsequent generation of bis(boryl)hemiaminals such as **5** and **6** occurs *via* B–H/Mg–O sigma bond metathesis, which incurs an enthalpic penalty of some 10.2 kcal mol^–1^ associated to the formation of a monomeric Mg–H complex rather than its very stable dimeric form. However, while this is lower than the entrance channel (–20.2 kcal mol^–1^), **5** can only be considered as an intermediate in the course of the formation of methyl amine product in line with the proposed catalytic cycle. The ultimate cleavage of the C–O bond and production of the methyl amine product requires traversal of an energy barrier of some 40.0 kcal mol^–1^ involving the further interaction of these reduced intermediates with a magnesium hydride, whereupon the formation of a boryloxide derivative similar to compound **4** is significantly exothermic (Δ*H* = –36.2 kcal mol^–1^). Dimerisation and a further Mg–O/H–B metathesis with a further molecule of HBpin yield (pinB)_2_O with the regeneration of the magnesium hydride (not shown in Fig. 8).

**Scheme 3:**
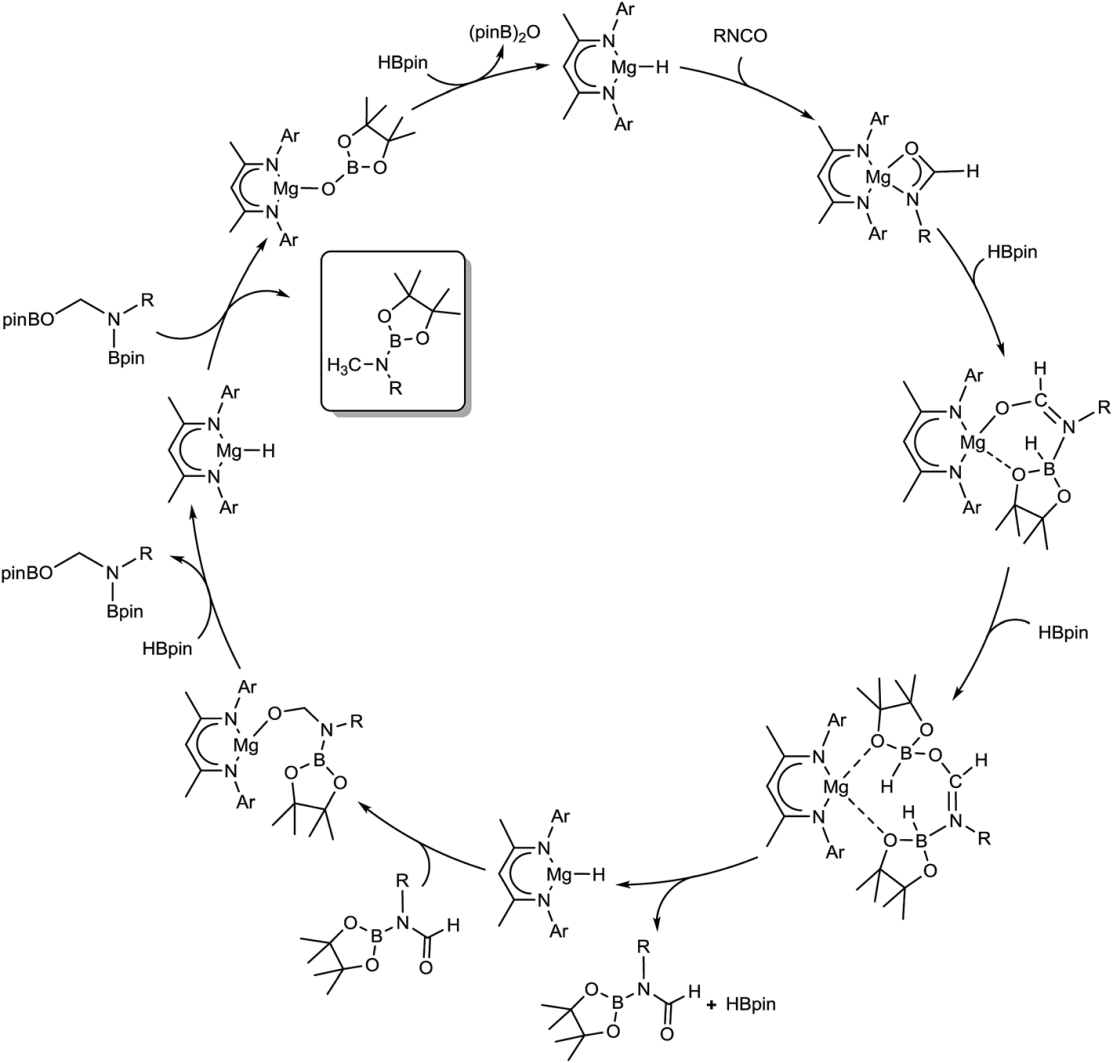
Proposed mechanism for the magnesium-catalysed hydrodeoxygenation of organic isocyanates.

## Conclusions

In conclusion, we describe a mild protocol for the catalytic transformation of the isocyanate function to a methyl amine. The activation of the heterocumulene occurs through a magnesium-centered hydroboration and ultimately the complete cleavage of the C=O bond. The sequential nature of this reactivity indicates that it may be further generalised to the targeted cleavage of the C–O bonds in alternative molecular and, potentially, even macromolecular species. We are continuing to study these possibilities.

## Supplementary Material

Supplementary information

Crystal structure data
