# Supplementary material for: Hydrodeoxygenation of isocyanates: snapshots of a magnesium-mediated C[double bond, length as m-dash]O bond cleavage
Source: Chem Sci. 2017 Mar 1;8(5):3529–37. doi: 10.1039/c7sc00117g (PMC6092714; doi:10.1039/c7sc00117g)
Supplement: Supplementary file 1 [file SC-008-C7SC00117G-s001.pdf]

Electronic Supplementary Material (ESI) for Chemical Science.
This journal is © The Royal Society of Chemistry 2017

Supplementary Information for

Hydrodeoxygenation of Isocyanates: Snapshots of a Magnesium-mediated C=O Bond Cleavage

Yan Yang,[a] Mathew D. Anker,[b] Jian Fang,[a]* Mary F. Mahon,[b] Laurent Maron,[c] Catherine Weetman[b] and Michael S. Hill[b]*

[a] *Key Laboratory of Nonferrous Metal Chemistry and Resources Utilization of Gansu Province School of Chemistry and Chemical Engineering, Lanzhou University, Lanzhou 730000, PR China.*

[b]*Department of Chemistry, University of Bath, Bath BA2 7AY, UK*

[c] *LPCNO, Université de Toulouse, INSA Toulouse 135, Avenue de Rangueil, 31077 Toulouse cedex, France*

General Experimental Procedures

All manipulations were carried out using standard Schlenk line and glovebox techniques under an inert atmosphere of argon. NMR experiments were conducted in J. Youngs tap NMR tubes made up and sealed in a Glovebox. NMR spectra were recorded on a Bruker AV300 spectrometer operating at 300.2 MHz (^{1}H), 75.5 MHz (^{13}C), 96.3 MHz (^{11}B). The spectra were referenced relative to residual solvent resonances or an external $BF_3.OEt_2$ standard (^{11}B). Microanalysis was performed by Mr S. Boyer of London Metropolitan Enterprises. Solvents (toluene, THF, hexane) were dried by passage through a commercially available (Innovative Technologies) solvent purification system, under nitrogen and stored in ampoules over molecular sieves. C_6D_6 and d_8-toluene were purchased from Fluorochem Ltd. and dried over molten potassium before distilling under nitrogen and storing over molecular sieves. Di-*n*-butylmagnesium (1.0 M solution in n-heptane) and pinacolborane were purchased from Sigma-Aldrich Ltd. and used without further purification. Organic isocyanates were purchased from the same supplier and dried over P_2O_5 before either distillation or vacuum transfer. [HC{(Me)CN(2,6-$^{i}Pr_2C_6H_3$)}$_2$Mg*n*Bu] (**1**) and [HC{(Me)CN(2,6-$^{i}Pr_2C_6H_3$)}$_2$MgH]$_2$ were synthesized by literature procedures.[1,2]

Synthesis of [(HC{(Me)CN(2,6-$^{i}PrC_6H_3$)}$_2$MgOC(H)N(2,6-$^{i}PrC_6H_3$)], 2

Addition of 2,6-di-isopropylphenyl isocyanate (0.067 mmol), to a J Youngs NMR tube containing a solution of [HC{(Me)CN(2,6-$^{i}Pr_2C_6H_3$)}$_2$MgH]$_2$, generated by addition of HBpin (0.067 mmol) to a solution of compound **1** (0.067 mmol), in d_8-toluene resulted in the immediate formation of compound **2**. Volatiles were removed *in vacuo* and the compound was redissolved in hexane. Storage at –35°C overnight yielded compound **2** as colourless needles suitable for single crystal X-ray characterisation (76%). ^{1}H NMR (d_8-tol, 300 MHz): δ 7.92 (1H, s, NC(*H*)O), 7.06 (6H, bs, Ar-*H*), 6.90 (3H, s, Ar-*H*), 4.96 (1H, s, C-*H*), 3.44 (2H, bm, C*H*(CH_3)$_2$), 3.17 (2H, bm, C*H*(CH_3)$_2$), 1.98 (2H, m, $^{3}J_{HH}$ = 6.78 Hz, C*H*(CH_3)$_2$), 1.66 (6H, s, NC(C*H*$_3$)CH), 1.33 (6H, bm, CH(C*H*$_3$)$_2$), 1.11 (12H, d, $^{3}J_{HH}$ = 6.78 Hz, CH(C*H*$_3$)$_2$), 0.96 (6H, bm, CH(C*H*$_3$)$_2$), 0.80 (6H, d, $^{3}J_{HH}$ = 6.78 Hz, CH(C*H*$_3$)$_2$). ^{13}C{^{1}H} NMR (d_8-tol, 125.76 MHz): δ 175.0 (N*C*(H)O),169.7 (N*C*(CH_3)CH), 143.9, 143.5, 140.3, 125.95, 125.40, 124.3, 122.9 (*C*-Ar), 95.1 (NC(CH_3)*C*H), 28.1, 27.8 (N*C*H(CH_3)$_2$), 24.7, 23.8 (CH(*C*H$_3$)$_2$). Despite multiple attempts, a meaningful microanalysis could not be obtained for this compound.

Figure S1: ^{1}H NMR spectrum of compound **2**.

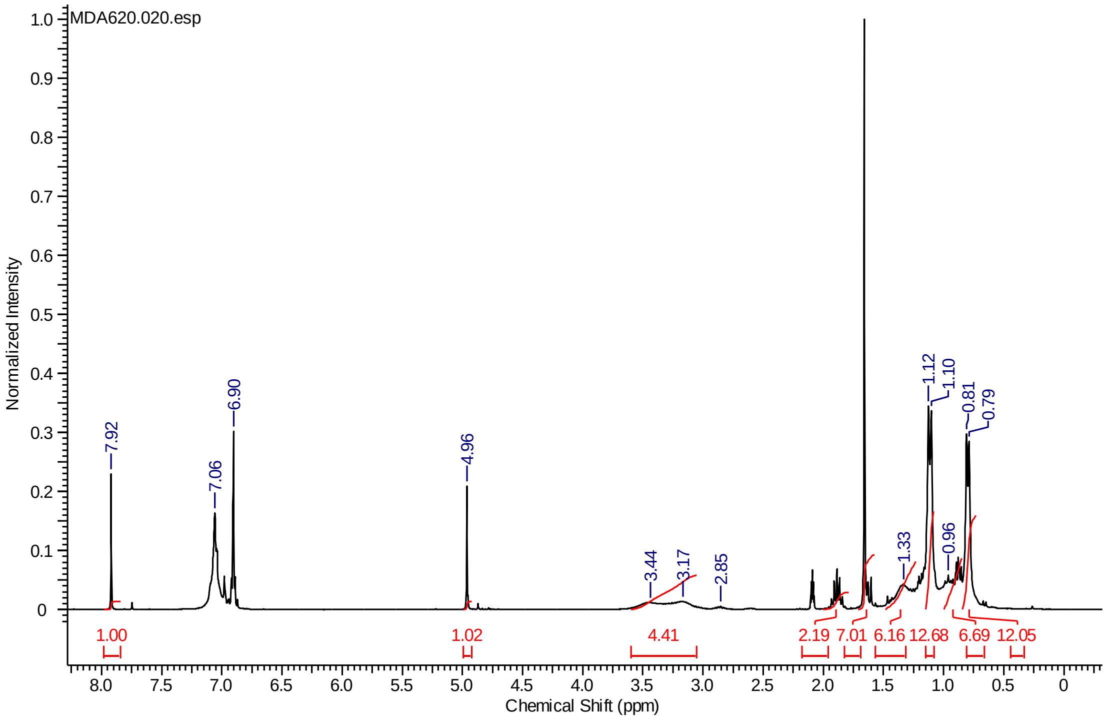

Figure S2: ^{13}C{^{1}H} NMR spectrum of compound **2**.

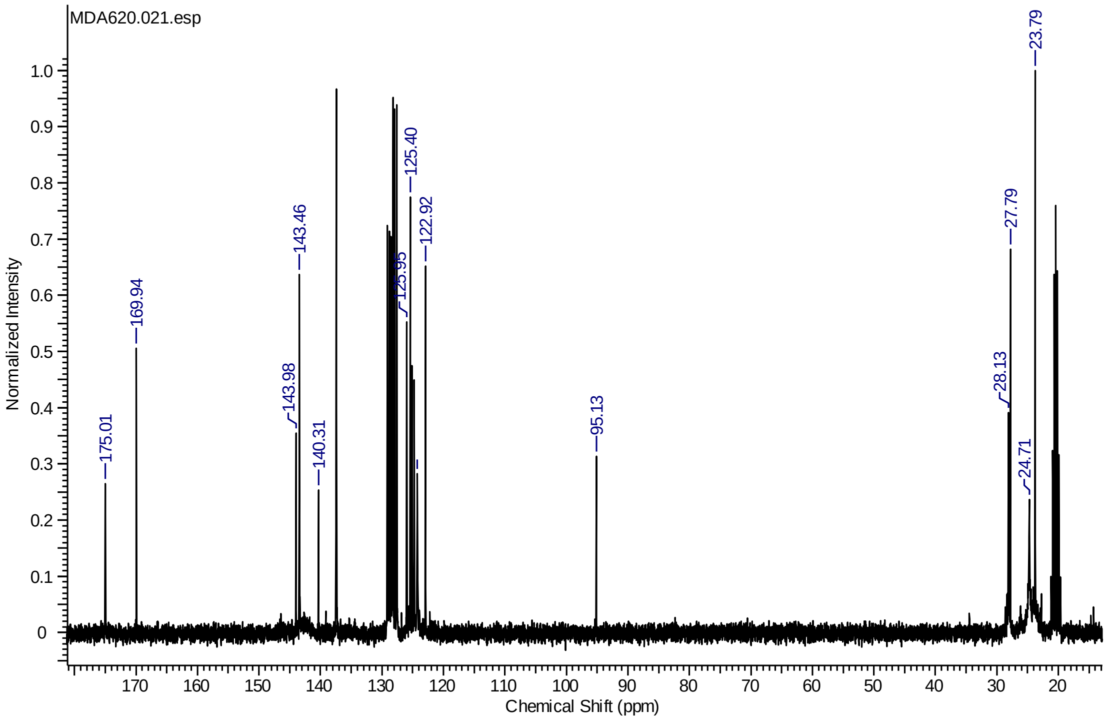

Synthesis of [(HC{(Me)CN(2,6-^{i}PrC$_6$H$_3$)}$_2$MgOC(H)N(2,6-^{i}PrC$_6$H$_3$)(HBpin)], 3

Addition of 4,4,5,5,-tetramethyl-1,3,2-dioxaborolane (0.067 mmol), to a J Youngs NMR tube containing a solution of compound **2** (0.067 mmol) in toluene resulted in the immediate formation of compound **3**. Solvent was removed *in vacuo* and the solid was redissolved in hexane. Storage at –35°C overnight provided compound **3** as colourless crystalline blocks suitable for single crystal X-ray diffraction analysis (70%). ^{1}H NMR (d$_8$-tol, 280K, 400 MHz): δ 7.69 (1H, s, NC(*H*)O), 7.14 (6H, bm, Ar-*H*), 7.03 – 6.98 (3H, bs, Ar-*H*), 4.92 (1H, s, C-*H*), 3.40 (3H, m, $^3J_{HH}$ = 6.82 Hz, C*H*(CH$_3$)$_2$), 3.28 (1H, m, $^3J_{HH}$ = 6.06 Hz, C*H*(CH$_3$)$_2$), 3.15 (2H, spt, $^3J_{HH}$ = 6.82 Hz, C*H*(CH$_3$)$_2$), 1.74 (6H, s, NC(C*H*$_3$)CH), 1.37 (10H, bm, CH(C*H*$_3$)$_2$), 1.26 (10H, bm, CH(C*H*$_3$)$_2$), 1.20 (6H, bm, CH(C*H*$_3$)$_2$), 1.11 (10H, bm, CH(C*H*$_3$)$_2$), 0.79 (6H, s, OC(C*H*$_3$)$_2$), 0.58 (3H, s, OC(C*H*$_3$)$_2$), 0.47 (3H, s, OC(C*H*$_3$)$_2$). ^{11}B NMR (d$_8$-tol, 125.76 MHz): δ 4.3 (H*B*Pin). Anal. Calcd. for C$_{48}$H$_{73}$MgBN$_3$O$_3$: C, 74.37; H, 9.49; N, 5.42%; Found: C, 74.36; H, 9.13; N, 5.32%.

Figure S3: ^{1}H NMR spectrum of compound **3**.

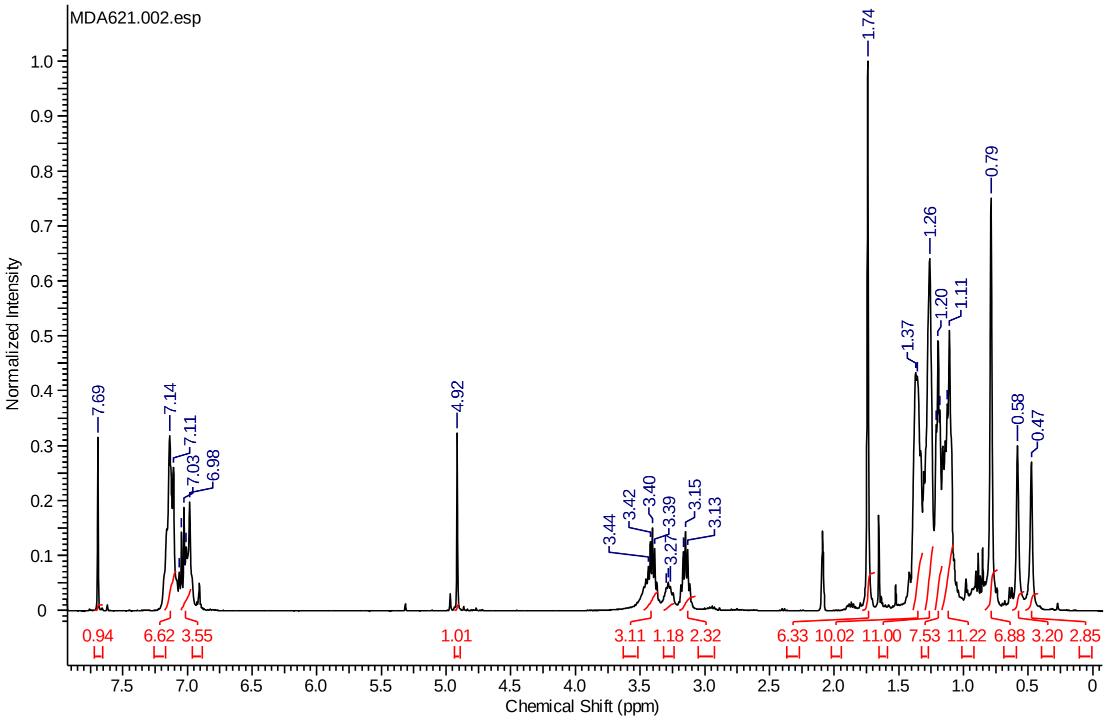

Figure S4: $^{13}C\{^1H\}$ NMR spectrum of compound **3**. Expansion of aliphatic region below.

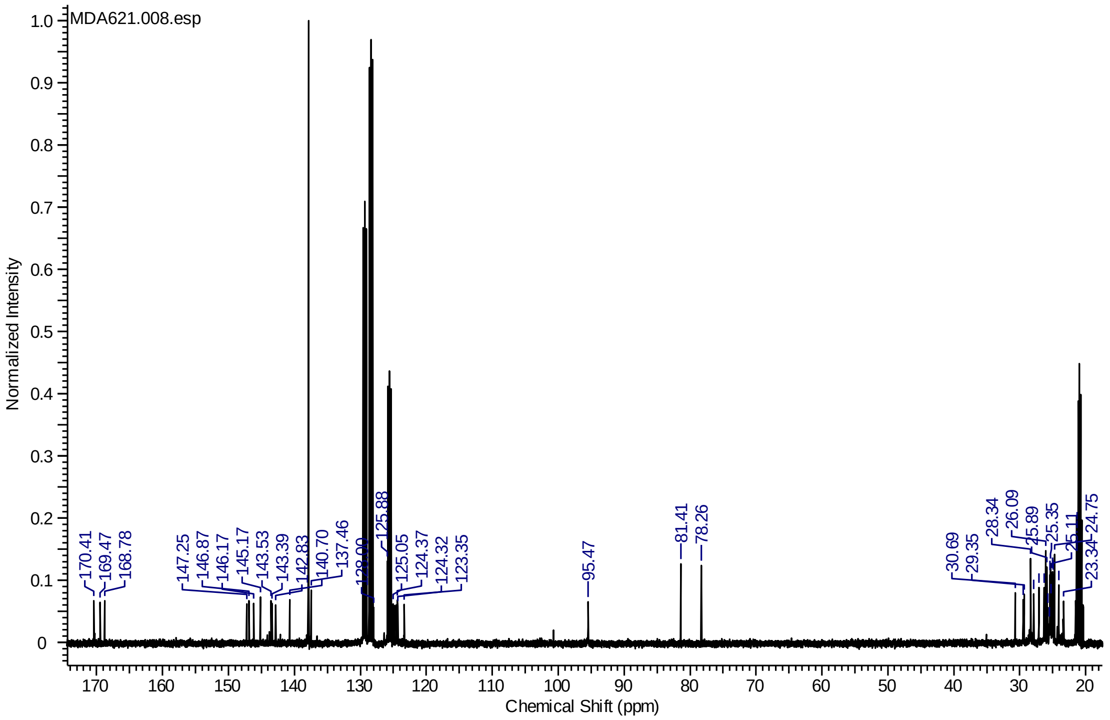

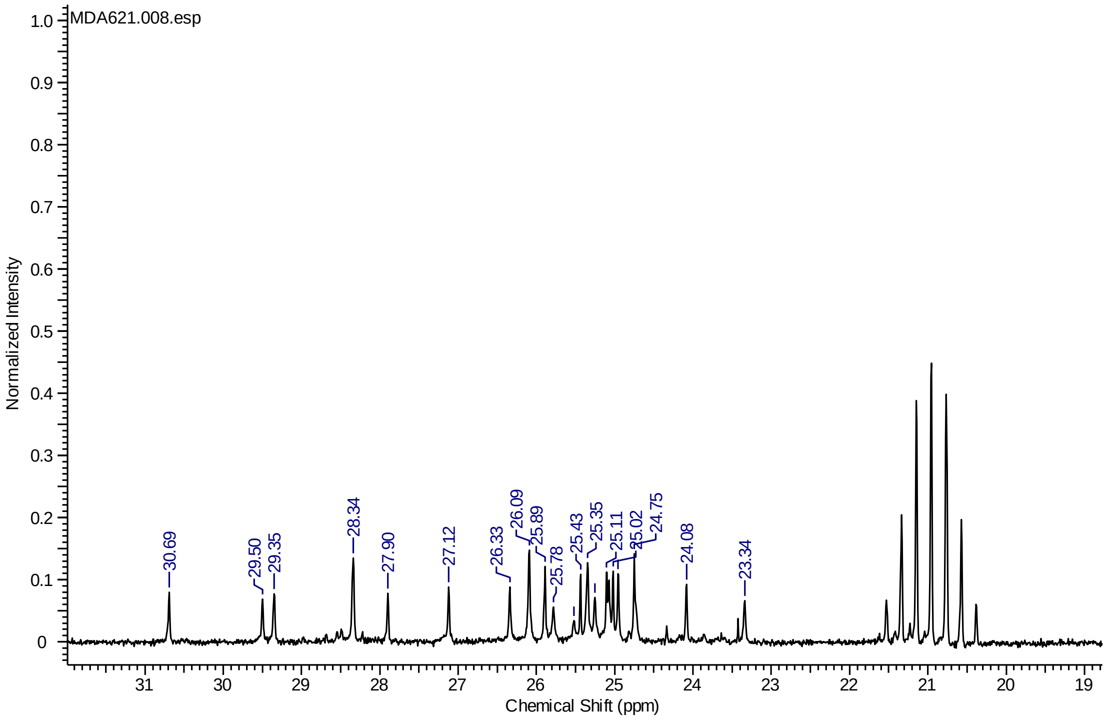

Figure S5: ^{11}B NMR spectrum of compound **3**.

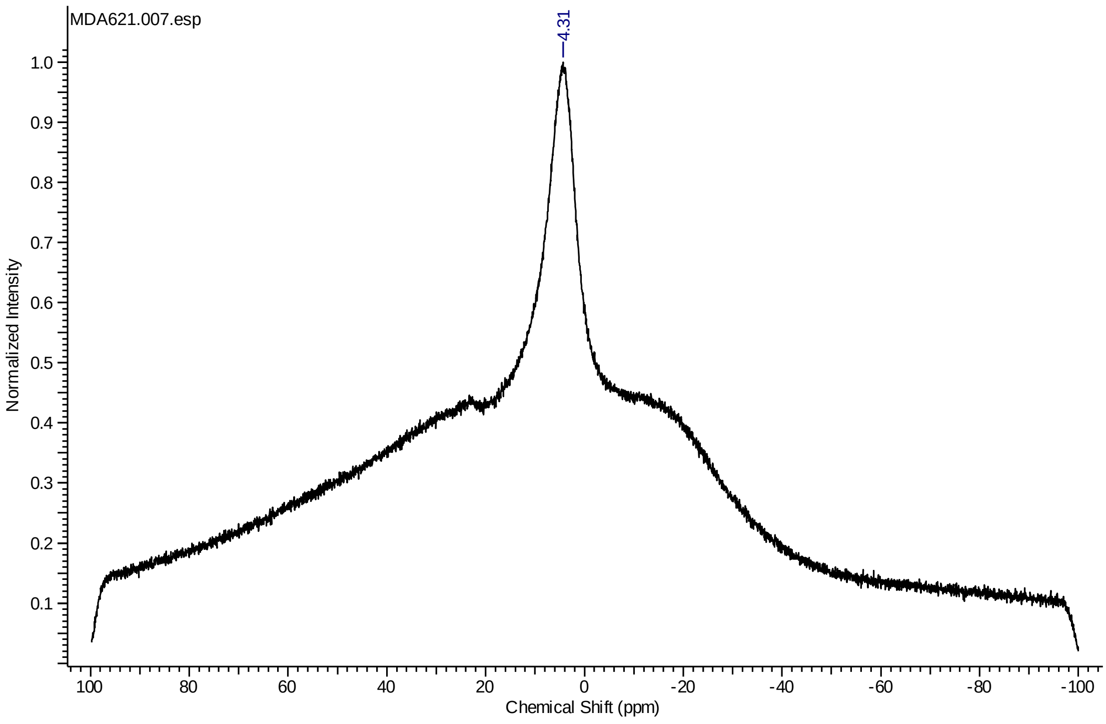

Synthesis of [(HC{(Me)CN(2,6-^{i}PrC$_6$H$_3$)}$_2$MgOBpin)], 4

Addition of 2,6-Diisopropylpheynyl isocyanate (0.067 mmol), to a J Youngs tube containing a solution of compound **1** (0.067 mmol) and 4,4,5,5,-tetramethyl-1,3,2-dioxaborolane (0.201 mmol), in toluene. The reaction was heated at 60°C for 24 hours and compound **4** crystallised from the reaction solution as colourless blocks suitable for single crystal X-ray diffraction analysis (85%). Once crystallised, compound **4** displayed only sparing solubility in arene solvents necessitating analysis by NMR spectroscopy in d$_8$-THF. ^{1}H NMR (d$_8$-THF, 300 MHz): δ 7.14 – 7.03 (4H, m, Ar-*H*), 6.91 (1H, d, $^3J_{HH}$ = 3.01 Hz, Ar-*H*), 6.89 (1H, d, $^3J_{HH}$ = 3.01 Hz, Ar-*H*), 4.79 (1H, s, C-*H*), 3.58 (THF), 3.30 (2H, m., $^3J_{HH}$ = 6.78 Hz, C*H*(CH$_3$)$_2$), 3.04 (2H, m., $^3J_{HH}$ = 6.78 Hz, C*H*(CH$_3$)$_2$), 1.73 (THF), 1.37 (6H, s, NC(C*H*$_3$)CH), 1.23 (6H, d, $^3J_{HH}$ = 6.78 Hz, CH(C*H*$_3$)$_2$), 1.18 (6H, s, C(C*H*$_3$)$_2$), 1.11 (6H, d, $^3J_{HH}$ = 6.78 Hz, CH(C*H*$_3$)$_2$), 0.98 (6H, d, $^3J_{HH}$ = 6.78 Hz, CH(C*H*$_3$)$_2$), 0.97 (6H, d, $^3J_{HH}$ = 6.78 Hz, CH(C*H*$_3$)$_2$), 0.07 (6H, s, C(C*H*$_3$)$_2$), 0.05 (6H, s, C(C*H*$_3$)$_2$). ^{13}C{^{1}H} NMR (d$_8$-THF, 125.76 MHz): δ 169.7 (N*C*(CH$_3$)CH), 148.4, 144.5, 143.3, 125.9, 124.7, 124.0 (*C*-Ar), 97.4 (NC(CH$_3$)*C*H), 82.4 (O*C*(CH$_3$)$_2$), 29.5, 28.6 (N*C*H(CH$_3$)$_2$), 27.1, 26.9 ((OC(*C*H$_3$)$_2$), 24.4, 23.4(CH(*C*H$_3$)$_2$). ^{11}B NMR (d$_8$-THF, 125.76 MHz): δ 21.6 O*B*Pin. Anal. Calcd. for $C_{70}H_{106}B_2Mg_2N_4O_6$: C, 71.87; H, 9.13; N, 4.79%; Found: C, 71.92; H, 8.93; N, 4.56%.

Figure S6: ^{1}H NMR spectrum of compound **4**.

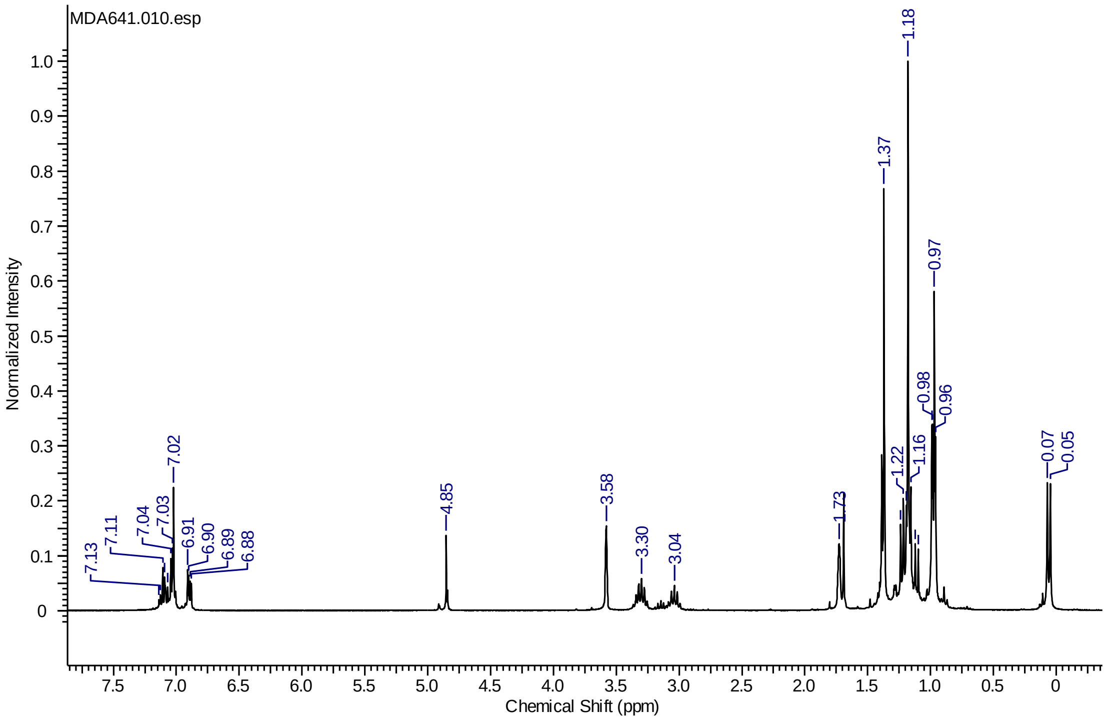

Figure S7: ^{11}B NMR spectrum of compound **4**.

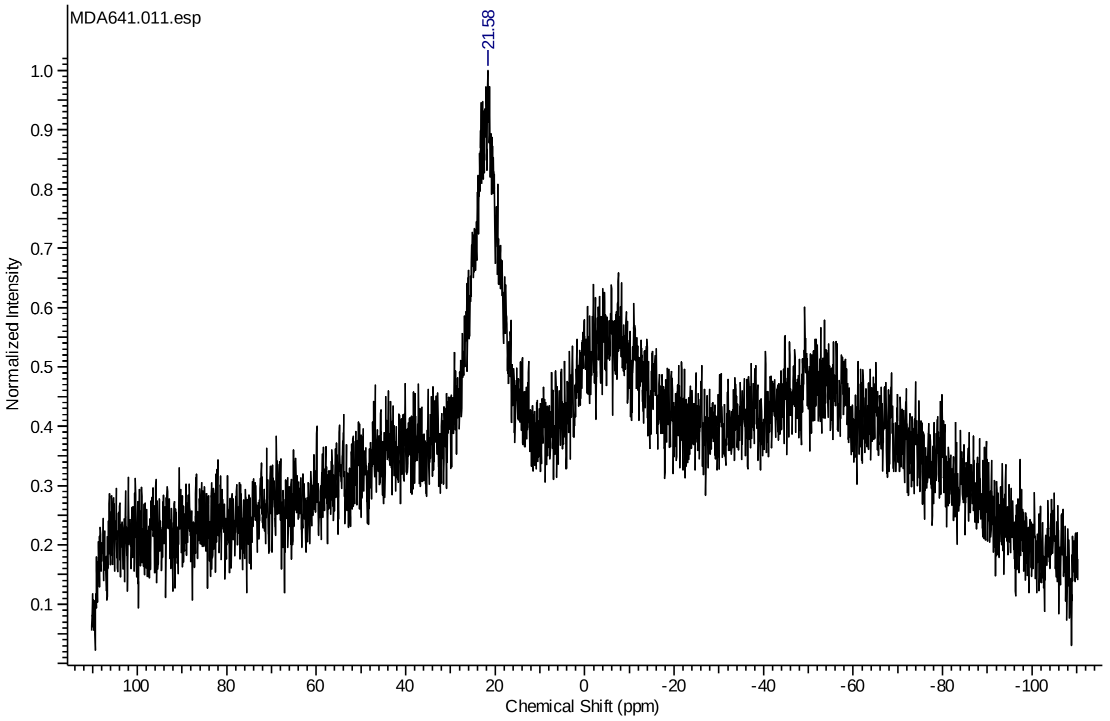

Figure S8: $^{13}C\{^{1}H\}$ NMR spectrum of compound **4**.

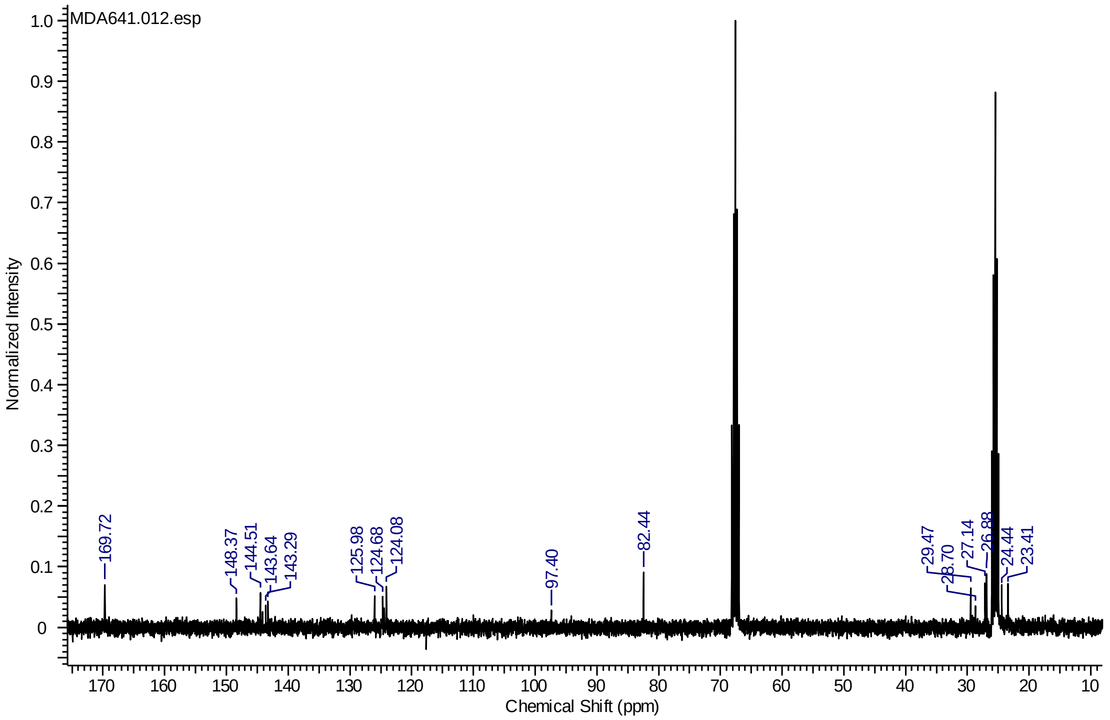

Figure S9: ^{1}H NMR spectrum of a non-catalysed reaction in C_6D_6 of ^{i}PrNCO and 3equivalents of HBpin after 12 hours at 60 °C.

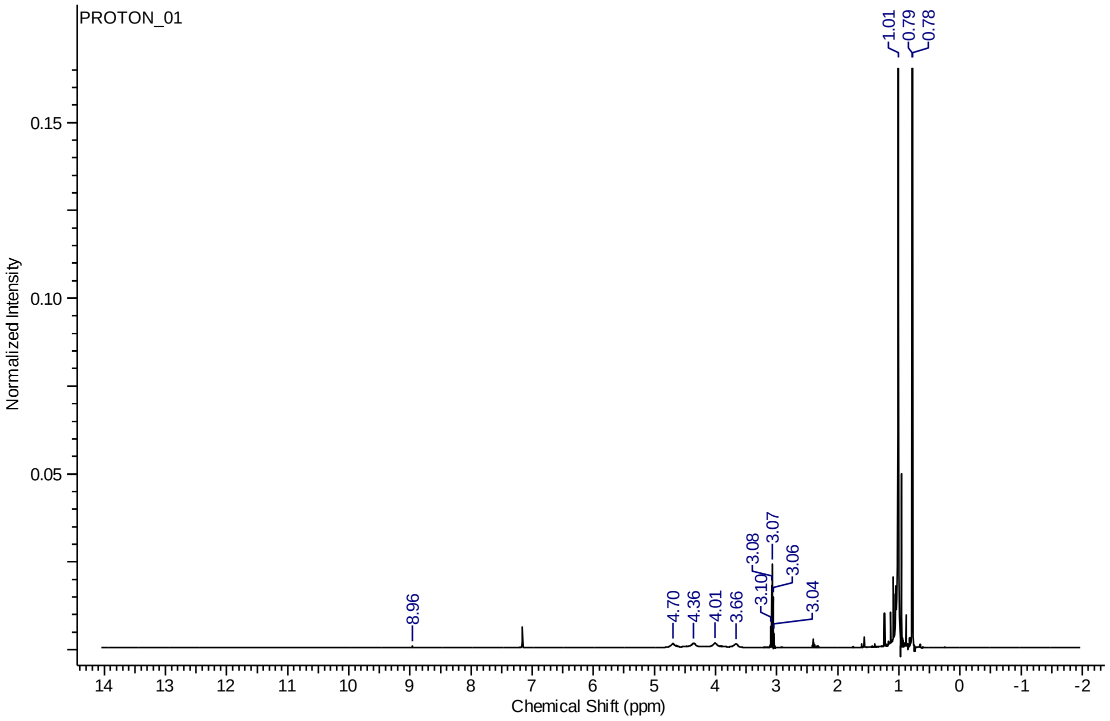

Figure S10: ^{11}B NMR spectrum of a non-catalysed reaction in C_6D_6 of ^{i}PrNCO and 3equivalents of HBpin after 12 hours at 60 °C.

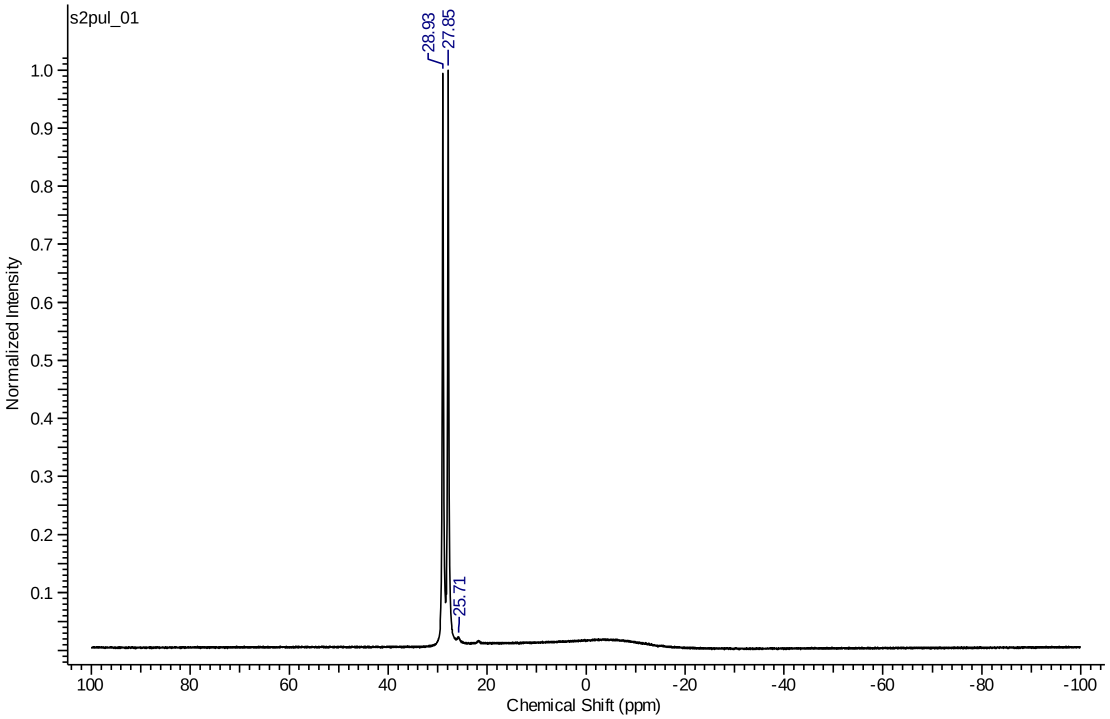

Figure S11: ^{1}H NMR spectrum of a non-catalysed reaction in C_6D_6 of DippNCO and 3equivalents of HBpin after 12 hours at 60 °C.

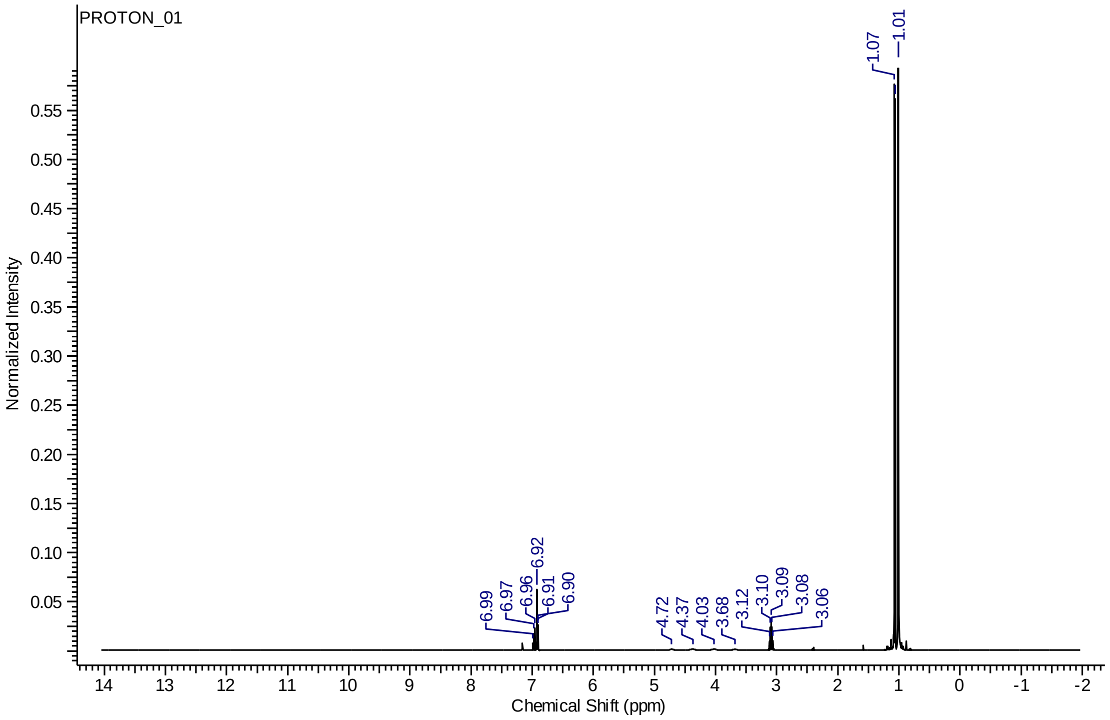

Figure S12: Expansion of ^{1}H NMR spectrum of a non-catalysed reaction in C_6D_6 of DippNCO and 3equivalents of HBpin after 12 hours at 60 °C.

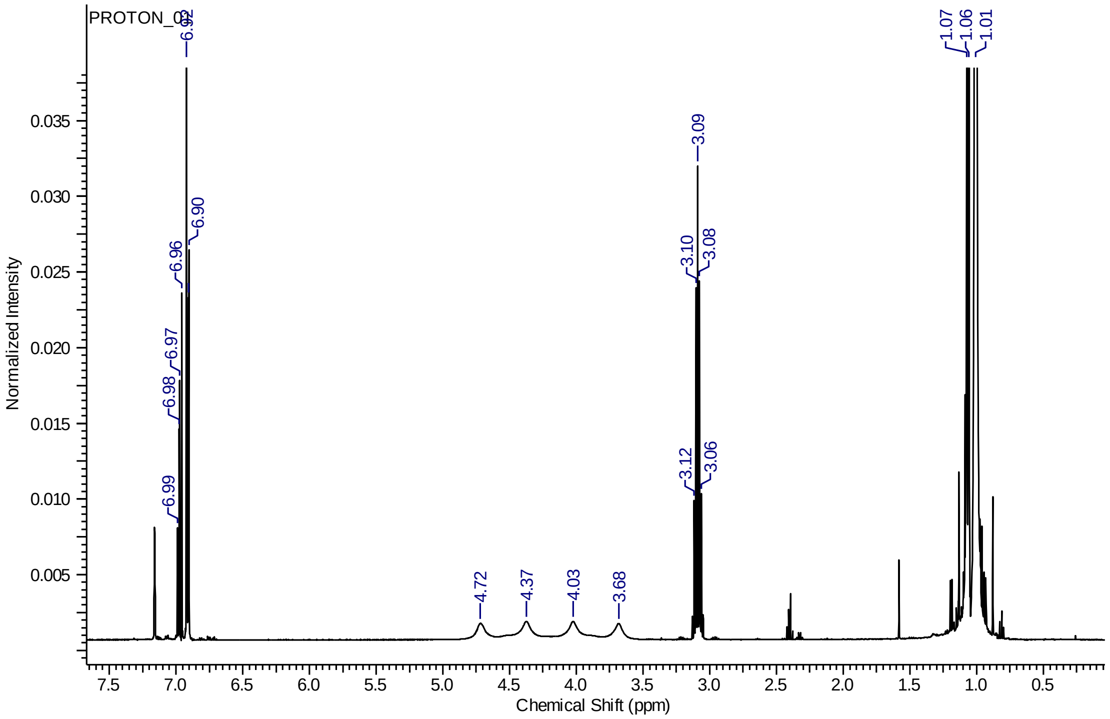

Figure S13: ^{11}H NMR spectrum of a non-catalysed reaction in C_6D_6 of DippNCO and 3equivalents of HBpin after 12 hours at 60 °C.

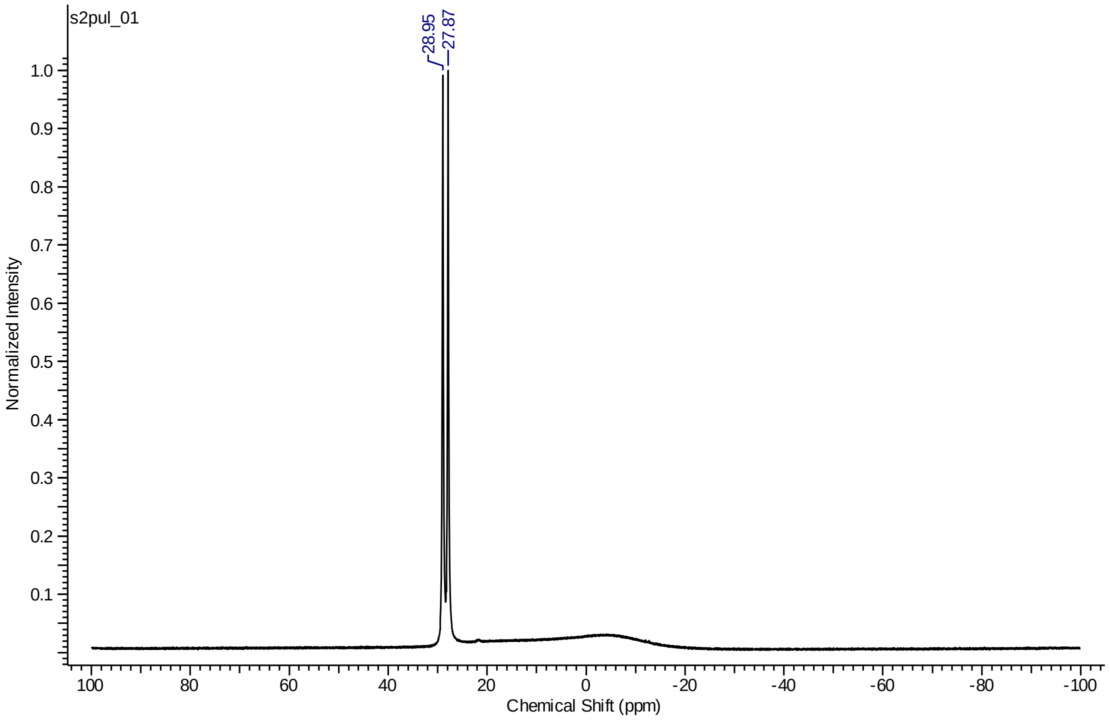

Figure S14: ^{1}H NMR spectrum of a non-catalysed reaction in C_6D_6 of AdNCO and 3equivalents of HBpin after 12 hours at 60 °C.

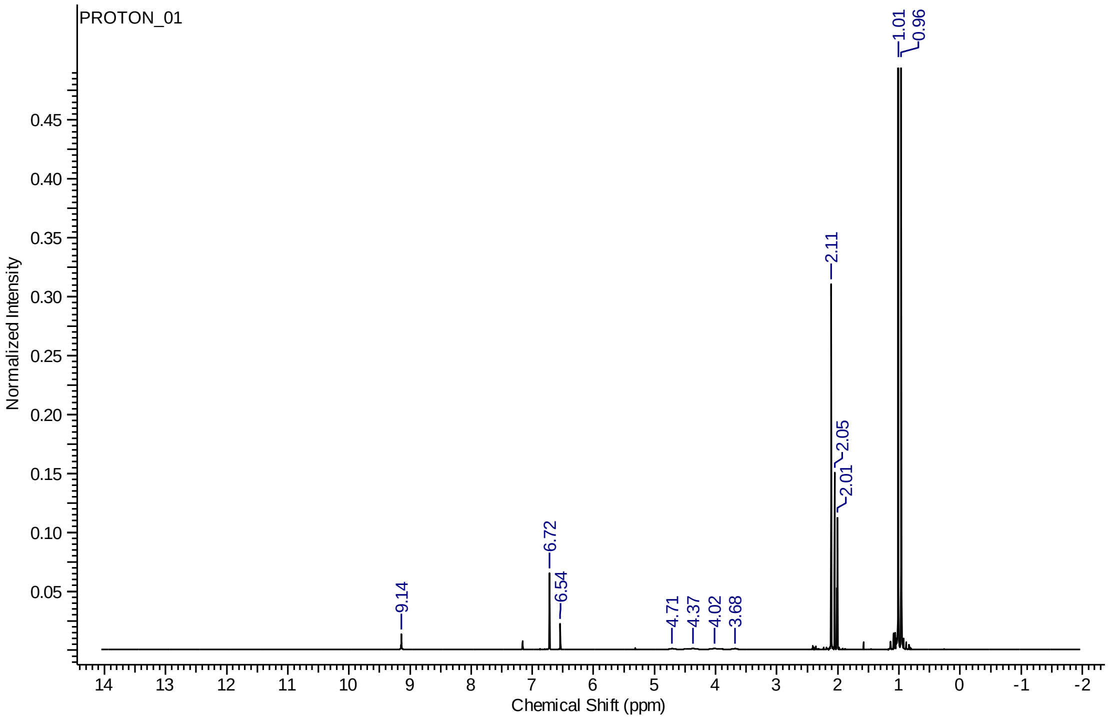

Figure S15: Expansion of ^{1}H NMR spectrum of a non-catalysed reaction in C_6D_6 of AdNCO and 3equivalents of HBpin after 12 hours at 60 °C.

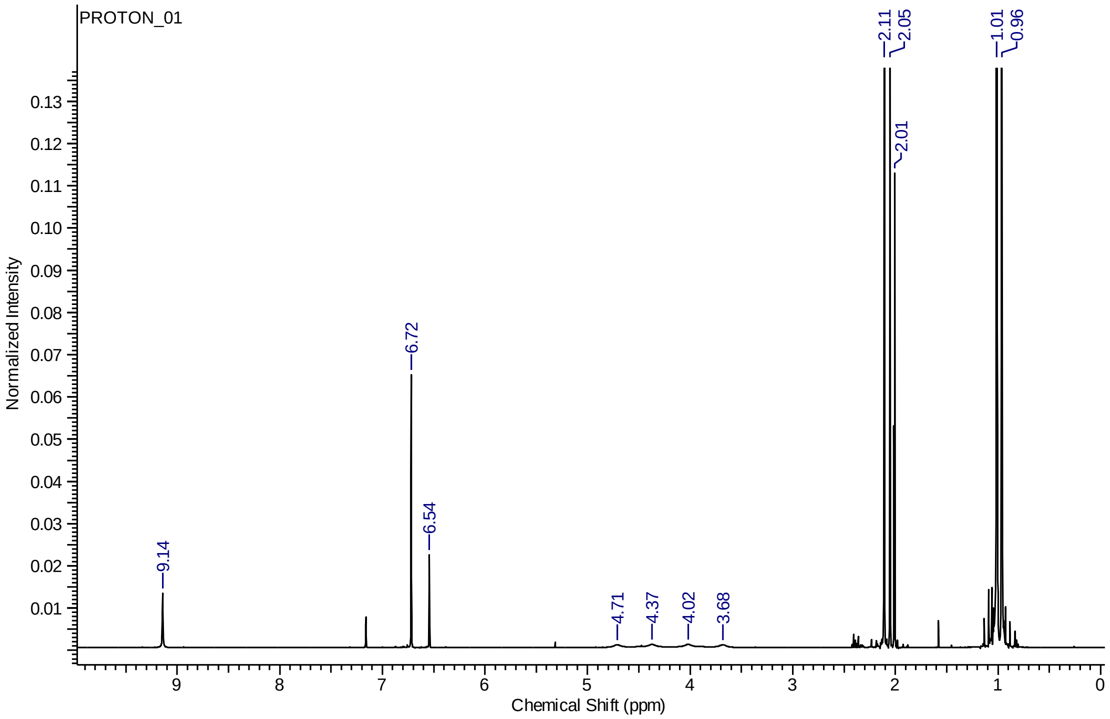

Figure S16: ^{11}B NMR spectrum of a non-catalysed reaction in C_6D_6 of AdNCO and 3equivalents of HBpin after 12 hours at 60 °C.

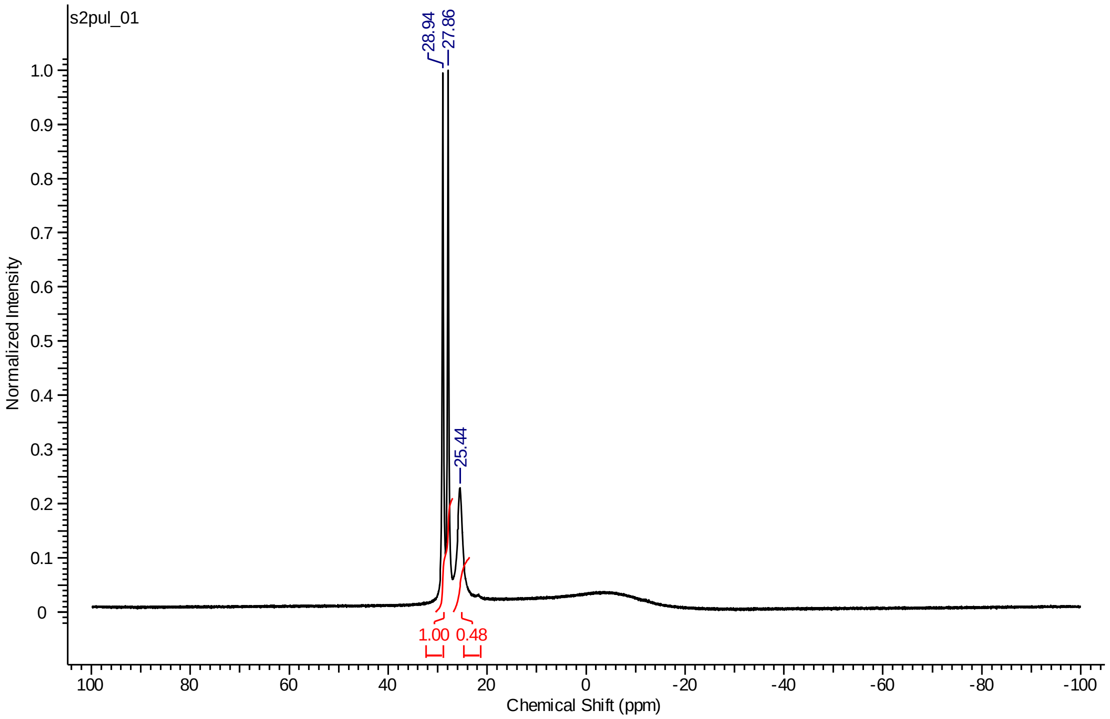

Catalytic Hydroboration of Isocyanates General Procedure: 10 mg (0.02 mmol, i.e. 10 mol%) of **1** was dissolved in 0.5 ml of C_6D_6. 90.0 μL (0.62 mmol) of pinacolborane was then added followed by either 0.2 or 0.4 mmol of the relevant isocyanate. This mixture was then transferred to a sealed Youngs tap NMR tube and the reaction was either allowed to react at room temperature or was heated in an oil bath at 60 ^{o}C for the reaction times shown in Tables 1 and 2. These reactions were regularly monitored by ^{1}H and ^{11}B NMR spectroscopy until maximum conversion was observed. On exposure to ambient atmosphere or on deliberate addition of a drop of aqueous dilute HCl the *N*-borylated amines were observed to hydrolyse rapidly to liberate the protonated parent methyl amines.

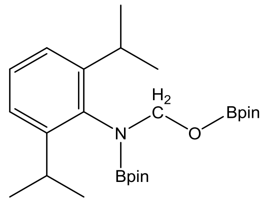

Synthesis of N,O-{B(OCMe$_2$)$_2$}$_2$-(methoxy)-2,6-diisopropylphenylamine, 5. Synthesis performed using 42.8 µL of 2,6-diisopropylphenyl isocyanate. ^{1}H NMR (400 MHz, C_6D_6, 300 K) δ_H (ppm): 7.14 (1H, t, J_{HH} = 4 Hz, p-H), 7.10 (2H, d, J_{HH} = 4 Hz, m-H), 5.35 (2H, s, NCH_2O), 3.55 (2H, sept, J_{HH} = 8 Hz, $CH(CH_3)_2$), 1.39 (6H, d, J_{HH} = 8 Hz, $CH(CH_3)_2$), 1.29 (6H, d, J_{HH} = 8 Hz, $CH(CH_3)_2$), 0.99 (O-B(OC(CH$_3$)$_2$)$_2$), 0.92 (N-B(OC(CH$_3$)$_2$)$_2$). ^{13}C{^{1}H} NMR (125.8 MHz, C_6D_6, 300 K) δ_C (ppm): 155.3 (ipso-C), 148.2 (o-C), 137.2 (p-C), 123.6 (m-C), 83.3 (O-B(OC(CH$_3$)$_2$)$_2$), 82.6 (N-B(OC(CH$_3$)$_2$)$_2$), 77.9 (NCH_2O), 28.9 ($CH(CH_3)_2$), 25.3 ($CH(CH_3)_2$), 25.0 (O-B(OC(CH$_3$)$_2$)$_2$), 24.9 (N-B(OC(CH$_3$)$_2$)$_2$). ^{11}B NMR (96.3 MHz, C_6D_6, 300K) δ_B (ppm): 28.3 (br. s, NB), 24.8 (br. s. OB). Anal. Calcd. for $C_{25}H_{43}B_2NO_5$: C, 65.39; H, 9.44; N, 3.05%; Found: C, 65.33; H, 9.47; N, 3.14%.

Figure S17: ^{1}H NMR spectrum of compound **5** resulting from the hydroboration of DippNCO with HBpin catalysed by 10 mol% **1**.

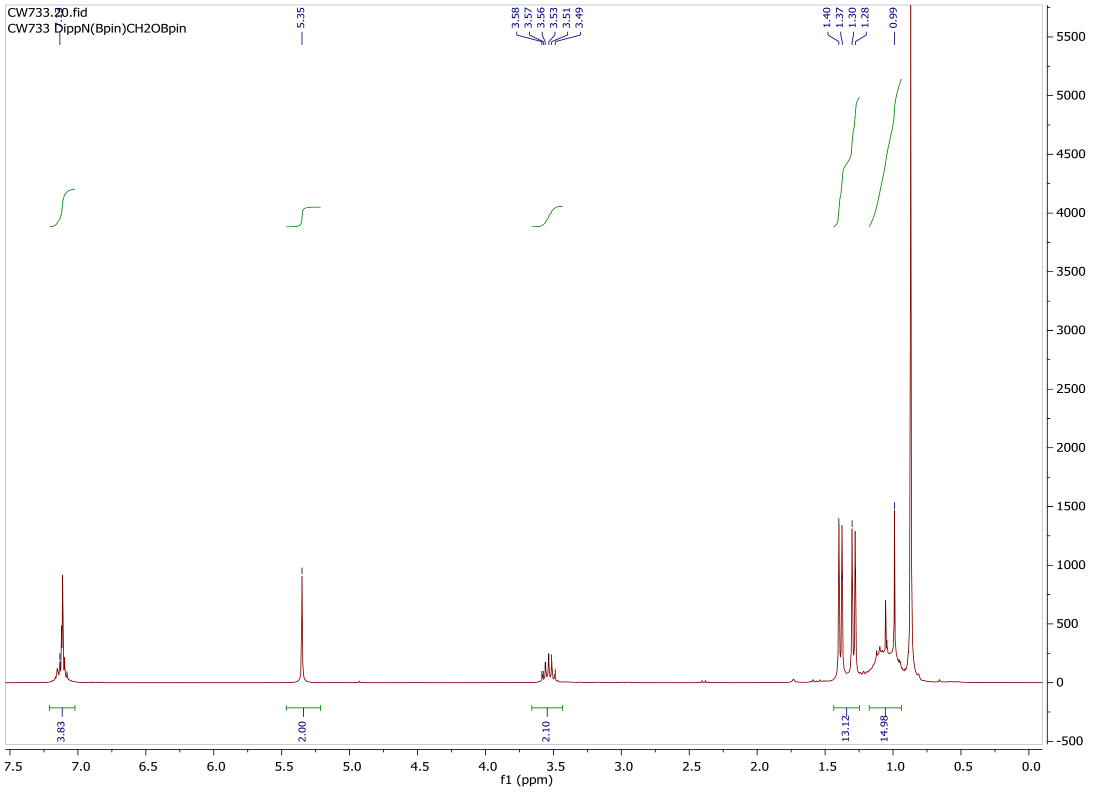

Figure S18: $^{13}C\{^{1}H\}$ NMR spectrum of compound **5** resulting from the hydroboration of DippNCO with HBpin catalysed by 10 mol% **1**.

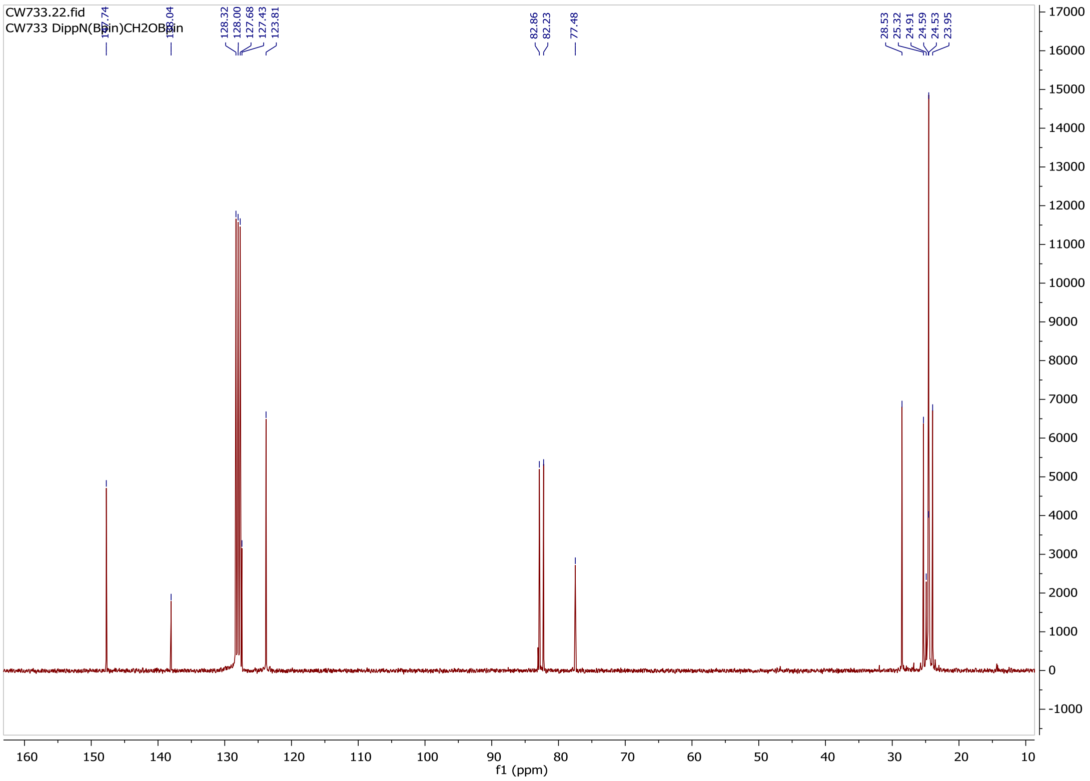

Synthesis of N,O-{B(OCMe$_2$)$_2$}$_2$-(methoxy)-2,4,6-trimethylphenylamine, 6.

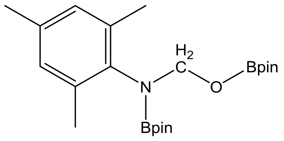

32.2 mg of 2,4,6-trimethylphenyl isocyanate. Compound **6** was isolated by crystallization from the reaction solvent at room temperature (65%). ^{1}H NMR (d$_8$-tol, 300 MHz): δ 6.75 (2H, s, Ar-*H*), 5.29 (2H, s, C*H*$_2$), 2.37 (6H, s, Ar-*o*-C*H*$_3$), 2.10 (3H, s, Ar-para-CH$_3$), 0.91 (12H, s, OC(C*H*$_3$)$_2$). ^{13}C{^{1}H} NMR (d$_8$-tol, 125.76 MHz): δ 139.7, 137.1, 135.6, 129.7 (*C*-Ar), 83.0, 82.5 (O*C*(CH$_3$)$_2$), 76.9 (N*C*H$_2$O), 24.9 (Ar-*C*H$_3$), 24.7 ((OC(*C*H$_3$)$_2$), 19.3 (Ar-*C*H$_3$). ^{11}B NMR (d$_8$-tol, 125.76 MHz): δ 27.2 (N*B*Pin), 25.3 (O*B*Pin). Anal. Calcd. for C$_{22}$H$_{41}$B$_2$NO$_7$: C, 58.31; H, 9.12; N, 3.36%; Found: C, 58.58; H, 8.89; N, 3.30%.

Figure S19: ^{1}H NMR spectrum of compound **6**.

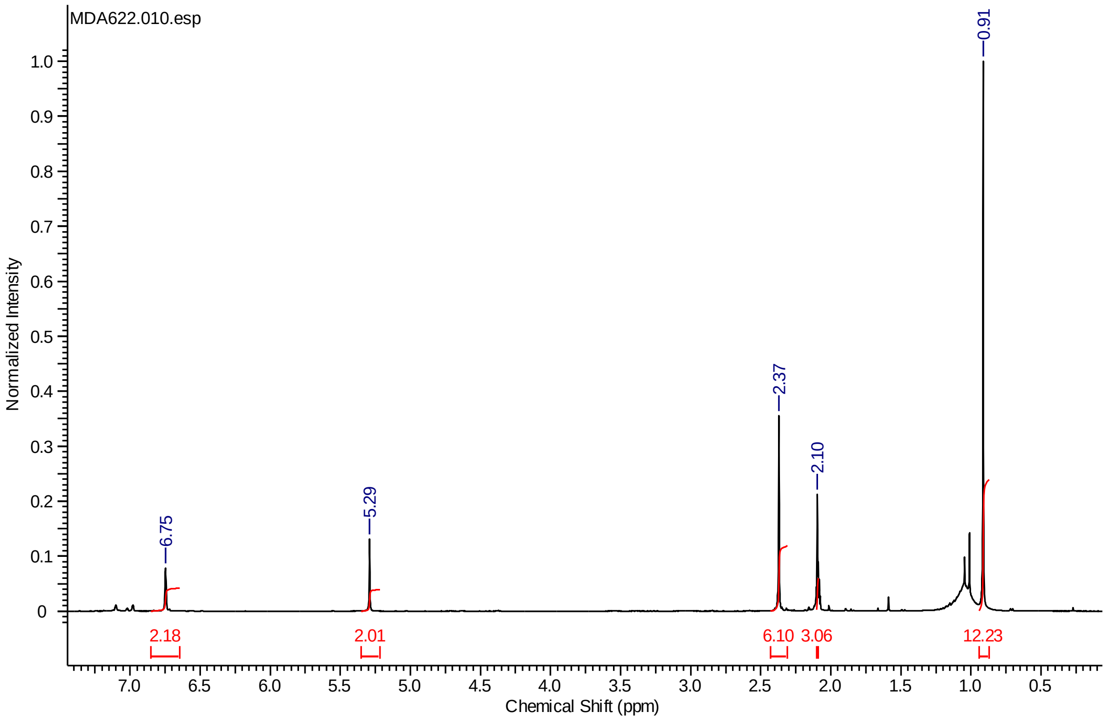

Figure S20: ^{11}B NMR spectrum of compound **6**.

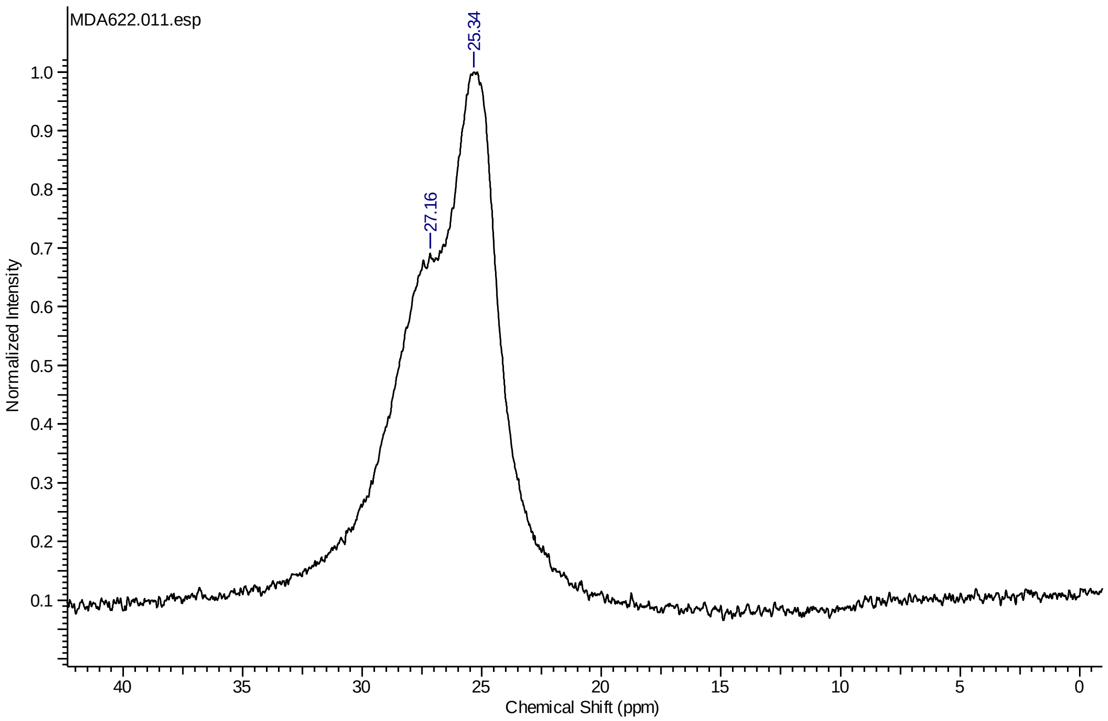

Figure S21: ^{13}C{^{1}H} NMR spectrum of compound **6**.

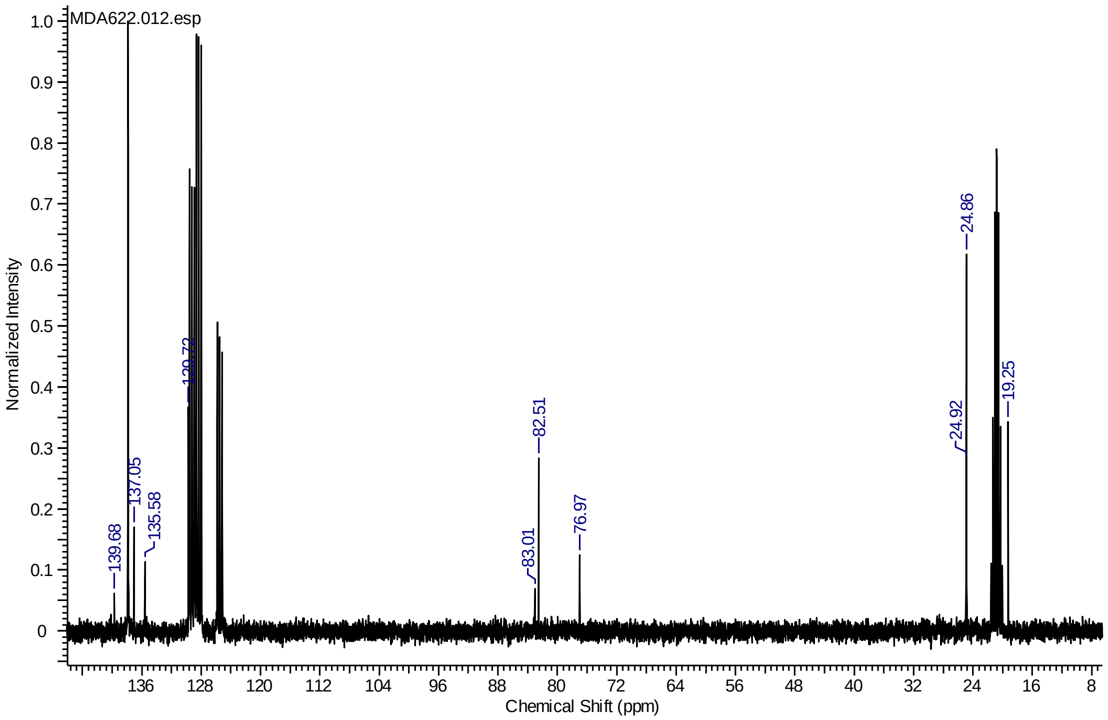

Synthesis of [*N*-(2,6-^{i}PrC$_6$H$_3$)(Bpin)CH(O)], 7.

Addition of 2,6-Diisopropylpheynyl isocyanate (2.0 mmol), to a J Youngs tube containing a solution of compound **1** (0.02 mmol) and 4,4,5,5,-tetramethyl-1,3,2-dioxaborolane (2.0 mmol), in 0.5 ml of toluene. The reaction was left at room temperature 5 minutes and compound **7** crystallised from the reaction solution as colourless needles suitable for single crystal X-ray diffraction analysis (85%). ^{1}H NMR (d$_8$-tol, 300 MHz): δ 9.13 (1H, s, O*H*C), 7.14 – 7.05 (3H, m, Ar-*H*), 2.98 (2H, spt, $^{3}J_{HH}$ = 6.85 Hz, C*H*(CH$_3$)$_2$), 1.21, 1.19 (6H, d, $^{3}J_{HH}$ = 6.85 Hz, CH(C*H*$_3$)$_2$), 0.96 (12H, s, Bpin). ^{13}C{^{1}H} NMR (d$_8$-tol, 125.76 MHz): δ 164.8 (OHC), 146.2, 128.8, 124.0 (*C*-Ar), 84.5 (O*C*(CH$_3$)$_2$),) 29.6 (*C*H(CH$_3$)$_2$), 24.7 (OC(*C*H$_3$)$_2$), 24.6, 24.4 (CH(*C*H$_3$)$_2$). ^{11}B NMR (d$_8$-tol, 125.76 MHz): δ 25.6 (O*B*Pin). Anal. Calcd. for C$_{19}$H$_{30}$BNO$_3$: C, 68.89; H, 9.13; N, 4.23%; Found: C, 68.71; H, 9.05; N, 4.22%.

Figure S22: ^{1}H NMR spectrum of compound **7**.

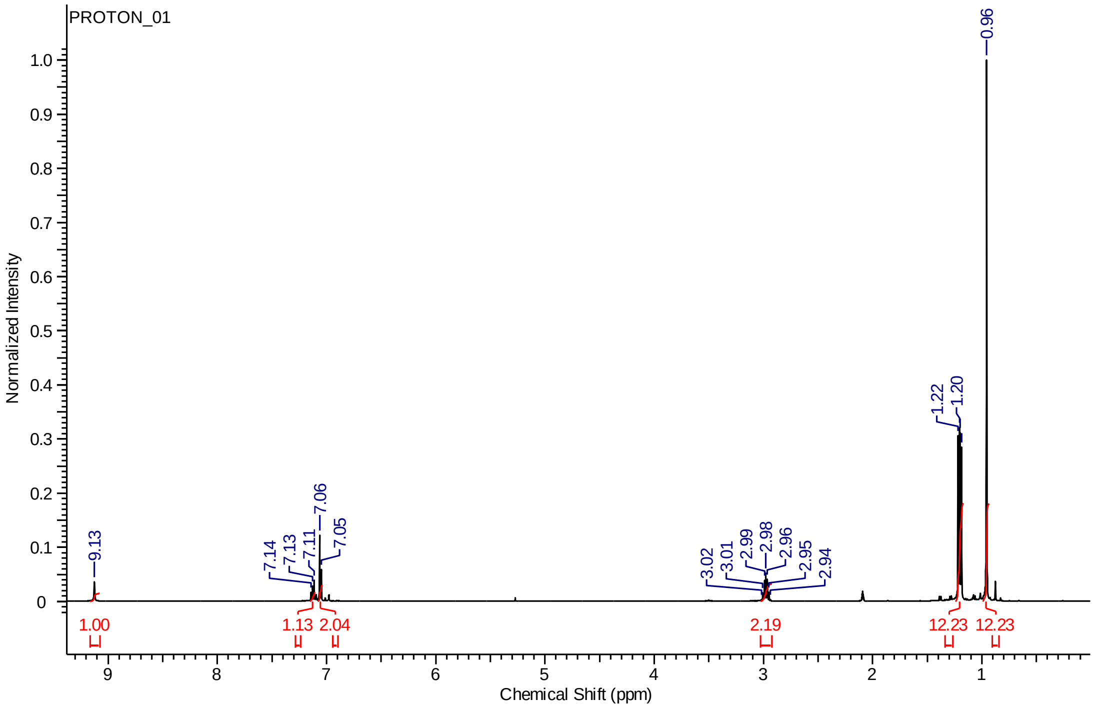

Figure S23: ^{11}B NMR spectrum of compound **7**.

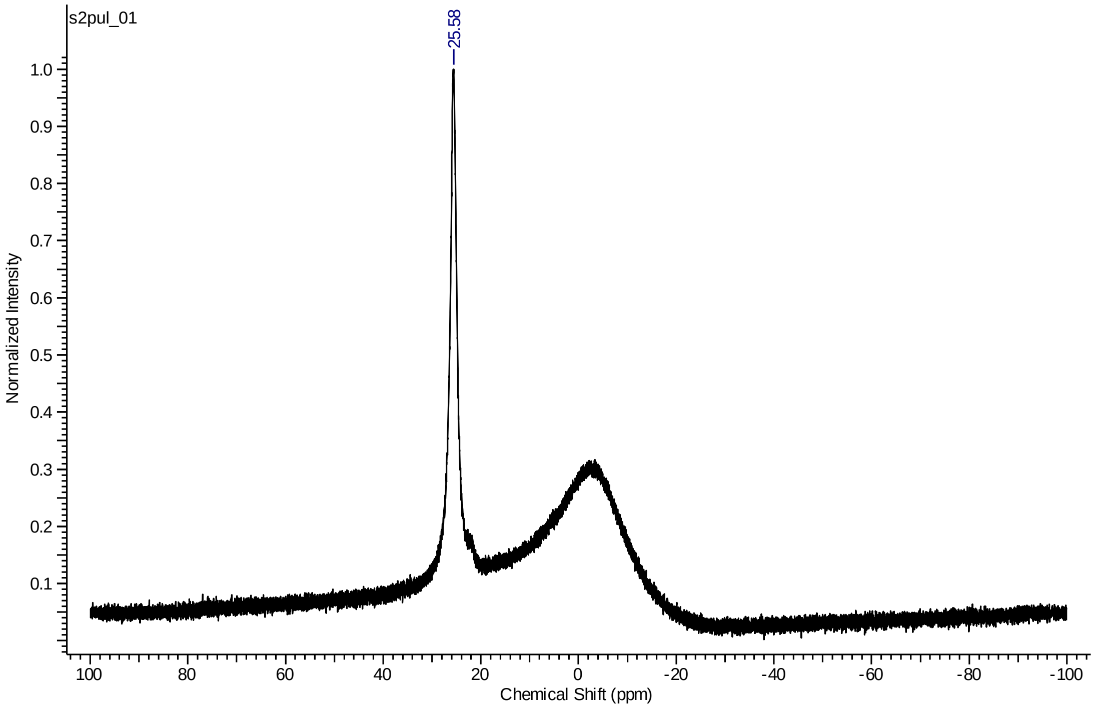

Figure S24: ^{13}C{^{1}H} NMR spectrum of compound **7**.

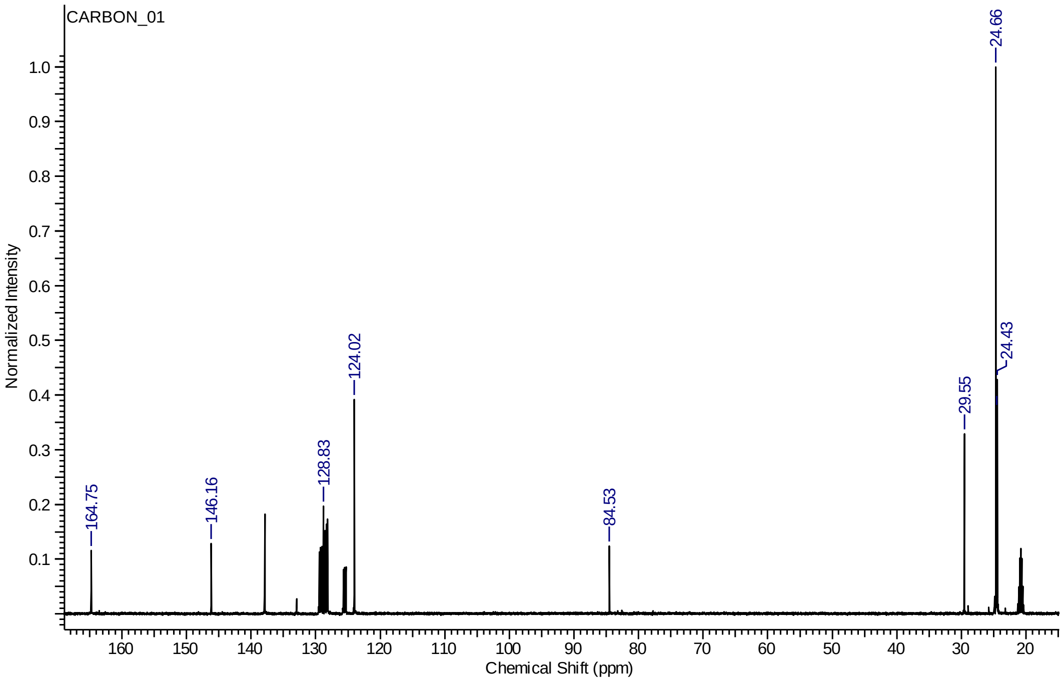

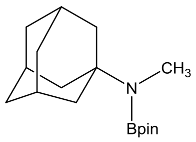

Hydroboration of 1-adamantyl isocyanate; N,N-methyl-{B(OCMe$_2$)$_2$}-adamantylamine: 35 mg of adamantyl isocyanate. ^{1}H NMR (500 MHz, C_6D_6, 300 K) δ_H (ppm): 2.73 (3H, s, NCH_3), 1.99 – 1.58 (ca. 15H, m, Ad-H), 1.10 (12H, s, $OC(CH_3)_2$). ^{13}C{^{1}H} NMR (125.8 MHz, C_6D_6, 300 K) δ_C (ppm): 83.5 ($OC(CH_3)_2$), 42.6 (NCH_3), 37.4 (Ad-C), 30.8 (Ad-C), 25.3 ($OC(CH_3)_2$), 25.1 (Ad-C). ^{11}B NMR (160.4 MHz, C_6D_6, 300K) δ_B (ppm): 27.5 (br. s, NB).

Figure 25: ^{1}H NMR spectrum resulting from the hydroboration of AdNCO with HBpin catalysed by 10 mol% **1**.

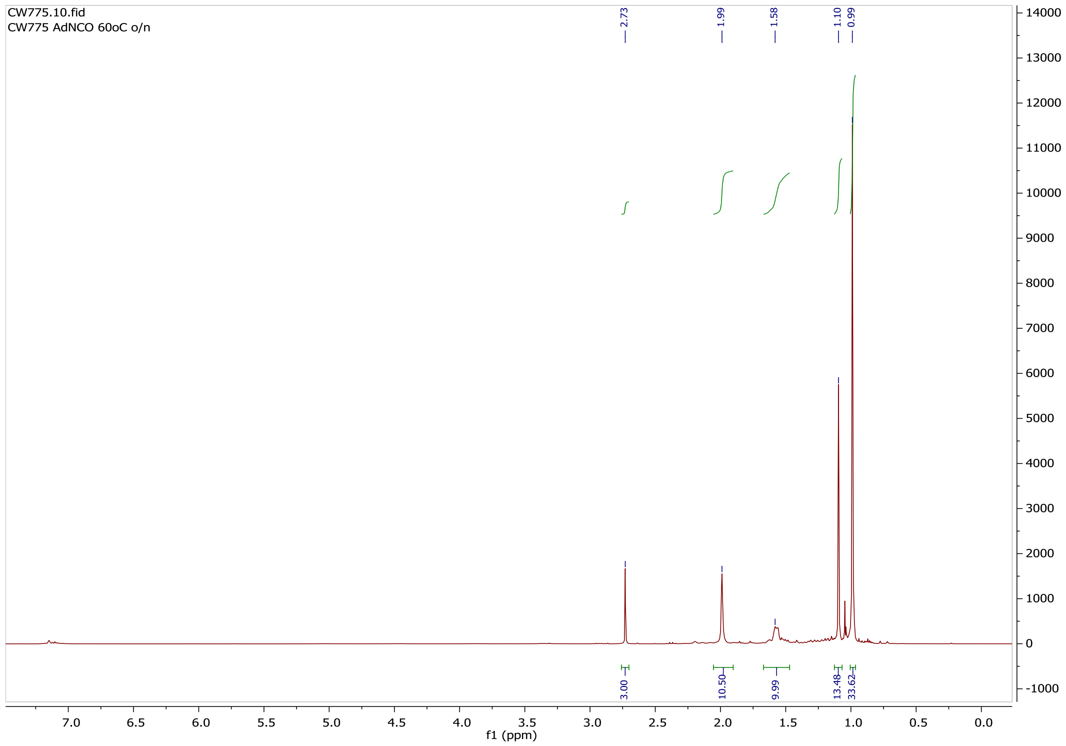

Figure S26: ^{11}B NMR spectrum resulting from the hydroboration of AdNCO with HBpin catalysed by 10 mol% **1**. Resonance observed at δ 37.5 ppm arises from *n*-BuBpin generated by the activation of the pre-catalyst (**1**) and the resonance observed at δ 8.4 ppm is assigned to the *in situ* formation of an intermediate borate species analogous to compound **3**.

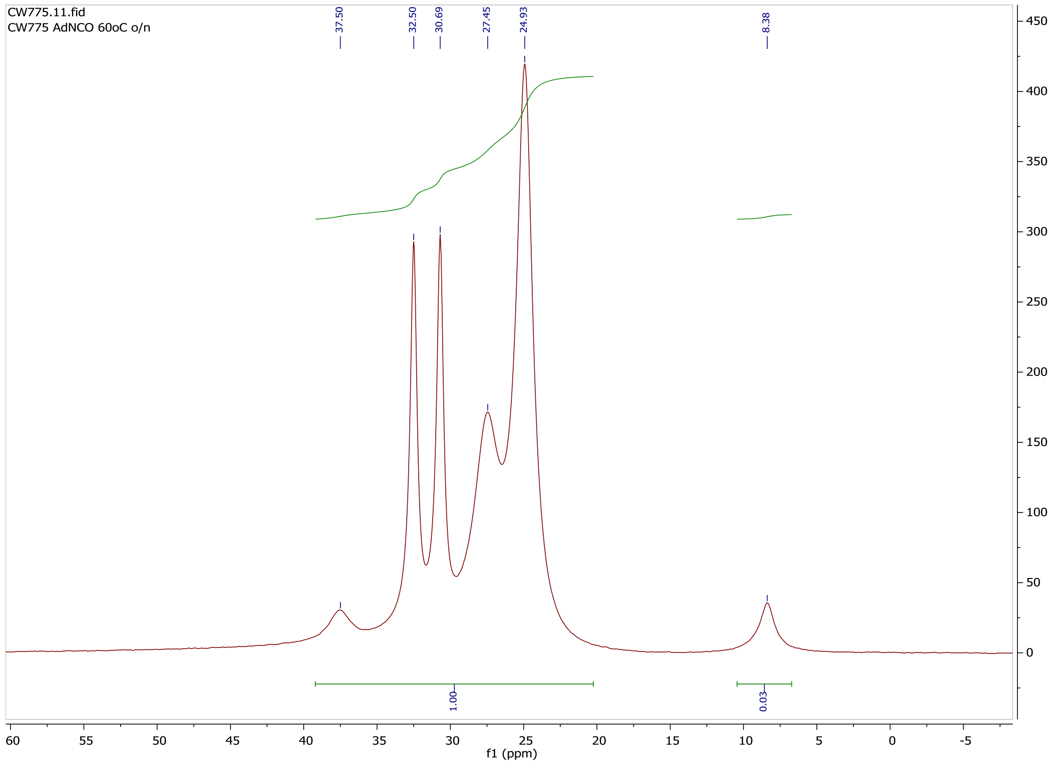

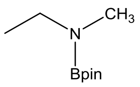

Hydroboration of ethyl isocyanate; N,N-methyl-{B(OCMe$_2$)$_2$}-ethylamine: 15.8 µL of ethyl isocyanate. ^{1}H NMR (500 MHz, C_6D_6, 300 K) δ_H (ppm): 3.00 (2H, q, J_{HH} = 5 Hz, NCH_2), 2.62 (3H, s, NCH_3), 1.11 (12H, s, $OC(CH_3)_2$), 0.98 (3H, t, J_{HH} = 5 Hz, NCH_2CH_3). ^{13}C{^{1}H} NMR (125.8 MHz, C_6D_6, 300 K) δ_C (ppm): 82.3 ($OC(CH_3)_2$), 43.7 (NCH_2), 33.4 (NCH_3), 25.0 ($OC(CH_3)_2$), 15.0 (NCH_2CH_3). ^{11}B NMR (160.4 MHz, C_6D_6, 300K) δ_B (ppm): 27.2 (br. s, NB).

Figure S27: ^{1}H NMR spectrum resulting from the hydroboration of EtNCO with HBpin catalysed by 10 mol% **1**.

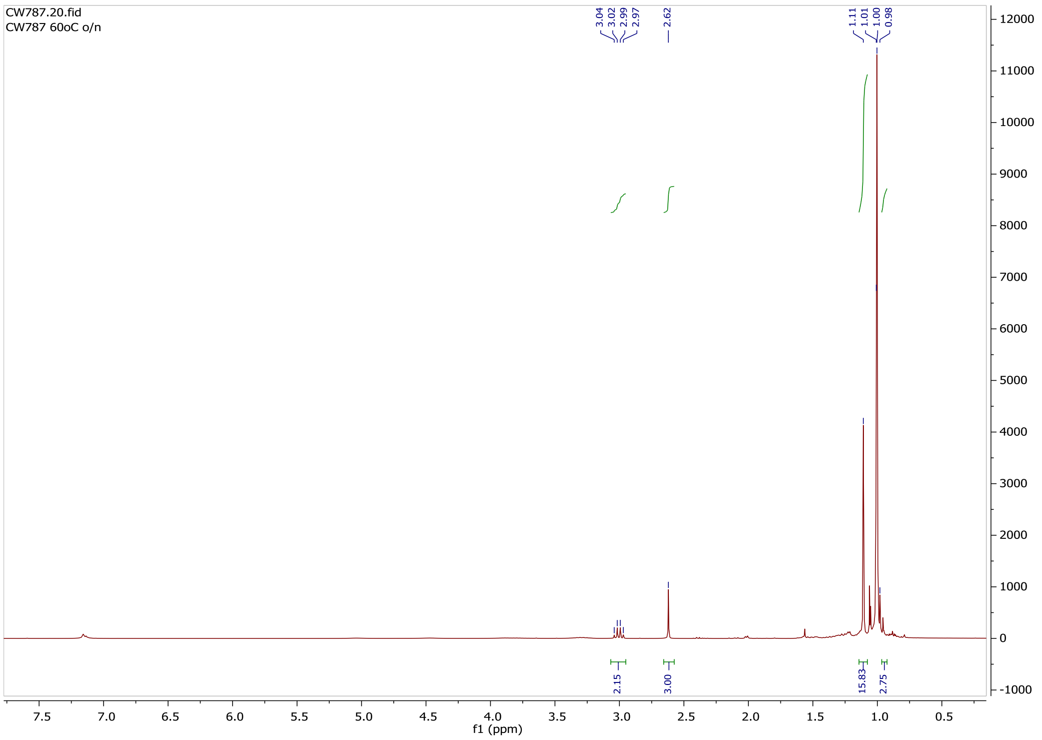

Figure S28: ^{11}B NMR spectrum resulting from the hydroboration of EtNCO with HBpin catalysed by 10 mol% **1**. Resonance observed at δ 37.5 ppm arises from *n*-BuBpin generated by the activation of the pre-catalyst (**1**) and the resonance observed at δ 8.1 ppm is assigned to the *in situ* formation of an intermediate borate species analogous to compound **3**.

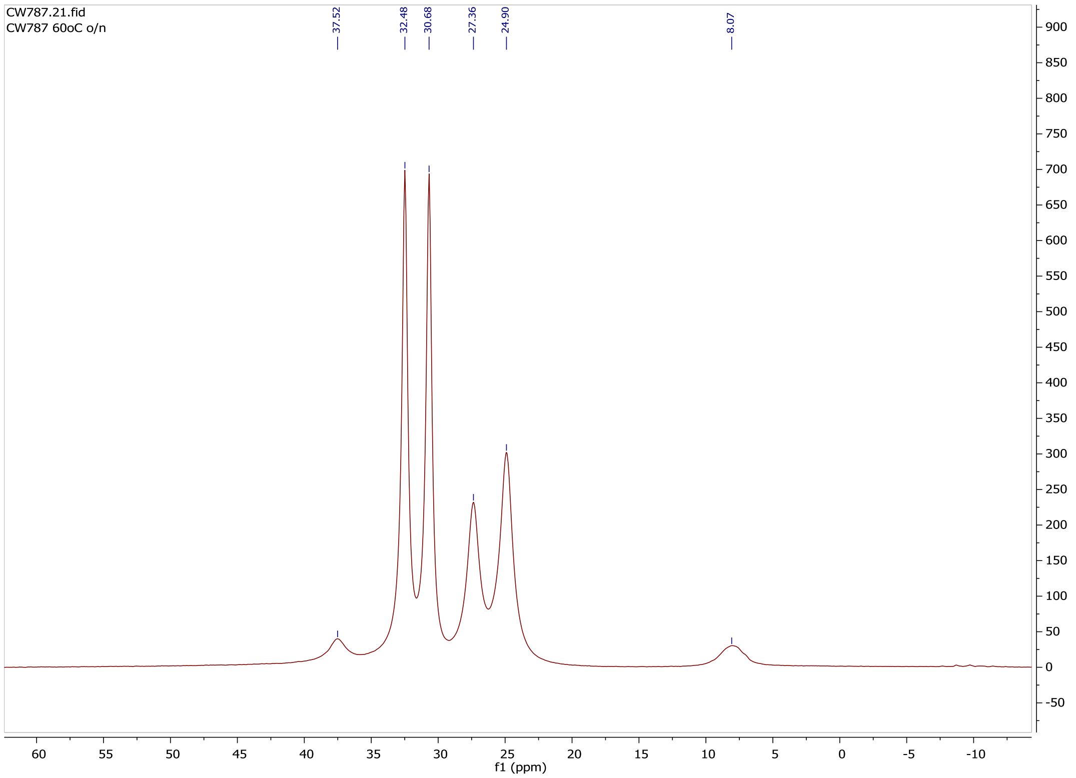

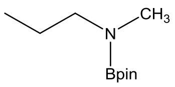

Hydroboration of propyl isocyanate; N,N-methyl-{$B(OCMe_2)_2$}-propylamine: 18.7 µL of propyl isocyanate. ^{1}H NMR (500 MHz, C_6D_6, 300 K) δ_H (ppm): 2.93 (2H, t, J_{HH} = 5 Hz, NCH_2), 2.60 (3H, s, NCH_3), 1.40 (2H, q, J_{HH} = 5 Hz, NCH_2CH_2), 1.11 (12H, s, $OC(CH_3)_2$), 0.81 (3H, t, J_{HH} = 5 Hz, CH_2CH_3). $^{13}C\{^1H\}$ NMR (125.8 MHz, C_6D_6, 300 K) δ_C (ppm): 83.5 ($OC(CH_3)_2$), 50.9 (NCH_2), 33.7 (NCH_3), 25.3 ($OC(CH_3)_2$), 22.0 (NCH_2CH_2), 11.5 (CH_2CH_3). ^{11}B NMR (160.4 MHz, C_6D_6, 300K) δ_B (ppm): 27.4 (br. s, NB).

Figure S29: ^1H NMR spectrum resulting from the hydroboration of *n*-PrNCO with HBpin catalysed by 10 mol% **1**.

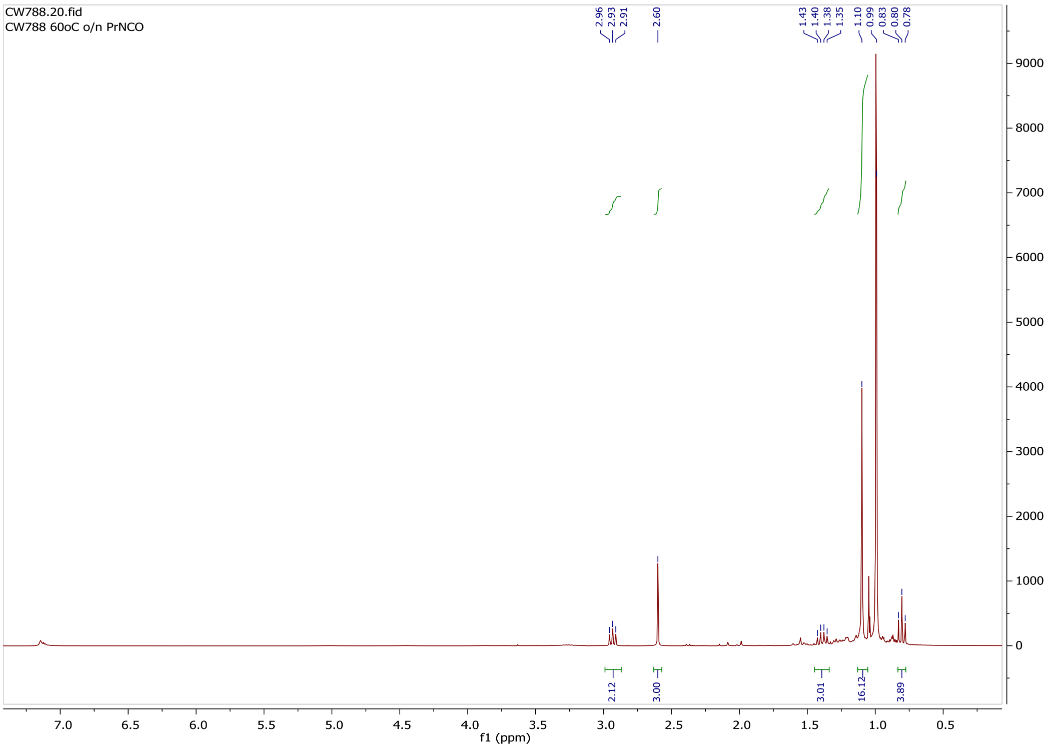

Figure S30: ^{11}B NMR spectrum resulting from the hydroboration of *n*-PrNCO with HBpin catalysed by 10 mol% **1**. Resonance observed at δ 37.5 ppm arises from *n*-BuBpin generated by the activation of the pre-catalyst (**1**) and the resonance observed at δ 7.7 ppm is assigned to the *in situ* formation of an intermediate borate species analogous to compound **3**.

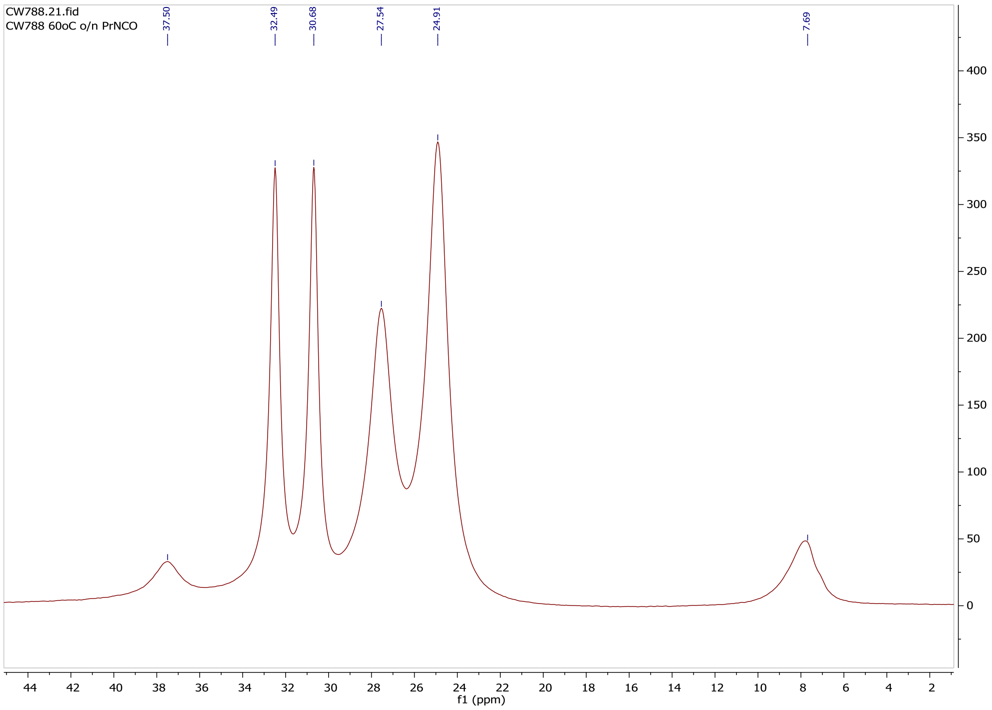

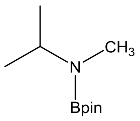

Hydroboration of iso-propyl isocyanate; N,N-methyl-{B(OCMe$_2$)$_2$}-iso-propylamine: 19.7 µL of *iso*-propyl isocyanate. ^{1}H NMR (500 MHz, C_6D_6, 300 K) δ_H (ppm): 3.79 (1H, sept, J_{HH} = 5 Hz, CH(CH$_3$)$_2$), 2.56 (3H, s, NCH$_3$), 1.11 (12H, s, OC(CH$_3$)$_2$), 1.03 (6H, d, J_{HH} = 10 Hz, CH(CH$_3$)$_2$). ^{13}C{^{1}H} NMR (125.8 MHz, C_6D_6, 300 K) δ_C (ppm): 83.2 (OC(CH$_3$)$_2$), 46.8 (NCH$_3$), 27.5 (NCH(CH$_3$)$_2$), 25.2 (OC(CH$_3$)$_2$), 21.4 (CH(CH$_3$)$_2$). ^{11}B NMR (160.4 MHz, C_6D_6, 300K) δ_B (ppm): 27.2 (br. s, NB).

Figure S31: ^{1}H NMR spectrum resulting from the hydroboration of *i*-PrNCO with HBpin catalysed by 10 mol% **1**.

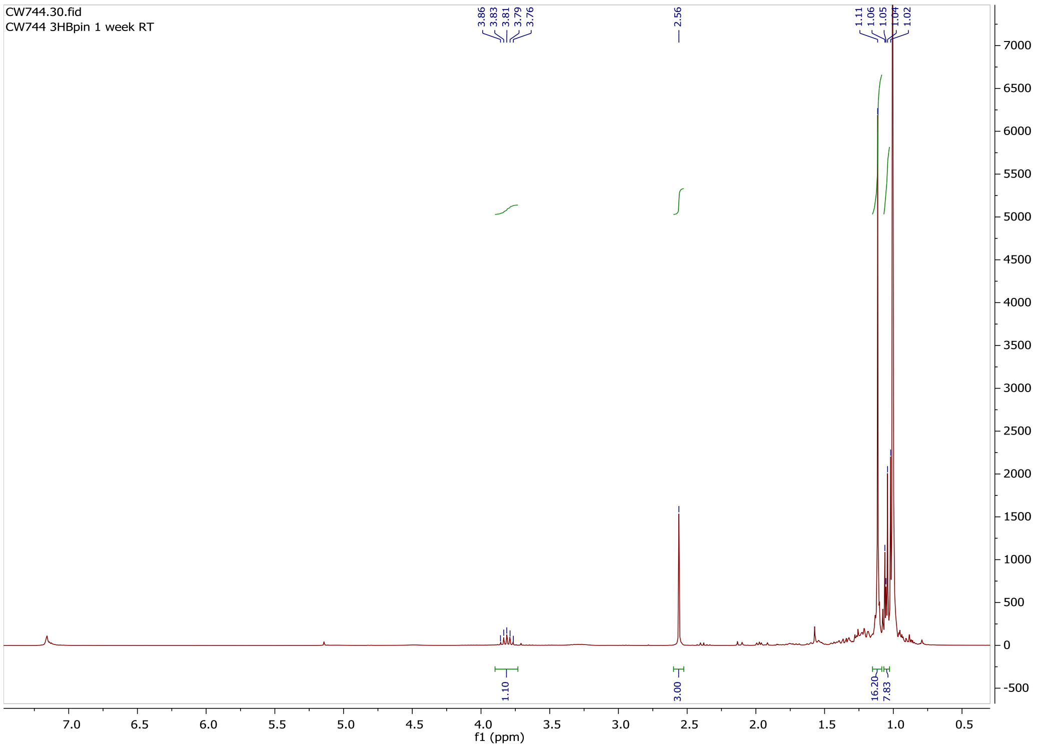

Figure S32: ^{11}B NMR spectrum resulting from the hydroboration of *i*-PrNCO with HBpin catalysed by 10 mol% **1**. Resonance observed at δ 37.5 ppm arises from *n*-BuBpin generated by the activation of the pre-catalyst (**1**) and the resonances observed at δ 7.8 and 7.1 ppm are assigned to the *in situ* formation of intermediate borate species.

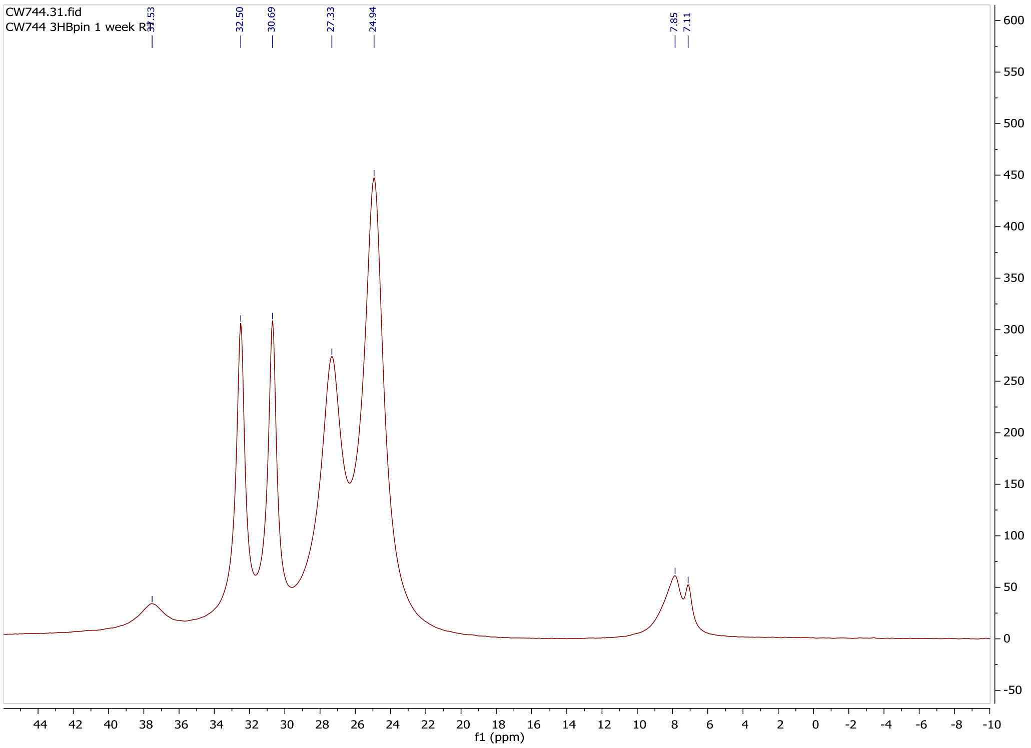

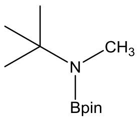

Hydroboration of tert-butyl isocyanate; N,N-methyl-{B(OCMe$_2$)$_2$}-tert-butylamine: 22.8 µL of *tert*-butyl isocyanate. ^{1}H NMR (500 MHz, C_6D_6, 300 K) δ_H (ppm): 2.69 (3H, s, NCH_3), 1.28 (9H, s, $C(CH_3)_3$), 1.09 (12H, s, $OC(CH_3)_2$). ^{13}C{^{1}H} NMR (125.8 MHz, C_6D_6, 300 K) δ_C (ppm): 83.5 ($OC(CH_3)_2$), 52. ($C(CH_3)_3$), 30.2 ($C(CH_3)_3$), 25.1 ($OC(CH_3)_2$). ^{11}B NMR (160.4 MHz, C_6D_6, 300K) δ_B (ppm): 24.3 (br. s, NB).

Figure S33: ^{1}H NMR spectrum resulting from the hydroboration of *t*-BuNCO with HBpin catalysed by 10 mol% **1**.

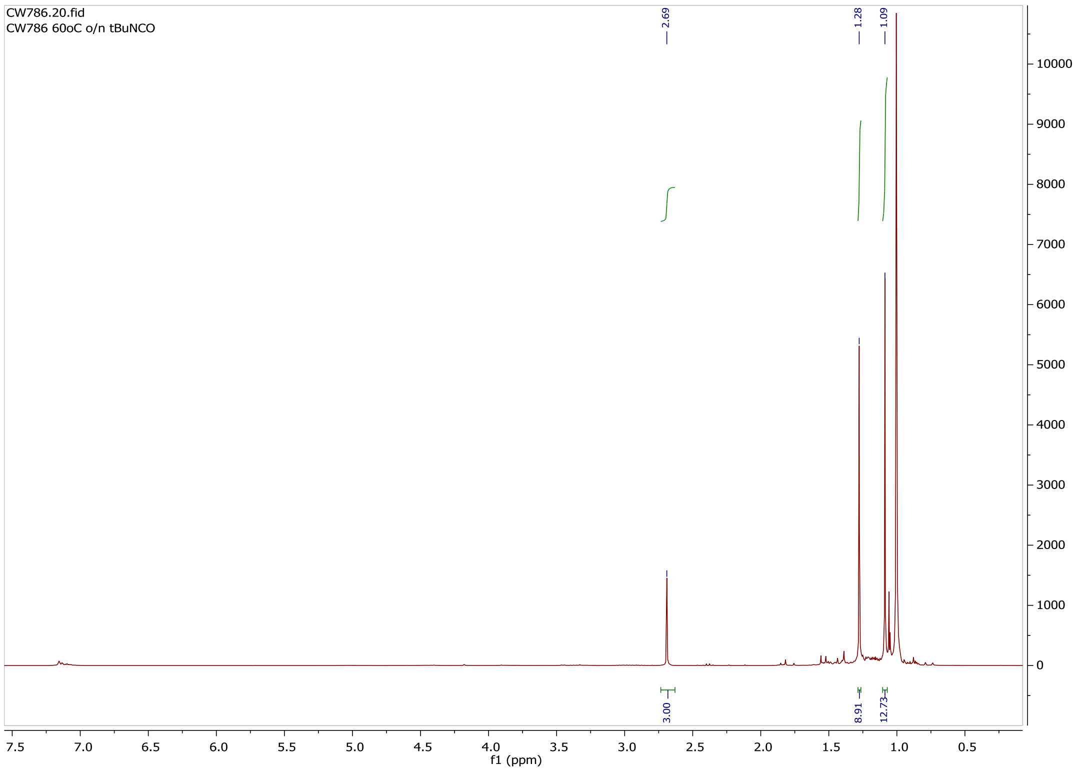

Figure S34: ^{11}B NMR spectrum resulting from the hydroboration of *t*-BuNCO with HBpin catalysed by 10 mol% **1**. Resonance observed at δ 37.5 ppm arises from *n*-BuBpin generated by the activation of the pre-catalyst (**1**) and the resonance observed at δ 8.3 ppm is assigned to the *in situ* formation of an intermediate borate species.

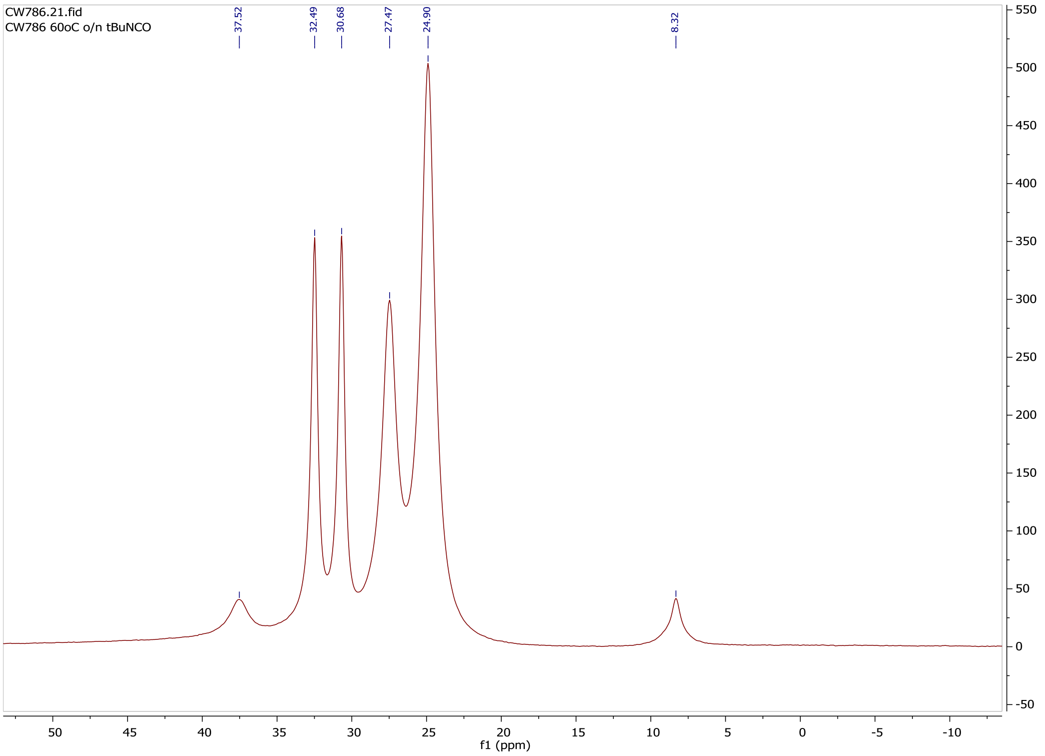

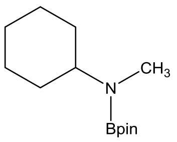

Hydroboration of cyclohexyl isocyanate; N,N-methyl-{B(OCMe$_2$)$_2$}-cyclohexylamine: 25.5 µL of cyclohexyl isocyanate. ^{1}H NMR (500 MHz, C_6D_6, 300 K) δ_H (ppm): 3.29 (1H, m, NCH), 2.63 (3H, s, NCH_3), 1.62 (4H, m, NCH(CH_2)$_2$), 1.45 (4H, m, NCH(CH_2)$_2$(CH_2)$_2$), 1.12 (12H, s, OC(CH_3)$_2$), 0.88 (2H, m, (CH_2)$_2$$CH_2$). ^{13}C{^{1}H} NMR (125.8 MHz, C_6D_6, 300 K) δ_C (ppm): 83.5 (OC(CH_3)$_2$), 55.7 (NCH), 32.3 (NCH_3), 29.1 (NCH(CH_2)$_2$), 26.8 (NCH(CH_2)$_2$(CH_2)$_2$), 25.9 (OC(CH_3)$_2$), 14.7 ((CH_2)$_2$$CH_2$). ^{11}B NMR (160.4 MHz, C_6D_6, 300K) δ_B (ppm): 27.4 (br. s, NB).

Figure S35: ^{1}H NMR spectrum resulting from the hydroboration of *Cy*NCO with HBpin catalysed by 10 mol% **1**.

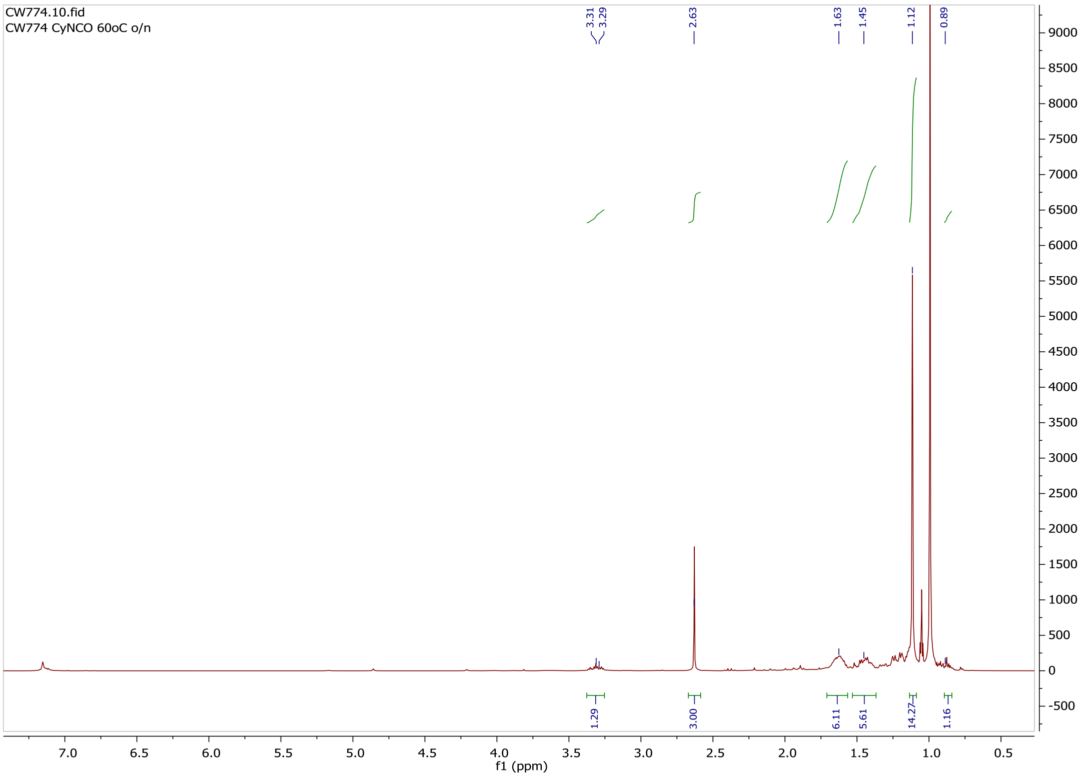

Figure S36: ^{11}B NMR spectrum resulting from the hydroboration of *t*-BuNCO with HBpin catalysed by 10 mol% **1**. Resonance observed at δ 37.5 ppm arises from *n*-BuBpin generated by the activation of the pre-catalyst (**1**) and the resonances observed at δ 7.8 and7.3 ppm are assigned to the *in situ* formation of intermediate borate species.

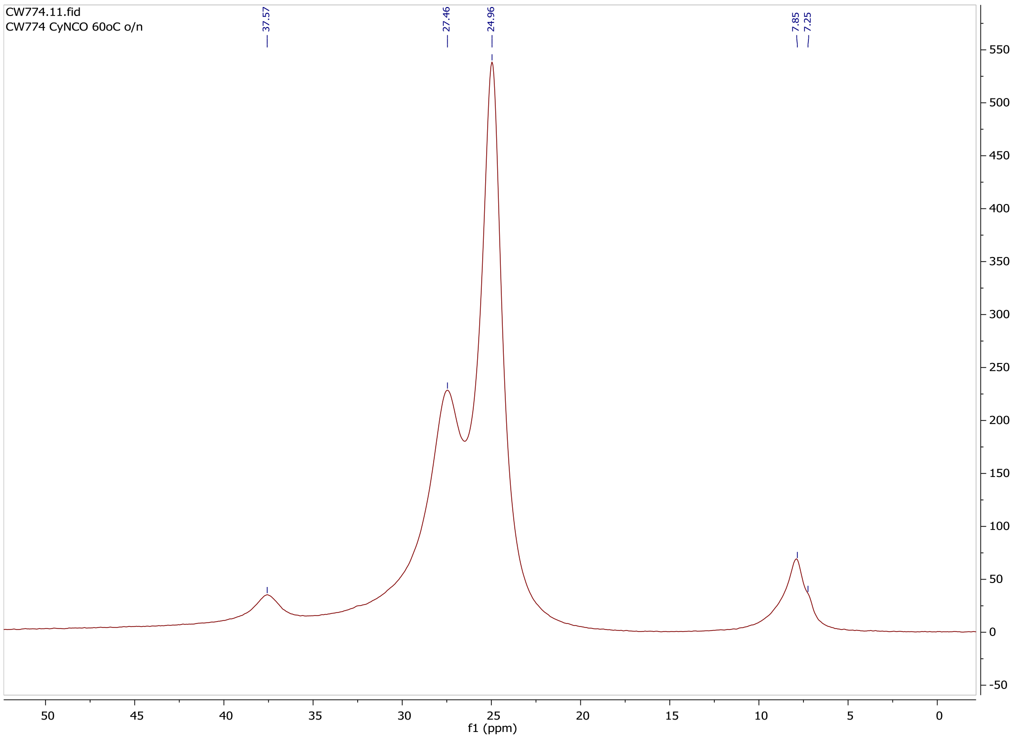

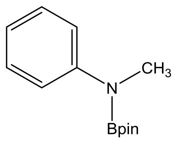

Hydroboration of phenyl isocyanate; N,N-methyl-{B(OCMe$_2$)$_2$}-phenylamine: 21.7 μL of phenyl isocyanate. ^{1}H NMR (500 MHz, C_6D_6, 300 K) δ_H (ppm): 7.45 (2H, m, o-H), 7.21 (1H, m, p-H), 6.87 (2H, m, m-H), 3.01 (3H, s, NCH_3), 1.08 (12H, s, $OC(CH_3)_2$). ^{13}C{^{1}H} NMR (125.8 MHz, C_6D_6, 300 K) δ_C (ppm): 164.2 (ipso-C), 148.3 (o-C), 129.2 (p-C), 121.4 (m-C), 83.5 ($OC(CH_3)_2$), 34.7 (NCH_3), 25.3 ($OC(CH_3)_2$). ^{11}B NMR (160.4 MHz, C_6D_6, 300K) δ_B (ppm): 27.7 (br. s, NB).

Figure S37: ^{1}H NMR spectrum resulting from the hydroboration of PhNCO with HBpin catalysed by 10 mol% **1**.

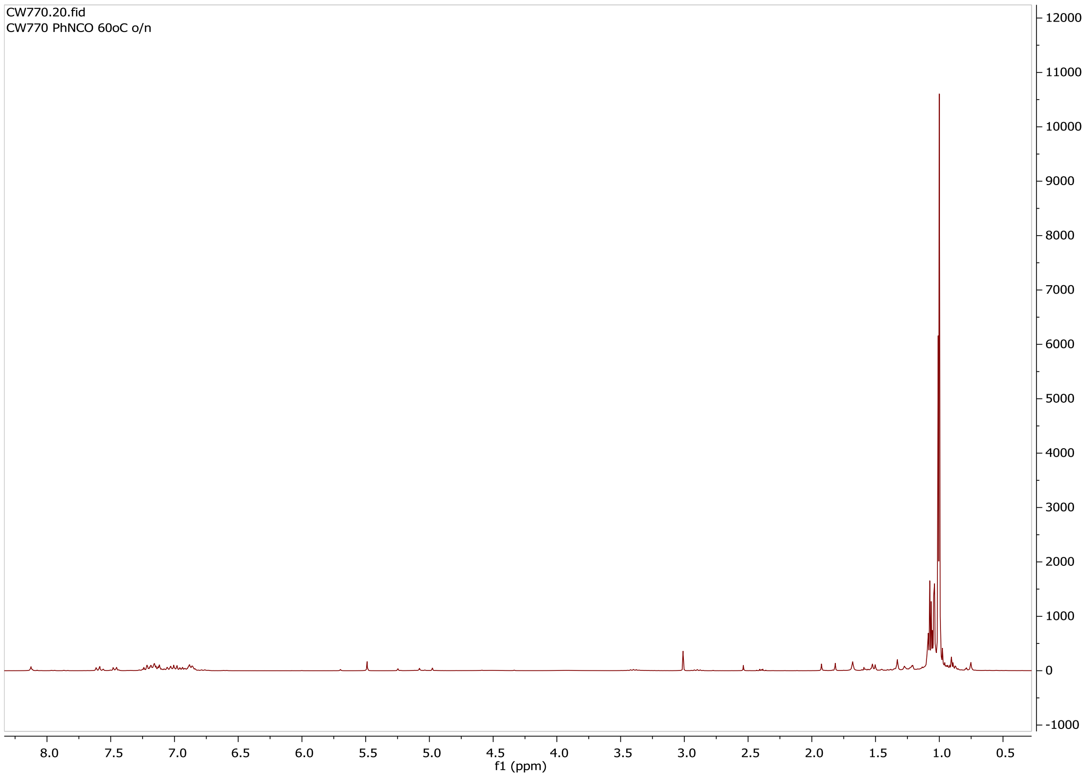

Figure S38: ^{11}B NMR spectrum resulting from the hydroboration of PhNCO with HBpin catalysed by 10 mol% **1**. Resonance observed at δ 37.5 ppm arises from *n*-BuBpin generated by the activation of the pre-catalyst (**1**) and the resonance observed at δ 8.2 ppm is assigned to the *in situ* formation of an intermediate borate species.

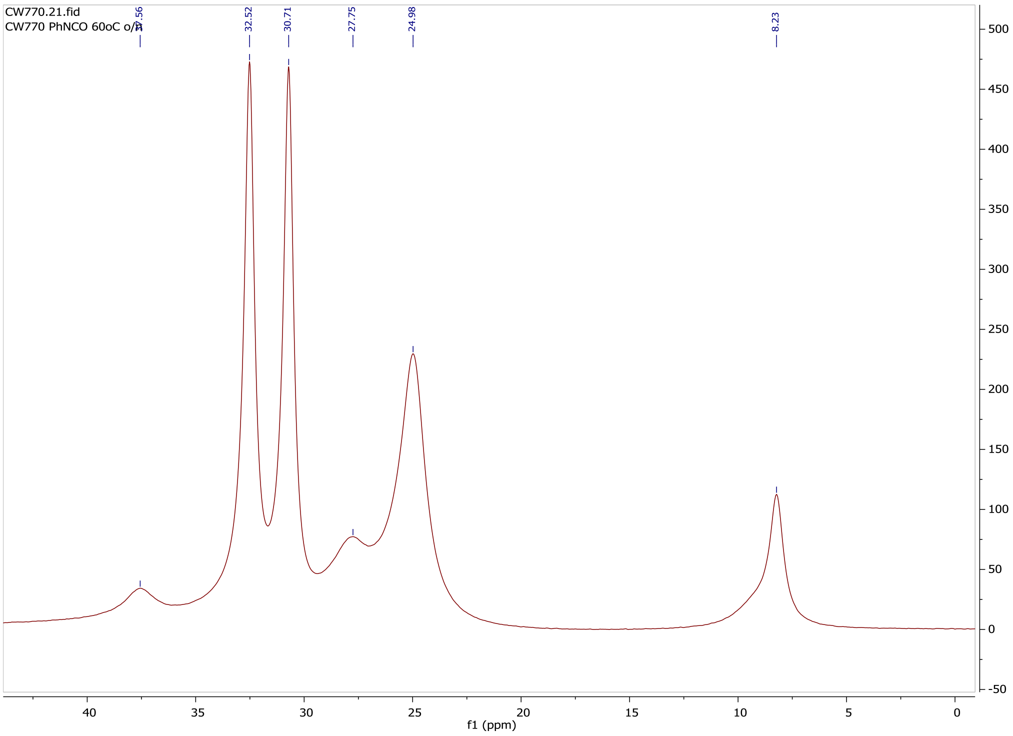

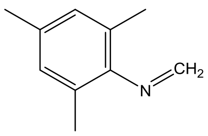

Formation of N-2,4,6-trimethylphenyl imine: A sample of compound **6** in C_6D_6 was heated for 12 hours at 60 °C, providing ca. 50% conversion to MesN=CH_2. ^{1}H NMR (500 MHz, C_6D_6, 300 K) δ_H (ppm): 7.24 (1H, d, J_{HH} = 12 Hz, NC*H*$_2$), 6.82 (1H, d, J_{HH} = 12 Hz, NC*H*$_2$), 6.76 (2H, s, *m*-*H*), 2.25 (6H, s, *o*-C*H*$_3$), 2.09 (3H, s, *p*-C*H*$_3$). ^{13}C{^{1}H} NMR (75.5 MHz, C_6D_6, 300 K) δ_C (ppm): 138.2 (*ipso*-*C*), 135.2 (*o*-*C*), 133.4 (*p*-*C*), 129.4 (NC*H*$_2$), 124.7 (*m*-*C*), 23.4 (*o*-*C*H$_3$), 18.6 (*p*-*C*H$_3$).]

Figure S39: ^{1}H NMR spectrum of a sample of compound **6** in C_6D_6 after heating at 60 °C for 12 hours showing the production of ca. 50 % MesN=CH_2.

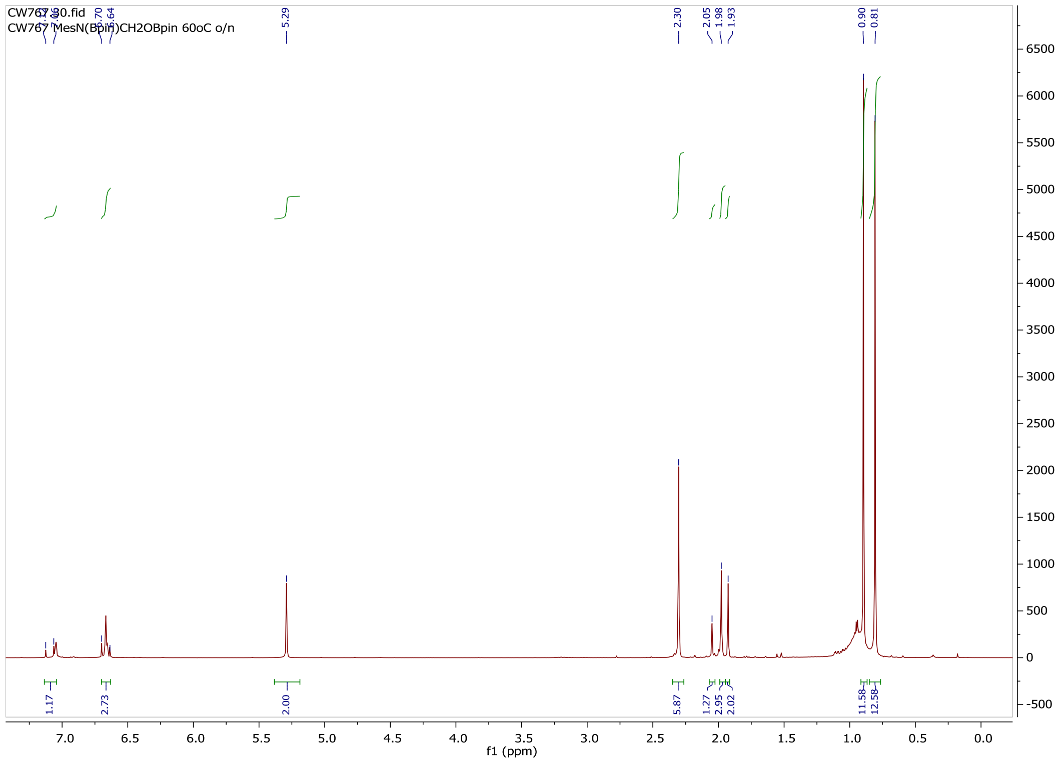

Figure S40: ^{1}H NMR spectrum of a sample of compound **6** in C_6D_6 after heating at 60 °C for 12 hours showing the complete conversion to MesN=CH_2.

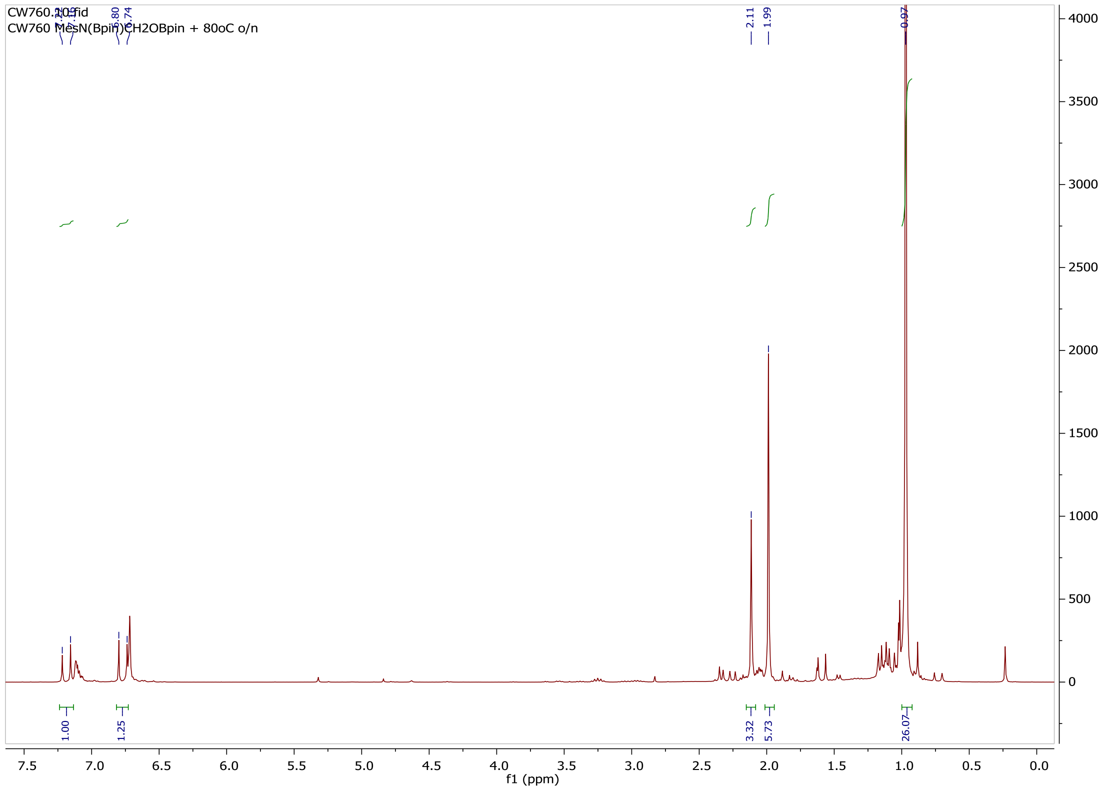

Figure S41: ^{1}H NMR spectrum (300 MHz) resulting from the room temperature reaction of compound **6** and [HC{(Me)CN(2,6-^{i}Pr$_2$C$_6$H$_3$)}$_2$MgH]$_2$ at room temperature demonstrating the facile production of MesN(Me)Bpin and compound **4** (amine resonances labelled).

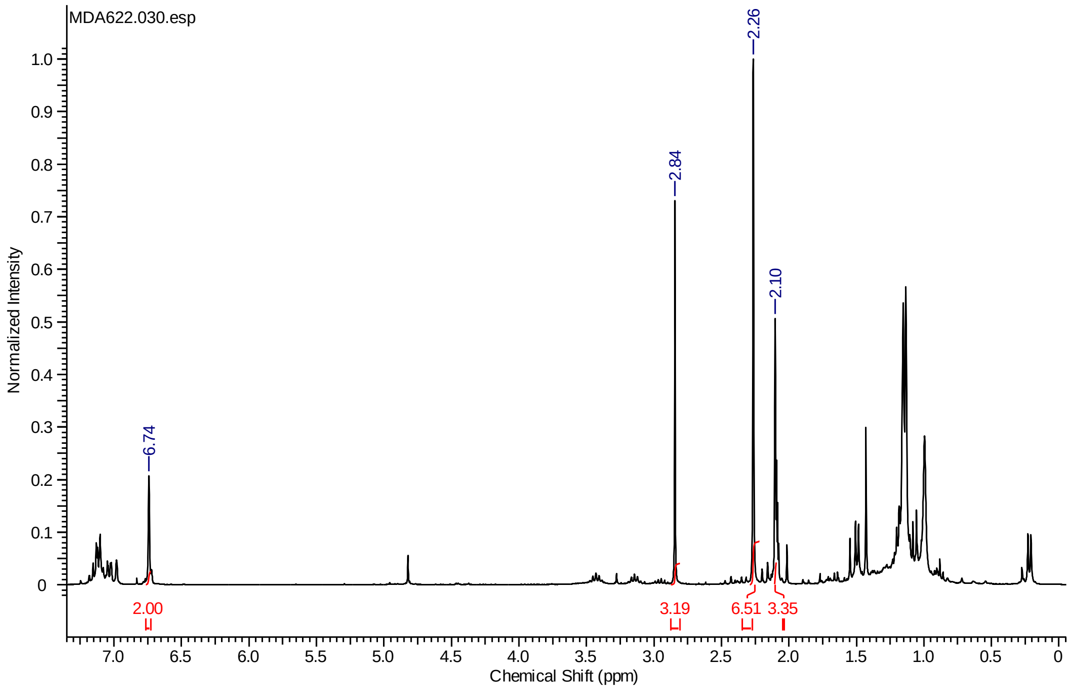

Figure S42: ^{1}H NMR spectrum (300 MHz) resulting from the room temperature reaction of compound **6** and [HC{(Me)CN(2,6-^{i}Pr$_2$C$_6$H$_3$)}$_2$MgH]$_2$ at room temperature demonstrating the facile production of MesN(Me)Bpin and compound **4** (compound **4** resonances labelled).

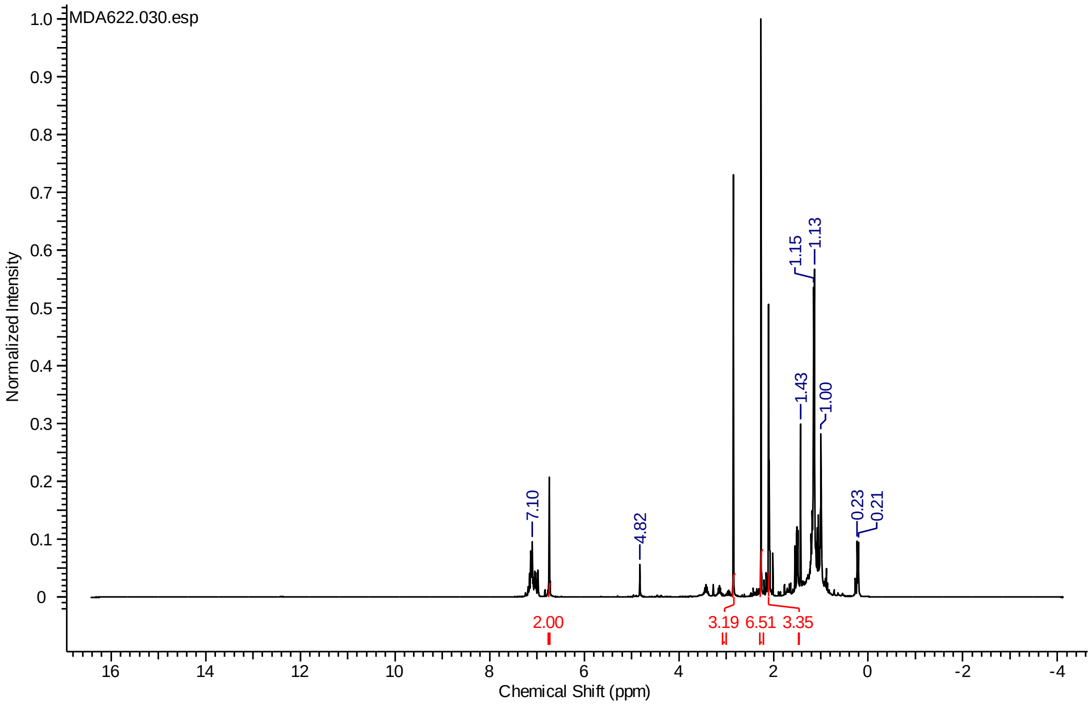

Figure S43: ^{11}B NMR spectrum (96.3 MHz) resulting from the room temperature reaction of compound **6** and [HC{(Me)CN(2,6-^{i}Pr$_2$C$_6$H$_3$)}$_2$MgH]$_2$ at room temperature demonstrating the facile production of compound **4**.

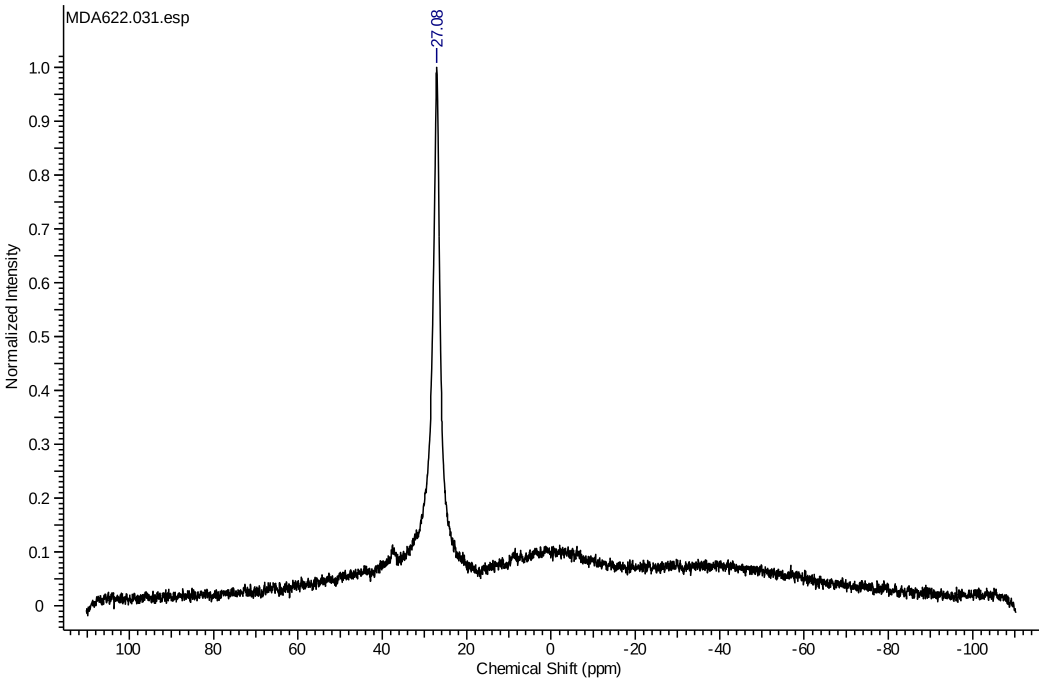

Figure S44: ^{13}C{^{1}H} NMR spectrum (75.5 MHz) resulting from the room temperature reaction of compound **6** and [HC{(Me)CN(2,6-^{i}Pr$_2$C$_6$H$_3$)}$_2$MgH]$_2$ at room temperature demonstrating the facile production of MesN(Me)Bpin and compound **4** (amine resonances labelled).

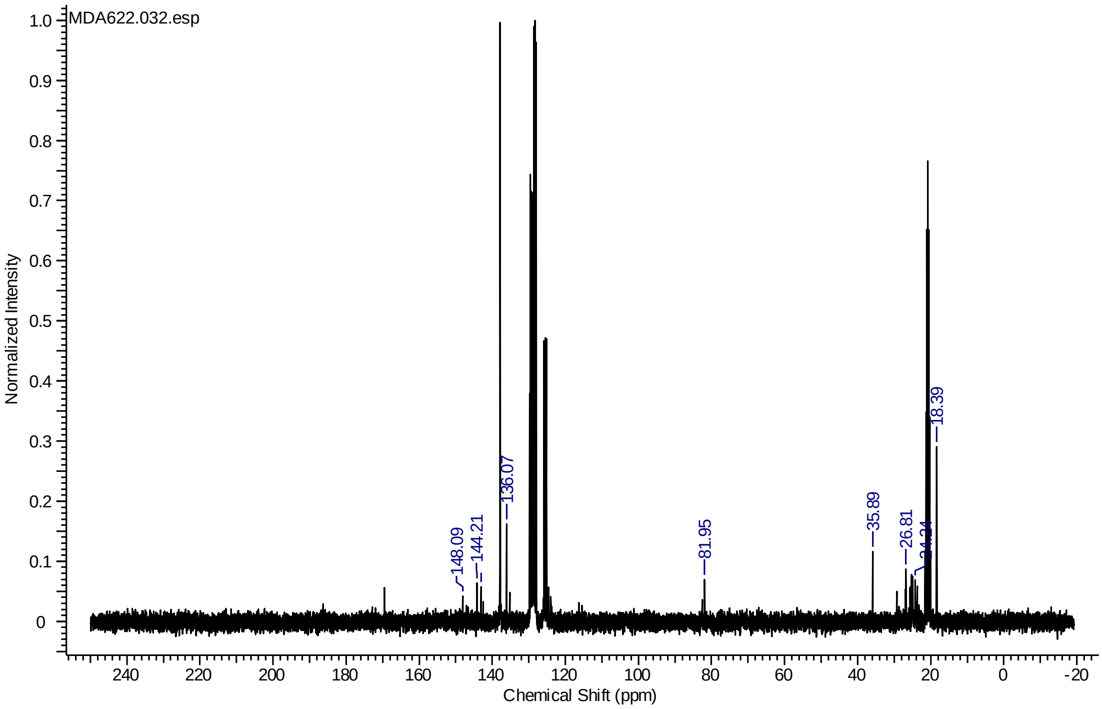

Figure S45: ^{1}H NMR spectrum (400 MHz) for the reaction of HBpin and i-PrNCO catalysed by 10 mol% 1 at 60 °C after 180 minutes.

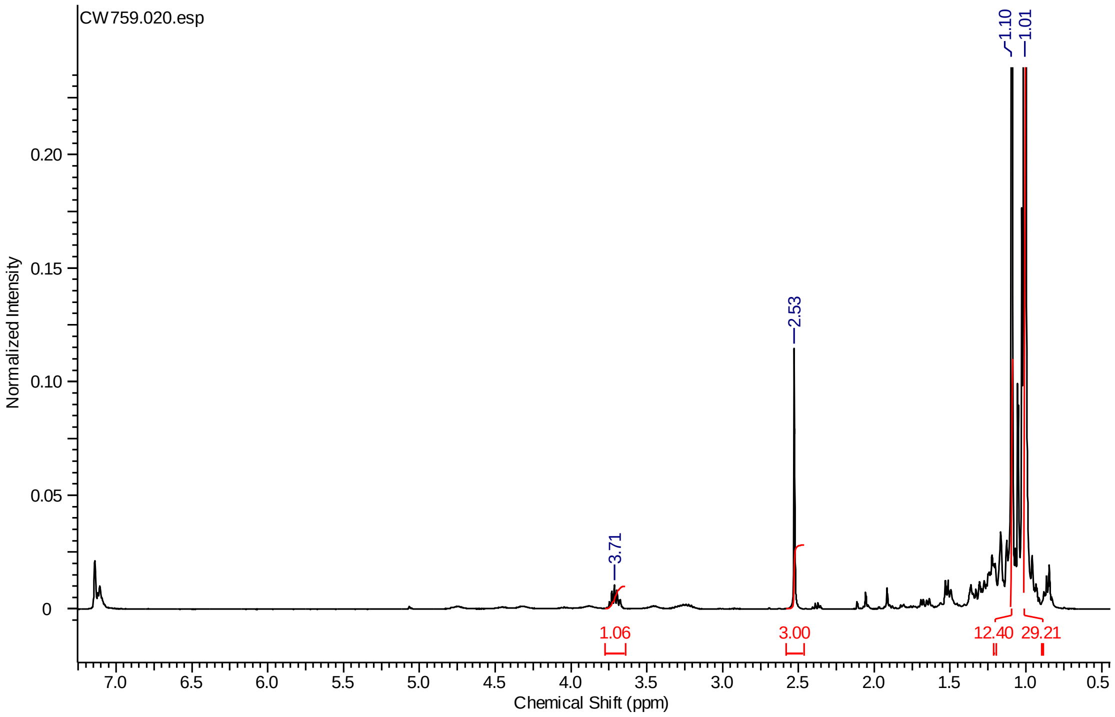

Single Crystal X-ray Diffraction analysis: Single Crystal X-ray diffraction data for compound **6** were collected on a Nonius Kappa CCD with a low temperature device at 150 K, utilizing Mo-Kα radiation monochromated with graphite (λ = 0.71070 Å) while those for **5** were attained on an Agilent Xcalibur machine. Processing utilized the Nonius software,[3] with structure solution and refinement using Olex2,[4] suite of programmes throughout and visualised utilizing ORTEP 3.[5] For compounds **2** – **4** and **7** data were collected on a SuperNova, Dual Cu at zero, EosS2 diffractometer. The crystals were kept at 150(2) K during data collections. Using Olex2,[4] the structures were solved *via* SHELXS[6] and refined with the ShelXL refinement package using Least Squares minimisation. The asymmetric unit of compound **3** incorporated one molecule of the magnesium complex and one molecule of toluene. C25 and C26 were modelled as being disordered over 2 sites in a 65:35 ratio. Distances from the arising fractional occupancy carbon atoms were refined subject to being similar and ADP restraints were included for C26A. H1, attached to B1, was readily located and refined freely. Disorder also prevailed in the solvent region such that the toluene entity has been modelled as being distributed over 3 overlaid sites in a 50:25:25 ratio. The associated phenyl rings were treated as rigid hexagons - and planarity, distance and ADP restraints were employed (on merit) to achieve the optimal chemically sensible refinement. The asymmetric unit of compound **4** incorporated half of a dimer molecule and a half of a molecule of toluene. Both moieties lie proximate to crystallographic inversion centres. All atoms in the Bpin ligand, with the exception of the B1, were seen to be disordered over 2 proximate locations in a 55:45 ratio. Distance and ADP restraints were employed in this region to assist convergence. It was very evident that the solvent equates to half of one molecule of toluene per asymmetric unit, and allowance for this has been made in the formula as presented herein. However, proximity to an inversion centre plus disorder did not facilitate a ready treatment with a model and, hence, the PLATON SQUEEZE algorithm was employed in this instance. The carbon atoms in both Bpin anions of compound **5** exhibited 65:35 disorder, which was successfully modelled, subject to distance restraints being applied to chemically similar bonds involving fractional occupancy carbons. Compound **6** contained 2 molecules per asymmetric unit.

CCDC 1504016-1504019 contain the supplementary crystallographic data for compounds **2**, **3**, **4** and **6**, respectively, while the data for compound **5** and **7** have respective codes of CCDC 1526366-1526367. These data can be obtained free of charge from The Cambridge Crystallographic Data Center via www.ccdc.ca.ac.uk/data_request/cif.

Table S1: Single Crystal X-ray Data Parameters for compounds **2** – **7**.

Compound	**2**	**3**	**4**	**5**	**6**	**7**
Empirical formula	$C_{42}H_{59}MgN_3O$	$C_{55}H_{80}BMgN_3O_3$	$C_{38.5}H_{57}BMgN_2O_3$	$C_{25}H_{43}B_2NO_5$	$C_{22}H_{37}B_2NO_5$	$C_{25}H_{43}B_2NO_5$
Formula weight	646.23	866.34	630.98	459.22	417.14	459.22
Temperature/K	150.01(10)	150.01(10)	150.00(10)	150.01(10)	150(2)	150.00(10)
Crystal system	monoclinic	monoclinic	monoclinic	orthorhombic	monoclinic	trigonal
Space group	$P2_1/n$	$P2_1/c$	$P2_1/n$	$P2_12_12_1$	$P2_1/n$	$P3_1$
a/Å	11.59209(14)	9.17488(14)	13.48093(17)	10.0273(4)	11.5490(2)	9.7577(2)
b/Å	26.2784(3)	19.0562(3)	14.64625(15)	12.4499(7)	33.1610(6)	9.7577(2)
c/Å	12.80407(16)	30.0078(6)	18.8268(2)	21.5324(9)	13.3198(2)	17.8748(4)
α/°	90	90	90	90	90	90
β/°	91.4753(11)	93.2810(17)	94.1686(12)	90	105.633(1)	90
γ/°	90	90	90	90	90	120
Volume/Å^3	3899.10(8)	5237.92(16)	3707.42(8)	2688.1(2)	4912.46(15)	1473.89(7)
Z	4	4	4	4	8	3
ρ_{calc} g/cm^3	1.101	1.099	1.130	1.135	1.128	1.552
μ/mm^{-1}	0.641	0.617	0.691	0.076	0.077	0.825
F(000)	1408.0	1888.0	1372.0	1000.0	1808.0	750.0
Crystal size/mm^3	0.133×0.091×0.076	0.178×0.046×0.046	0.235×0.119×0.053	0.505×0.133×0.116	0.5×0.4×0.3	0.393×0.129×0.087
2Θ range for data collection/°	6.728 to 139.614	5.496 to 139.68	7.656 to 139.576	6.552 to 54.964	7.346 to 54.798	10.468 to 147.178
Reflections collected	23319	31806	22162	22144	59286	11789
Independent reflections	7278 [R_{int}= 0.0259]	9773 [R_{int} = 0.0362]	6916 [R_{int}= 0.0297]	6057 [R_{int} = 0.0521]	10990[R_{int}= 0.0910]	3061 [R_{int}= 0.0334]
Data/restraints/parameters	7278/0/438	9773/98/703	6916/79/469	6057/154/426	10990/0/564	3061/1/225
Goodness-of-fit on F^2	1.017	1.017	1.024	1.049	1.011	1.049
Final R indexes [I>=2σ (I)]	R_1 = 0.0367, wR_2 = 0.0894	R_1 = 0.0472, wR_2 = 0.1163	R_1 = 0.0482, wR_2 = 0.1230	R_1 = 0.0529, wR_2 = 0.0897	R_1= 0.0498, wR_2= 0.1237	R_1 = 0.0413, wR_2 = 0.1037
Final R indexes [all data]	R_1 = 0.0473, wR_2 = 0.0952	R$_1$ = 0.0623, wR_2 = 0.1262	R_1 = 0.0607, wR_2 = 0.1306	R_1 = 0.0879, wR_2 = 0.1010	R_1= 0.0775, wR_2= 0.1390	R_1 = 0.0433, wR_2 = 0.1053
Largest diff. peak/hole / e Å^{-3}	0.26/-0.24	0.56/-0.48	0.44/-0.35	0.17/-0.15	0.33/-0.23	0.29/-0.16

Computational details

Geometry optimizations were performed using Gaussian09 suite of programs[7] using the Becke's 3-parameter hybrid functional,[8] combined with the non-local correlation functional provided by Perdew/Wang.[9] The 6-311+G(d) all-electron basis set was used for the magnesium and the 6-31G(d,p) for the remaining atoms.[10] All stationary points have been identified for minimum (Nimag=0) or transition states (Nimag=1). Intrinsic Reaction Paths (IRPs)[11] were traced from the various transition structures to obtain the connected intermediates. We have also considered in the present study dispersion effects, in particular the third generation of Grimme's dispersion corrections with Becke-Johnson damping model[12] on the B3PW91 geometries (single point calculations).

Cartesian coordinates of all optimized structures

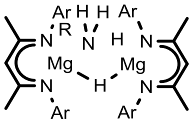

IRC-reverse

N	17.10291000	0.92765200	7.47552600
N	16.23514600	-1.95354000	7.74001500
C	17.45732100	-0.90581300	5.92341800
H	17.91614700	-1.13170900	4.96753500
C	17.59856100	0.43339400	6.34566700
C	16.86520200	-2.01072000	6.57129200
C	18.39200600	1.33611200	5.42382000
H	18.70968100	0.80722100	4.52416000
H	19.28767200	1.71971300	5.92635400
H	17.80201100	2.20790000	5.11833900
C	16.99460700	-3.34148500	5.85913500
H	17.48872200	-3.22999100	4.89298100
H	16.01266200	-3.79875300	5.69180700
H	17.57732700	-4.05178700	6.45766700
Mg	12.63439100	0.74396500	9.46599900
N	11.45826500	2.39240600	9.22801000
N	11.09908200	-0.60634400	9.52633500
C	9.40142500	1.13109300	9.52474900
H	8.32835100	1.26761000	9.59430800
C	10.13493300	2.32352200	9.33574300
C	9.82794100	-0.21177300	9.57453500
C	9.31621500	3.59310900	9.23933300
H	9.44087000	4.06615100	8.25823300
H	8.25374400	3.39430400	9.38578800
H	9.63576100	4.32680300	9.98793000
C	8.73229800	-1.25150200	9.67709000
H	7.74792600	-0.78745500	9.75020400
H	8.73298600	-1.90853900	8.79954400
H	8.87439100	-1.89441200	10.55334100

N	13.59880900	0.67705200	11.45917900
C	13.59786600	1.89174200	12.21060200
C	12.51148000	2.21923400	13.02813100
C	14.66874600	2.78408300	12.09508500
C	12.49976400	3.42602900	13.72284000
H	11.67413600	1.53056600	13.11406500
C	14.64532300	3.99111300	12.79053800
H	15.52153800	2.51528000	11.47791800
C	13.56380900	4.31994900	13.60640600
H	11.65175300	3.66703000	14.35812900
H	15.48617900	4.67344700	12.69951900
H	13.55250700	5.25969200	14.15052400
Mg	15.88218700	-0.17478100	8.71040600
H	15.95475700	-0.09844600	10.51433300
H	14.06577500	0.42328500	8.41355500
H	14.56865000	0.37045500	11.20287600
C	15.72284100	-3.18311200	8.31959200
H	14.91160200	-3.63344400	7.72694400
H	16.49640300	-3.95323200	8.45220200
H	15.31957300	-2.96868800	9.31521500
C	17.36259900	2.31930100	7.80224800
H	18.43494500	2.55174400	7.87719600
H	16.92489200	3.02142400	7.07653400
H	16.92078700	2.54815300	8.77789300
C	11.39003700	-2.03168700	9.52446700
H	11.07354300	-2.53889600	10.44853600
H	10.92251500	-2.56430100	8.68423000
H	12.47055800	-2.17954600	9.42770600
C	12.07641000	3.68911900	8.99309000
H	11.70746400	4.17883700	8.08103500
H	11.93099200	4.38629800	9.83008700
H	13.15584800	3.55726900	8.86972400
H	13.16017100	-0.07364300	11.98714800

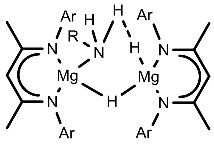

TS

N	17.08906600	0.99934800	8.05586700
N	15.48347000	-1.46294500	7.29256300
C	17.69758500	-0.80737000	6.54348800
H	18.50379000	-1.13198400	5.89588900
C	17.93594100	0.41146400	7.21275400
C	16.58028800	-1.66674600	6.56673200
C	19.26888600	1.06888200	6.92477500
H	19.84100700	0.50189700	6.18942100
H	19.87277800	1.15071500	7.83594100
H	19.13287600	2.08681400	6.54254700
C	16.66696800	-2.89164300	5.68191500
H	17.63440500	-2.95436500	5.18214900
H	15.88720200	-2.87523300	4.91155600

H	16.52018600	-3.80944600	6.26241400
Mg	12.25805800	0.92547400	9.77727700
N	10.75419600	2.21801600	9.27539400
N	11.07868200	-0.74927300	9.84837700
C	9.11949300	0.43251100	9.03425400
H	8.09044200	0.26367000	8.73851400
C	9.53881800	1.77916200	8.96046800
C	9.80338200	-0.72150000	9.46749500
C	8.49076200	2.76809400	8.49490400
H	8.83652700	3.32569500	7.61713000
H	7.55784800	2.26637900	8.23497900
H	8.27336700	3.50754700	9.27440700
C	8.99186800	-1.99964300	9.49491000
H	7.97135400	-1.83075600	9.14906500
H	9.44470700	-2.77030100	8.86045800
H	8.94313300	-2.41279800	10.50911300
N	13.50200000	0.99523900	11.44957100
C	14.03374700	2.18594000	11.94798000
C	14.19317300	2.43359800	13.32574000
C	14.46784500	3.18821800	11.05517700
C	14.74431800	3.62566800	13.78287000
H	13.87187500	1.67669700	14.03903000
C	15.02229100	4.37849700	11.52055100
H	14.35586000	3.02723600	9.98396600
C	15.16561200	4.61065800	12.88759600
H	14.84705600	3.78620500	14.85341500
H	15.34213600	5.13168100	10.80421700
H	15.59554700	5.53971000	13.24938400
Mg	15.29418600	0.15215400	8.51695000
H	15.29053200	-0.22028200	10.43814800
H	13.62880700	0.89951000	8.46903300
H	14.60731000	0.24347700	10.81570900
C	14.38769100	-2.41415100	7.19145300
H	14.03638900	-2.55574700	6.15991400
H	14.64525900	-3.40595900	7.59176500
H	13.53455000	-2.04179500	7.76693700
C	17.48723100	2.25382600	8.67966500
H	18.44365200	2.17997900	9.21492100
H	17.57645600	3.07676400	7.95532700
H	16.73663300	2.55006400	9.41785100
C	11.63597700	-2.00596100	10.32273300
H	11.13407800	-2.38324000	11.22621800
H	11.59782000	-2.80932700	9.57246300
H	12.69028300	-1.86150500	10.58110900
C	11.02360100	3.64581100	9.17823700
H	10.96604900	4.01915800	8.14516000
H	10.33903400	4.25203700	9.78745400
H	12.03382800	3.85166000	9.54273200
H	13.21377500	0.40210400	12.22543800

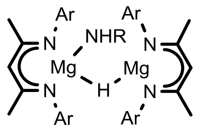

(+H2) IRC-forward

N	16.93054000	1.18491400	8.12368400
N	15.25058800	-1.21084300	7.30840300
C	17.62596800	-0.84664200	6.97300200
H	18.50430200	-1.31591900	6.54557700
C	17.85590500	0.42778100	7.53319700
C	16.43860500	-1.59488000	6.84382100
C	19.27504400	0.94311600	7.43432700
H	19.91861200	0.23722100	6.90821400
H	19.70041200	1.11755900	8.42918600
H	19.30974300	1.90077300	6.90313200
C	16.55758500	-2.91259300	6.11097200
H	17.59021500	-3.11859000	5.82680700
H	15.94825400	-2.91093700	5.19965400
H	16.19944400	-3.74269500	6.73017400
Mg	12.09747500	1.02304500	9.90327600
N	10.50215900	2.20298700	9.39236100
N	11.06464400	-0.74738900	9.80398000
C	9.12554400	0.29493800	8.77973600
H	8.17154500	0.05116400	8.32635800
C	9.39145300	1.67889700	8.88424700
C	9.85759600	-0.81561800	9.24573400
C	8.29352100	2.59848600	8.39216000
H	8.67166300	3.30169300	7.64166500
H	7.47051200	2.03537900	7.95012000
H	7.88932300	3.20006700	9.21468600
C	9.17980600	-2.16227000	9.10129600
H	8.21690800	-2.07139300	8.59697000
H	9.80137300	-2.86158300	8.53022400
H	9.00663200	-2.62120900	10.08187600
N	13.38002800	1.17012300	11.45828100
C	14.18731300	2.22587500	11.80905300
C	14.89622800	2.28086500	13.03537300
C	14.36480300	3.32912600	10.93437600
C	15.70344600	3.36238400	13.36430600
H	14.78993500	1.45419900	13.73653500
C	15.17576500	4.40898000	11.27457600
H	13.83372600	3.34126000	9.98340500
C	15.85719300	4.44264700	12.49106900
H	16.22053500	3.36283900	14.32155600
H	15.26767600	5.24026500	10.57810900
H	16.48346800	5.28892800	12.75614600
Mg	15.03943000	0.50810600	8.35569900
H	15.57940400	-0.51358400	10.36415900
H	13.39854000	1.15923400	8.46102700
H	14.98771000	-0.12403600	10.64819600

C	14.08943200	-2.05350600	7.06116500
H	13.91260100	-2.22997600	5.99110000
H	14.16483600	-3.03615700	7.54915400
H	13.19664900	-1.55965500	7.45675500
C	17.32315300	2.49211400	8.63869300
H	18.14942100	2.43359300	9.35918300
H	17.62629300	3.18337000	7.83941300
H	16.48096800	2.94557700	9.16756500
C	11.65774400	-1.96510000	10.33251100
H	11.06211700	-2.41422000	11.14092600
H	11.80864600	-2.74366300	9.57006200
H	12.64119600	-1.73264600	10.75345000
C	10.59956000	3.65107100	9.50662500
H	10.60964100	4.15611100	8.52904300
H	9.78022300	4.08876100	10.09380200
H	11.52964200	3.91420500	10.01837300
H	13.28547300	0.53502500	12.24580500

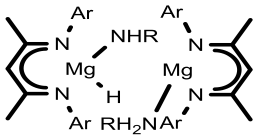

IRC-reverse

N	16.86592100	2.36498600	8.52455900
N	16.81103000	0.19843300	6.42498200
C	18.27388900	0.39296100	8.34964200
H	19.09933400	-0.10630500	8.84403600
C	17.87650700	1.60938100	8.94472700
C	17.83544300	-0.22441300	7.16012500
C	18.70333900	2.06397300	10.12937900
H	19.21570300	3.00855900	9.91173800
H	19.45973300	1.32371000	10.39371900
H	18.07112300	2.23831000	11.00724900
C	18.63210100	-1.43134300	6.70842200
H	19.37833800	-1.71526400	7.45192800
H	19.15585200	-1.22492400	5.76736400
H	17.98062700	-2.29375400	6.52722600
N	11.81828500	3.84018800	8.46397300
N	10.30332800	1.20860300	8.44703100
C	9.72565500	3.28281000	9.57126300
H	8.96486500	3.71537900	10.21066700
C	10.76975200	4.16219400	9.22258800
C	9.50349100	1.93166300	9.22415200
C	10.65415500	5.57073000	9.76583900
H	10.59444800	6.30404200	8.95304500
H	9.76724200	5.68498500	10.39032900
H	11.53232200	5.83552900	10.36560500
C	8.25352500	1.29898000	9.79711900
H	7.69640600	2.00463700	10.41464400
H	7.58990200	0.94347100	9.00051400
H	8.50331600	0.42836200	10.41420800
N	13.75362100	0.71250700	7.98362200

C	13.90122000	0.18868400	9.28570700
C	13.99556500	-1.19160000	9.54151500
C	13.95420500	1.06084100	10.38954700
C	14.13706700	-1.67450300	10.83926500
C	14.09255700	0.57161900	11.68522300
C	14.18616400	-0.79935000	11.92443800
H	14.20967400	-2.74719700	11.00176600
H	14.12706500	1.27218000	12.51611900
H	13.67062600	-0.07839800	7.34207400
H	14.29530700	-1.17844700	12.93612100
H	13.88557900	2.13331700	10.21954100
H	13.96146100	-1.88874900	8.70595200
C	12.80302500	4.87074300	8.16449600
H	12.36730700	5.75084100	7.66909400
H	13.33051300	5.23296200	9.05929300
H	13.55856400	4.46306500	7.48389600
C	9.95363500	-0.17961800	8.18327700
H	10.69904100	-0.62022100	7.51433700
H	9.93712500	-0.79315000	9.09495700
H	8.97829300	-0.28861100	7.68857500
C	16.54764900	-0.45386800	5.15475600
H	17.42818400	-0.49531000	4.49783800
H	16.17770900	-1.48611000	5.26455800
H	15.78350600	0.11551300	4.61475100
C	16.64537200	3.64770300	9.17057200
H	16.35400200	3.55316400	10.22764400
H	17.52567500	4.30556700	9.13140800
H	15.83591300	4.17452400	8.65589200
Mg	15.52936800	1.65757800	7.12667900
Mg	12.08099800	1.93702900	7.75558700
N	12.11810900	2.28805100	5.58062300
C	11.55090300	1.30025900	4.72482800
C	10.21113100	1.39018700	4.33235500
C	12.31394200	0.20182600	4.31470600
C	9.64619200	0.39485100	3.53911400
H	9.61233200	2.23921100	4.65384000
C	11.73966100	-0.79036300	3.52299300
H	13.35923800	0.13745000	4.60284900
C	10.40489500	-0.70150900	3.13113100
H	8.60536900	0.47975600	3.23897800
H	12.34580600	-1.63432700	3.20511600
H	9.96218700	-1.47514900	2.51110800
H	14.71463700	2.70420100	5.88338900
H	13.15138100	2.40882400	5.47322600
H	11.68084700	3.19510300	5.43943600

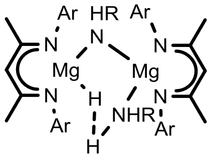

TS

N	16.78222700	2.29175700	8.27645100
N	16.46945600	-0.13062600	6.46319700
C	18.40206700	0.54965100	7.77127500
H	19.41884700	0.24979400	7.99654700
C	17.99281700	1.74680700	8.39322700
C	17.73091500	-0.29057200	6.85773200
C	19.03912200	2.44365700	9.23617800
H	19.27940100	3.43188900	8.82698900
H	19.96216600	1.86457000	9.28239700
H	18.68096000	2.60111600	10.25972300
C	18.54327400	-1.43737100	6.29580200
H	19.53595500	-1.47925700	6.74569500
H	18.66643400	-1.33561000	5.21127900
H	18.04567700	-2.39800200	6.46988500
N	11.60531900	3.68446800	8.73759400
N	10.03923400	1.09402900	8.48640700
C	9.68662700	2.93902000	10.02863300
H	9.03239900	3.25447200	10.83315700
C	10.67285100	3.87789100	9.67084700
C	9.36767800	1.67989800	9.47167300
C	10.62814400	5.19682800	10.41377400
H	10.43616700	6.02929500	9.72655800
H	9.84449200	5.19945600	11.17245000
H	11.58367200	5.40754100	10.90766700
C	8.15147800	0.99230200	10.05595100
H	7.73873200	1.55741100	10.89261000
H	7.36399900	0.87945900	9.30158800
H	8.39899000	-0.01454000	10.41035900
N	13.51198200	0.65822300	8.24748200
C	13.69650300	0.33447700	9.61502400
C	13.74609300	-0.99463200	10.07176600
C	13.84505100	1.35937900	10.56592100
C	13.93692400	-1.28403900	11.42002100
C	14.03800100	1.06365100	11.91253300
C	14.08763000	-0.25855800	12.35317400
H	13.96957400	-2.32211000	11.74141000
H	14.14338100	1.87810200	12.62502100
H	13.32512600	-0.22090400	7.76053100
H	14.23642000	-0.48573800	13.40448300
H	13.78264100	2.39408200	10.23895600
H	13.63481200	-1.80717700	9.35552900
C	12.50012500	4.78441200	8.41812900
H	11.97093600	5.68340300	8.06894300
H	13.13195900	5.09323800	9.26516500
H	13.17312500	4.48317800	7.60804400
C	9.54921000	-0.17574100	7.97064300
H	10.14789600	-0.47259300	7.10406300
H	9.61275400	-0.98882100	8.70924900
H	8.50672600	-0.12413200	7.62702200
C	15.93316800	-1.03535300	5.45659600

H	16.53052000	-1.04827400	4.53482100
H	15.85753400	-2.07340000	5.81357200
H	14.92678200	-0.71329700	5.17218300
C	16.53013600	3.56873400	8.92544900
H	16.58335500	3.50833100	10.02177100
H	17.22397900	4.35690200	8.60123800
H	15.52236700	3.91138500	8.67179300
Mg	15.29161600	1.28900100	7.32605600
Mg	11.80192500	1.87276000	7.78517700
N	12.18273300	2.23780900	5.77006400
C	11.91939400	1.39441000	4.69396600
C	11.50355400	1.87565600	3.43443300
C	12.10084100	0.00006200	4.81601000
C	11.27577700	1.01239400	2.36901700
H	11.35628000	2.94632000	3.30355300
C	11.87584000	-0.85980400	3.74356900
H	12.40832800	-0.41209700	5.77488800
C	11.46044700	-0.36467000	2.50872400
H	10.95091600	1.42179400	1.41540200
H	12.02232600	-1.92887600	3.87972000
H	11.28129700	-1.03567300	1.67422600
H	14.58629200	2.45773000	5.92904700
H	13.71680100	2.35418800	5.89754900
H	11.92431900	3.18899700	5.51121900

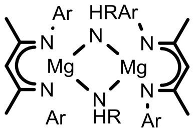

(+H2) IRC-forward

N	16.30718800	2.39145800	8.56035200
N	16.44167300	0.13108900	6.51913000
C	18.26823500	1.34811600	7.56294600
H	19.35054600	1.39890400	7.57712500
C	17.62954500	2.27050000	8.41445900
C	17.73937600	0.37347900	6.68827400
C	18.54195800	3.17854300	9.20905100
H	18.35047100	4.23220600	8.97608000
H	19.59152200	2.97005400	8.99918200
H	18.37778100	3.05655200	10.28574100
C	18.75021100	-0.42971800	5.90080600
H	19.77157900	-0.14757300	6.15863600
H	18.61575200	-0.27995500	4.82341800
H	18.63193000	-1.50276300	6.08834700
N	11.87229000	3.85510200	8.43673000
N	10.03874000	1.49919500	8.91024400
C	10.44403800	3.41063000	10.35107800
H	10.13487400	3.83791800	11.29840500
C	11.31996000	4.21427000	9.59734600
C	9.81072800	2.18838700	10.01963300
C	11.61080500	5.58819800	10.16610600

H	11.21867400	6.37288500	9.50811600
H	11.15484800	5.71637900	11.14880500
H	12.68834900	5.76555100	10.26040700
C	8.78924700	1.68326800	11.01639500
H	8.74467800	2.32070900	11.90053400
H	7.78802800	1.65224800	10.57085300
H	9.02669400	0.66319200	11.33821300
N	13.17799900	0.49852900	8.03055100
C	13.16902800	-0.22622000	9.24767000
C	13.08764200	-1.63044800	9.28393400
C	13.25417000	0.45865400	10.47307800
C	13.09665600	-2.31754400	10.49504200
C	13.26923600	-0.23553100	11.68002900
C	13.19283100	-1.62803900	11.70367400
H	13.03153000	-3.40277700	10.49079800
H	13.33045000	0.32186600	12.61138100
H	12.96702900	-0.15099600	7.26881700
H	13.20256600	-2.16602300	12.64685600
H	13.27457300	1.54583100	10.47140300
H	13.01718100	-2.18259400	8.34849600
C	12.61794000	4.85684800	7.69044200
H	12.00288600	5.72547700	7.41255600
H	13.49682800	5.24363600	8.22768300
H	12.98055500	4.41350600	6.75812900
C	9.26084600	0.29498200	8.66239100
H	9.49137700	-0.08473500	7.66207200
H	9.48635500	-0.50911400	9.37783900
H	8.17652300	0.47099900	8.69041300
C	16.03239000	-0.88777700	5.55966200
H	16.40093100	-0.68473100	4.54561600
H	16.37272900	-1.89345400	5.84346500
H	14.94075800	-0.91841900	5.49600200
C	15.79430500	3.39352400	9.48461300
H	16.09382000	3.20315500	10.52473000
H	16.11645800	4.41252400	9.22854200
H	14.70071200	3.38570200	9.45819700
Mg	15.06047200	1.15067000	7.57862800
Mg	11.61256100	1.95552100	7.67034500
N	11.75447900	1.95981000	5.64591000
C	11.92342300	0.95388500	4.72717400
C	12.33126700	1.19344100	3.38914000
C	11.72057000	-0.40636800	5.07927400
C	12.53965500	0.15556400	2.48993500
H	12.48212700	2.22307500	3.06738700
C	11.94206200	-1.44067000	4.17356200
H	11.31771100	-0.64029900	6.06381400
C	12.36092300	-1.17778100	2.86917700
H	12.84959600	0.39189300	1.47403800
H	11.75994900	-2.46643800	4.48830500
H	12.52350800	-1.98540000	2.16204000

H	14.72334400	2.42193100	5.71313300
H	13.97123000	2.31249600	5.77593000
H	11.73432600	2.85693600	5.16866900

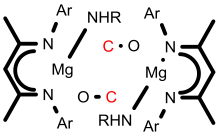

IRC-reverse

N	17.54426600	-1.06979900	9.27634500
N	17.29604500	0.20073200	6.51769700
C	19.13887400	0.33774800	8.10230300
H	20.12496800	0.77343000	8.21306900
C	18.72521800	-0.45846900	9.18971000
C	18.50930700	0.62132200	6.87020900
C	19.72271200	-0.61289800	10.31680900
H	19.30121800	-0.26860400	11.26813000
H	20.63424400	-0.04643300	10.12304700
H	19.99807000	-1.66488400	10.45447300
C	19.31306800	1.45596200	5.89708600
H	20.25729200	1.78025900	6.33609000
H	18.75396800	2.34504800	5.58546200
H	19.53904300	0.88788800	4.98718000
N	9.25161400	0.21671300	8.92491400
N	10.57682500	0.39315300	11.66026600
C	8.55999900	-0.80173300	11.02456200
H	7.77884400	-1.42621200	11.44212300
C	8.41186100	-0.52089000	9.64832100
C	9.51833100	-0.35890700	11.95873000
C	7.18261200	-1.10499900	8.98644000
H	6.50587800	-0.31211500	8.64703800
H	7.45203800	-1.69323100	8.10247800
H	6.63000700	-1.74846500	9.67207500
C	9.29172000	-0.78290900	13.39352300
H	9.17650600	0.08974400	14.04679200
H	8.39730400	-1.39940100	13.49046000
H	10.14488600	-1.35477100	13.77626100
N	14.65981300	-2.13457000	7.64281200
C	13.71036700	-2.24776200	6.64717000
C	13.10873100	-3.48063300	6.30420700
C	13.26987800	-1.10493500	5.94135000
C	12.14628800	-3.56355900	5.30481600
C	12.30778100	-1.19829100	4.93994700
C	11.73470200	-2.42618900	4.60684800
H	11.71431700	-4.53269400	5.06547200
H	12.00181200	-0.29657000	4.41416500
Mg	10.99366200	0.86856300	9.73122600
Mg	16.13767000	-0.80152000	7.84192000
N	12.22843800	2.39412700	9.35637900
C	12.52204300	3.06322000	8.18637200
C	12.33457000	2.44140700	6.92999700
C	13.05769000	4.37242600	8.17305800

C	12.64007800	3.09771700	5.74233800
C	13.36140000	5.02015900	6.98055200
C	13.15552100	4.39502800	5.74968900
O	15.06453100	0.98000700	8.89993700
C	14.78895400	1.75928700	9.69811100
H	12.47364100	2.58641200	4.79666400
H	13.76316000	6.03042200	7.01531500
H	12.41433000	3.00321300	10.14794600
H	14.84585300	-3.04944200	8.04390900
O	12.38402900	-0.94670400	9.36054300
C	13.09315400	-1.80241000	9.65975200
H	13.38934800	4.90558200	4.82040600
H	11.95362200	1.42242700	6.89495200
H	13.22059700	4.88223700	9.12124100
H	10.98758100	-2.49597500	3.82205400
H	13.42015400	-4.38161000	6.83052700
H	13.68419400	-0.13056900	6.19461200
C	8.93093400	0.47334400	7.52617300
H	9.01006400	-0.42924900	6.90375500
H	7.92318000	0.88734600	7.39008600
H	9.63282100	1.20823100	7.12271800
C	11.46670200	0.81067200	12.73291100
H	10.96795000	1.44590200	13.47900100
H	11.91718200	-0.03516000	13.27230400
H	12.29018500	1.39633100	12.31199500
C	16.80146700	0.52440500	5.18481500
H	16.63369800	1.60195400	5.04681500
H	17.47880300	0.18779200	4.38856800
H	15.84331100	0.02329400	5.02136100
C	17.26140000	-1.89238900	10.44329700
H	17.94852600	-2.74510600	10.54121700
H	17.30398000	-1.32824800	11.38626100
H	16.24996500	-2.30210700	10.36043200

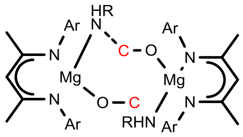

TS

N	17.35189300	-0.90743000	9.36088900
N	17.36406700	0.21097100	6.53796700
C	19.13489600	0.26757700	8.20367500
H	20.15268500	0.61290700	8.34441400
C	18.58619700	-0.41298600	9.31054100
C	18.59584200	0.53138500	6.92609900
C	19.50251800	-0.58367900	10.50352600
H	19.05484900	-0.15862300	11.40903000
H	20.46607700	-0.09985100	10.33846200
H	19.68602300	-1.64481400	10.70923700
C	19.52550800	1.21907100	5.94861700
H	20.47946700	1.46881400	6.41493300
H	19.07961400	2.14202900	5.56124600

H	19.72824200	0.57756100	5.08304900
N	9.45610500	-0.05416700	8.97415800
N	10.90894100	0.15653300	11.65058500
C	8.53007900	-0.09993600	11.22635700
H	7.58427500	-0.19417400	11.74693000
C	8.43032500	-0.15386200	9.81974700
C	9.65393500	0.01022800	12.07180900
C	7.03653700	-0.35852800	9.26699100
H	6.77882200	0.42327500	8.54411100
H	6.96088000	-1.31653900	8.73945300
H	6.28918800	-0.35152600	10.06115200
C	9.37534800	-0.05158800	13.55756700
H	9.71742700	0.85944700	14.06195000
H	8.31049700	-0.17343900	13.75890100
H	9.90877300	-0.88924100	14.02115600
N	14.73134900	-2.13056400	7.47686100
C	13.91267100	-2.26868600	6.36876300
C	13.52794800	-3.52881900	5.85713200
C	13.38254300	-1.12668400	5.72725300
C	12.67703900	-3.63504100	4.76340000
C	12.53053500	-1.24314600	4.63171200
C	12.16633100	-2.49564300	4.13742900
H	12.41005800	-4.62229700	4.39346600
H	12.15335100	-0.34141400	4.15404800
Mg	11.31502000	0.31730000	9.67509300
Mg	16.02112400	-0.57714600	7.85407700
N	12.58573900	1.94561000	9.30205400
C	12.42945100	2.84990300	8.23078400
C	12.21207400	2.36788700	6.93161100
C	12.54427200	4.23797200	8.41837800
C	12.10397700	3.24845300	5.85723900
C	12.42508200	5.11183100	7.34358000
C	12.20600500	4.62400000	6.05406200
O	14.63630300	0.92993700	8.35266700
C	14.17344900	1.08788200	9.45204800
H	11.93377200	2.85308400	4.85925100
H	12.50681900	6.18206800	7.51441800
H	12.61398000	2.45739700	10.18292500
H	15.08993100	-3.04197000	7.74979600
O	12.48259800	-1.38094500	9.06935000
C	13.26042400	-2.23727800	9.22332600
H	12.11543200	5.30818600	5.21567600
H	12.12379200	1.29816600	6.76582900
H	12.72162300	4.62830300	9.41866000
H	11.50535200	-2.58321700	3.28043000
H	13.91810700	-4.42907200	6.32876600
H	13.66254500	-0.14130200	6.09216300
C	9.20083300	-0.19402400	7.54580000
H	8.66318300	-1.11873800	7.29905600
H	8.62594900	0.64787300	7.13471000

H	10.15053700	-0.23221700	7.00496200
C	11.98240700	0.22146000	12.63318900
H	11.86239400	1.05202200	13.34348200
H	12.07342500	-0.70274700	13.22142100
H	12.93622700	0.37143100	12.11704300
C	16.96400900	0.50593400	5.16922100
H	16.92336000	1.58543200	4.96188300
H	17.62946200	0.05100000	4.42235000
H	15.96333700	0.10297100	4.98830800
C	16.92989500	-1.61856600	10.55783800
H	17.57304900	-2.47847300	10.79441300
H	16.89933000	-0.97412500	11.44848600
H	15.91786500	-2.00799700	10.40973100

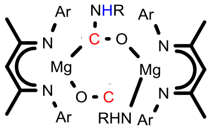

IRC-forward

N	16.93838600	-0.52514700	9.31729500
N	16.68033900	0.20290500	6.38694700
C	18.49907200	0.70382900	7.91659900
H	19.46794200	1.18897000	7.95149300
C	18.09139200	0.11277900	9.13079300
C	17.87771600	0.71649200	6.64867000
C	19.07509800	0.22064100	10.27765100
H	18.61242300	0.68794700	11.15435900
H	19.94935000	0.80986900	9.99766300
H	19.42191800	-0.77054600	10.59297600
C	18.67344500	1.35725500	5.53041400
H	19.60716100	1.78381700	5.89952100
H	18.10127600	2.15280400	5.03976200
H	18.91919400	0.62198400	4.75521200
N	9.50758400	-0.76129600	8.83628500
N	10.93915800	-0.08361100	11.41571200
C	8.57423900	-0.50100300	11.06757400
H	7.63272700	-0.54794800	11.60239500
C	8.47952700	-0.75742000	9.68414900
C	9.69248100	-0.19192900	11.86904500
C	7.08885700	-1.04627700	9.16121400
H	6.80649300	-0.33582900	8.37598300
H	7.03577100	-2.04651400	8.71611500
H	6.34479200	-0.98857300	9.95650200
C	9.41347900	0.03148500	13.33967000
H	9.69044600	1.04745600	13.64460100
H	8.35794800	-0.11553500	13.57177600
H	9.99934000	-0.65595700	13.96030200
N	14.63673400	-2.44764300	7.61513600
C	13.81937900	-2.76938100	6.53969000
C	13.79055000	-4.06114300	5.96827800
C	12.94436900	-1.80285900	5.99549000
C	12.94996900	-4.35998100	4.90332700

C	12.10725400	-2.11210600	4.92472200
C	12.09870600	-3.39000800	4.36792400
H	12.96088200	-5.36311700	4.48358200
H	11.46191100	-1.33799500	4.51512300
Mg	11.41703300	-0.44602200	9.46616100
Mg	15.48829800	-0.56891800	7.86704900
N	12.46544700	2.32221800	8.58020100
C	13.10659400	3.51320900	8.17073900
C	14.41054700	3.56759700	7.65803300
C	12.36947900	4.70094700	8.29590000
C	14.94678100	4.79871400	7.28438400
C	12.91816900	5.92039600	7.91838000
C	14.21468200	5.97731700	7.40846800
O	14.02873400	0.74866600	8.20907900
C	12.85806800	1.01213300	8.61874900
H	15.95928700	4.82860300	6.89089100
H	12.32911300	6.82725200	8.02388700
H	11.52775500	2.47075700	8.92721300
H	15.28248000	-3.21261000	7.79427200
O	12.58020800	-2.19573900	9.38378300
C	13.57396800	-2.82342800	9.42206100
H	14.64756400	6.92841700	7.11279300
H	14.98882100	2.66069500	7.55725000
H	11.35631800	4.66470200	8.69268400
H	11.44849200	-3.62544900	3.53076800
H	14.45104400	-4.82790100	6.36895300
H	12.95241700	-0.79505600	6.40438600
C	9.24787700	-1.04961300	7.43152700
H	8.76724400	-2.02537000	7.27854600
H	8.61382400	-0.28958200	6.95202400
H	10.19378600	-1.07916300	6.88439400
C	11.99951600	0.25167800	12.35454400
H	11.82083300	1.19730700	12.88539800
H	12.15860400	-0.52742100	13.11416900
H	12.94182300	0.36766000	11.80949800
C	16.18887200	0.23891500	5.01686100
H	16.02547700	1.26265200	4.64851200
H	16.86372000	-0.26145200	4.30800700
H	15.22875000	-0.28215700	4.96091000
C	16.67927100	-1.12570700	10.61642000
H	17.46456000	-1.82752900	10.93164100
H	16.56672400	-0.38015300	11.41852400
H	15.74770100	-1.69905400	10.57250300

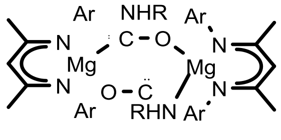

IRC-reverse

N	16.96214200	-0.50287700	9.39663700
N	16.72906000	0.12245600	6.44059800
C	18.52783100	0.68986200	7.97086900

H	19.49258000	1.18355700	7.99897900
C	18.11172900	0.13828000	9.20055600
C	17.91962900	0.65435700	6.69704100
C	19.08164400	0.29459000	10.35359100
H	18.60523300	0.78749700	11.20866000
H	19.95372300	0.88186000	10.06290300
H	19.43364500	-0.68198700	10.70653200
C	18.72207500	1.26356000	5.56603200
H	19.64808300	1.71065000	5.93023200
H	18.14868500	2.03690400	5.04245400
H	18.98241700	0.50454900	4.81894000
N	9.55366200	-0.85069800	8.90482200
N	10.98185900	-0.09088300	11.46320800
C	8.61949100	-0.53347200	11.12828900
H	7.67854300	-0.57148300	11.66481800
C	8.52599800	-0.83036800	9.75290300
C	9.73605700	-0.19458900	11.92003500
C	7.13736100	-1.14475700	9.23942400
H	6.84881100	-0.45879000	8.43489500
H	7.09193000	-2.15740800	8.82247200
H	6.39339200	-1.07073600	10.03345300
C	9.45633300	0.06818500	13.38400700
H	9.73078200	1.09274100	13.66102800
H	8.40119700	-0.07522800	13.62012700
H	10.04399600	-0.60056600	14.02311500
N	14.70456300	-2.50926800	7.73267900
C	13.89356400	-2.87421700	6.66621600
C	13.88313700	-4.18299300	6.13421300
C	13.00656000	-1.93652700	6.09182600
C	13.04823600	-4.52547400	5.07792200
C	12.17521400	-2.28956300	5.03016800
C	12.18471800	-3.58384100	4.51247600
H	13.07331300	-5.54067200	4.68890200
H	11.52012800	-1.53708000	4.59641100
Mg	11.46056300	-0.50006400	9.52331300
Mg	15.52894500	-0.61244700	7.93307700
N	12.45546300	2.25119100	8.53650200
C	13.07008100	3.43878700	8.07921500
C	14.37284200	3.50160500	7.56448300
C	12.30643200	4.61373700	8.15654700
C	14.88153500	4.72829500	7.14111700
C	12.82785600	5.82892200	7.72986900
C	14.12305600	5.89420600	7.21762700
O	14.04973000	0.69674900	8.21937400
C	12.87481600	0.95166200	8.62246200
H	15.89333100	4.76503100	6.74637000
H	12.21855300	6.72586600	7.79874500
H	11.51535200	2.39315800	8.87978600
H	15.36127100	-3.25929300	7.93385600
O	12.64311800	-2.23867600	9.49584700

C	13.63973100	-2.86059700	9.54654000
H	14.53464300	6.84203200	6.88351500
H	14.97120200	2.60446000	7.50048000
H	11.29408700	4.57076700	8.55473400
H	11.53886800	-3.85340300	3.68225400
H	14.55341900	-4.92809900	6.55884200
H	13.00069400	-0.91665200	6.46956300
C	9.29584900	-1.18150100	7.50908000
H	8.82318500	-2.16517100	7.38461200
H	8.65526400	-0.44084900	7.00839000
H	10.24179600	-1.21886200	6.96243100
C	12.04041300	0.27621600	12.39220000
H	11.85762300	1.23638200	12.89483200
H	12.20206300	-0.47953500	13.17448300
H	12.98268100	0.37989800	11.84460800
C	16.25158500	0.10906700	5.06512700
H	16.08852500	1.11872800	4.65971300
H	16.93552000	-0.41293900	4.38097300
H	15.29388000	-0.41733000	5.01744500
C	16.69304100	-1.05907700	10.71324900
H	17.48304600	-1.73771000	11.06515200
H	16.55870600	-0.28618900	11.48564000
H	15.77025600	-1.64703000	10.67767700

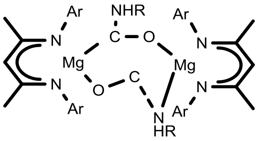

TS

N	16.98241900	-0.47217500	9.40873700
N	16.69990800	0.08591500	6.43936900
C	18.55060500	0.63262500	7.91621100
H	19.53034900	1.09650500	7.91022800
C	18.14551100	0.12801400	9.17010000
C	17.91118400	0.58981400	6.65899500
C	19.14395500	0.28894400	10.29759500
H	18.70059200	0.82136400	11.14664600
H	20.02673800	0.84046900	9.97135900
H	19.47364800	-0.68689900	10.67306900
C	18.70131300	1.15932000	5.49903100
H	19.64979100	1.58215800	5.83304800
H	18.13812500	1.94424200	4.98158100
H	18.91863300	0.38447700	4.75445200
N	9.53123200	-0.81031500	8.89855900
N	10.95999100	-0.14254300	11.47879600
C	8.58662600	-0.49415700	11.11700300
H	7.64004200	-0.50732800	11.64483300
C	8.49673900	-0.77026500	9.73735000
C	9.70794200	-0.20757800	11.92337200
C	7.10392400	-1.03692000	9.20780000
H	6.84149200	-0.33147500	8.41111400
H	7.03324700	-2.04183900	8.77584900

H	6.35556400	-0.95320100	9.99678000
C	9.42447800	0.04232900	13.38907400
H	9.73616800	1.05072200	13.68530400
H	8.36226400	-0.06329300	13.61326100
H	9.97941200	-0.65910400	14.02241500
N	14.68513300	-2.51272300	7.82865100
C	13.92390500	-2.87858500	6.71447600
C	13.98954100	-4.16977200	6.15271600
C	13.01992200	-1.96027900	6.14437900
C	13.20227500	-4.51715400	5.06169900
C	12.23278800	-2.31861000	5.05048300
C	12.31520500	-3.59582300	4.49965700
H	13.28220500	-5.51802700	4.64469900
H	11.55682000	-1.58420300	4.61862100
Mg	11.44801800	-0.53349800	9.53477900
Mg	15.52246700	-0.58444500	7.97614800
N	12.42716500	2.24661700	8.56174600
C	13.03449200	3.43846000	8.10616400
C	14.33216600	3.50799700	7.57961400
C	12.26812600	4.61079600	8.19550600
C	14.83348400	4.73792400	7.15693200
C	12.78195100	5.82942100	7.76938400
C	14.07220500	5.90113900	7.24559500
O	14.03128800	0.70160900	8.24879900
C	12.85174000	0.94985300	8.64942500
H	15.84144800	4.77946700	6.75288400
H	12.17055600	6.72415600	7.84787800
H	11.48602300	2.38175600	8.90496500
H	15.37871800	-3.23071300	8.02562800
O	12.64406700	-2.20146300	9.35480400
C	13.72796700	-2.67539800	9.50001300
H	14.47777700	6.85172200	6.91196800
H	14.93234600	2.61291700	7.50595400
H	11.25976600	4.56294000	8.60304300
H	11.70479700	-3.86905500	3.64413500
H	14.67689500	-4.89736400	6.57998600
H	12.96322700	-0.95305000	6.54939000
C	9.27728000	-1.11967600	7.49788900
H	8.78720900	-2.09296800	7.35781000
H	8.65480300	-0.36166300	7.00002600
H	10.22695600	-1.16809400	6.95834000
C	12.02357500	0.16723800	12.42253400
H	11.87334000	1.12485000	12.94084800
H	12.14976600	-0.60813600	13.19198500
H	12.97388900	0.24339500	11.88463300
C	16.18912100	0.07070400	5.07574400
H	16.04634400	1.07995300	4.66191700
H	16.84050100	-0.47965900	4.38222100
H	15.21566500	-0.42743000	5.05706600
C	16.72094900	-0.97567100	10.74924900

H	17.50291200	-1.65757000	11.11226100
H	16.61805500	-0.17050900	11.49275000
H	15.78521700	-1.54389100	10.74718300

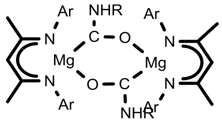

IRC-forward

N	17.08506300	-0.04941500	8.86410300
N	16.20405000	0.51284300	6.03921300
C	18.46274400	0.42751600	6.92379000
H	19.49830600	0.53874100	6.62302900
C	18.27104000	0.12220100	8.28790800
C	17.52229700	0.60086800	5.88656400
C	19.52965400	-0.01578400	9.11850300
H	19.53116700	0.69537900	9.95263600
H	20.42417200	0.16062200	8.51960800
H	19.60587700	-1.01744400	9.55737900
C	18.09530800	0.89918900	4.51696100
H	19.18400200	0.96190400	4.54527800
H	17.70813900	1.84678100	4.12505200
H	17.81791100	0.12206900	3.79508400
N	9.86950200	0.19163900	7.95375600
N	10.75145500	-0.37101700	10.77822100
C	8.49252700	-0.28713300	9.89409800
H	7.45711100	-0.39926000	10.19503100
C	8.68374000	0.01889700	8.53008700
C	9.43331300	-0.46001500	10.93111500
C	7.42488400	0.15637700	7.69978000
H	7.34766700	1.15833000	7.26174500
H	7.42392400	-0.55410500	6.86507100
H	6.53061700	-0.02133700	8.29866400
C	8.86077700	-0.75899700	12.30077100
H	9.13758500	0.01829800	13.02268700
H	7.77213500	-0.82267300	12.27262200
H	9.24885900	-1.70628200	12.69252100
N	14.51170800	-2.74216200	7.48662300
C	14.00487100	-4.05314700	7.35812700
C	14.88637900	-5.03103300	6.87185800
C	12.69062300	-4.41919800	7.68375500
C	14.46874800	-6.34665600	6.71149200
C	12.28653500	-5.74241100	7.51706200
C	13.16210500	-6.71229900	7.03353000
H	15.16824900	-7.08708000	6.33305800
H	11.26541900	-6.01349300	7.77254600
Mg	11.64616800	-0.08508000	8.94979500
Mg	15.30871600	0.22711600	7.86733900
N	12.44297900	2.88424300	9.33004000
C	12.94971800	4.19527900	9.45834000
C	14.26398000	4.56134900	9.13278700
C	12.06810900	5.17320700	9.94434200

C	14.66798000	5.88460900	9.29930100
C	12.48565200	6.48888000	10.10453100
C	13.79230900	6.85453900	9.78257300
O	14.20794200	1.73985500	8.52878400
C	12.98884800	1.70477800	8.90480300
H	15.68911100	6.15569800	9.04388300
H	11.78606300	7.22932400	10.48276500
H	11.47683700	2.79174300	9.61337600
H	15.47790700	-2.64974200	7.20346100
O	12.74679100	-1.59771200	8.28789200
C	13.96593000	-1.56271200	7.91202300
H	14.12205900	7.88182900	9.90694300
H	14.94529800	3.81182200	8.75687700
H	11.04666000	4.89554500	10.19842800
H	12.83229500	-7.73955300	6.90901400
H	15.90781300	-4.75337500	6.61771000
H	12.00938500	-3.66968900	8.05984400
C	9.91657800	0.51319500	6.53601300
H	9.50487500	-0.28527100	5.90138400
H	9.38182700	1.44102600	6.28552900
H	10.95886500	0.65661300	6.23255600
C	11.60346500	-0.55958000	11.94198900
H	11.38701600	0.14853200	12.75501900
H	11.53654500	-1.57372100	12.36275800
H	12.64809700	-0.40141700	11.65408700
C	15.35241300	0.70190300	4.87525600
H	15.42058200	1.71576100	4.45401300
H	15.56811600	-0.00684900	4.06257700
H	14.30755100	0.54513400	5.16308900
C	17.03752000	-0.37023300	10.28200000
H	17.57093700	-1.29872500	10.53289000
H	17.45038200	0.42792000	10.91626000
H	15.99503200	-0.51197900	10.58553100

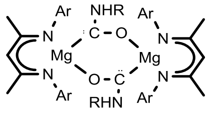

IRC-reverse

Mg	14.92246000	-0.56719900	7.92637000
N	16.59752200	-1.32557700	8.84728900
O	14.31766200	1.10842400	8.77917700
N	15.85264100	-0.22859900	6.12941900
C	18.02803300	-0.96654400	6.91916400
H	19.05736000	-1.06859000	6.59442600
C	17.78749900	-1.35918600	8.25169300
C	17.14811400	-0.44955700	5.94141500
C	18.99173000	-1.85476500	9.02494700
H	18.84867100	-2.88862900	9.36060800
H	19.15624100	-1.25108800	9.92488500
H	19.89834300	-1.81664400	8.41943100
C	17.76789200	-0.13869800	4.59503200
H	18.83799600	-0.35037200	4.58950500

H	17.62651200	0.91539500	4.33000500
H	17.29862300	-0.73071300	3.80069300
Mg	11.48644200	0.31189200	9.53437400
N	9.81197400	1.07339400	8.61501000
N	10.55616700	-0.02890300	11.33089700
C	8.38158300	0.71344100	10.54303500
H	7.35250900	0.81645200	10.86827100
C	8.62224800	1.10784400	9.21104600
N	13.60971100	2.79032800	9.98685300
C	9.26106800	0.19358300	11.51965800
C	13.32655200	1.57928000	9.43576800
C	7.41852200	1.60656600	8.43903500
H	7.25312000	1.00511800	7.53777400
H	6.51195000	1.56824500	9.04460000
H	7.56280900	2.64100700	8.10566800
C	12.79315300	3.60429300	10.79081900
C	13.41187400	4.50384700	11.66869200
C	8.64123700	-0.11867500	12.86569400
H	9.11190400	0.47093500	13.66099000
H	7.57152900	0.09496500	12.87203400
H	8.78074300	-1.17354200	13.12863900
C	12.64666500	5.33621100	12.47964700
H	13.14391200	6.02956100	13.15253500
C	11.39511000	3.56203100	10.72573200
C	11.25452000	5.27645000	12.43565800
C	10.63971000	4.38537600	11.55714900
H	9.55600700	4.33298700	11.50118800
O	12.09057300	-1.36303500	8.67976300
N	12.80038600	-3.04693800	7.47591100
C	13.08271700	-1.83504900	8.02555500
C	13.61822100	-3.86216400	6.67449100
C	13.00089000	-4.76352600	5.79750500
C	13.76736700	-5.59718000	4.98906600
C	15.01617100	-3.81937300	6.74126900
C	15.15943300	-5.53694700	5.03472200
C	15.77285800	-4.64405000	5.91235400
H	13.27116100	-6.29191000	4.31683400
H	16.85647600	-4.59123200	5.96957200
H	11.84073300	-3.36071800	7.61187700
H	14.56911000	3.10426500	9.84981300
C	9.91532500	1.51117800	7.23232400
H	10.96151900	1.45588900	6.91371300
H	9.33586200	0.88341200	6.53924700
H	9.59108300	2.55135100	7.08176900
C	11.34106600	-0.56450200	12.43153600
H	11.01087900	-1.56546600	12.74600300
H	12.38631400	-0.65789700	12.11878000
H	11.33068000	0.08168800	13.32104400
C	16.49441300	-1.76121800	10.23066000
H	17.07324900	-1.13185800	10.92281000

H	15.44813900	-1.70634200	10.54908700
H	16.81955900	-2.80086900	10.38290400
C	15.06729800	0.30401800	5.02764500
H	14.02170000	0.39580100	5.33971100
H	15.39575300	1.30519600	4.71203100
H	15.07939900	-0.34332800	4.13900800
H	15.75843800	-6.18392400	4.40059100
H	15.51337400	-3.15963400	7.44518000
H	11.91507000	-4.80088200	5.74547400
H	10.89684400	2.90377500	10.02118200
H	10.65651800	5.92242200	13.07176000
H	14.49776900	4.54077400	11.71940200

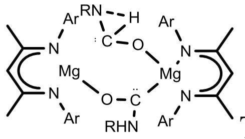

TS

Mg	14.97751400	-0.55005700	7.79735900
N	16.94159300	-1.07827700	7.99292700
O	14.53159400	0.76441800	9.18874300
N	15.11104400	-0.03733600	5.81624900
C	17.47643700	-0.57352800	5.67806700
H	18.30447600	-0.58193200	4.97865800
C	17.80120500	-1.00668700	6.98122300
C	16.25439400	-0.12233600	5.13941200
C	19.24497900	-1.40461600	7.20388100
H	19.31708300	-2.43747200	7.56298300
H	19.71614900	-0.77029300	7.96377300
H	19.82712900	-1.32144300	6.28524800
C	16.28689000	0.28006800	3.67973500
H	17.29315200	0.19709900	3.26713700
H	15.94588400	1.31294000	3.54566400
H	15.62172100	-0.35321700	3.08097900
Mg	11.49524900	0.44077400	9.18236200
N	10.24181700	1.77298000	8.25039000
N	10.12700800	-0.14597600	10.58931400
C	8.34760000	1.22680400	9.67021400
H	7.29692000	1.45518000	9.80855600
C	8.96115100	1.90157000	8.59665100
N	13.84164700	2.13563800	10.74817100
C	8.87358800	0.28891700	10.58814500
C	13.50268900	1.23676800	9.78829600
C	8.07027600	2.83821600	7.80780300
H	8.04273000	2.55599100	6.74906800
H	7.04800200	2.83055600	8.18834900
H	8.44225600	3.86864200	7.85054200
C	12.99517700	2.84744600	11.61805200
C	13.50745700	3.26122600	12.85414600
C	7.90317300	-0.23295000	11.62657400
H	8.25906200	-0.01781500	12.64066200
H	6.91501200	0.21420600	11.51034600

H	7.79627200	-1.32127300	11.55196000
C	12.70932800	3.97462600	13.74316700
H	13.12331100	4.29013200	14.69696500
C	11.67716400	3.17308400	11.27692200
C	11.38750100	4.27513900	13.41729700
C	10.88106200	3.86876700	12.18361100
H	9.85705100	4.10491700	11.90835500
O	12.12605200	-1.06133500	8.06406000
N	12.78829200	-3.19899000	7.66111600
C	13.09018900	-1.87947500	7.92093500
C	13.80535300	-4.15402000	7.85056400
C	13.92050200	-5.21121500	6.93214300
C	14.89402600	-6.18496200	7.10616800
C	14.65752200	-4.11906200	8.96513600
C	15.75285600	-6.13869000	8.20938900
C	15.62543600	-5.10921000	9.13737300
H	14.98057000	-6.99256800	6.38440700
H	16.27351000	-5.07800400	10.00929000
H	13.47914300	-2.36682700	6.83173900
H	14.84330000	2.25774900	10.88757500
C	10.74056900	2.54827400	7.12553100
H	11.81523600	2.37182500	7.01367900
H	10.26857800	2.27262800	6.17084300
H	10.60862000	3.63253600	7.25466000
C	10.52985400	-1.11739200	11.59378100
H	9.96906900	-2.06055600	11.52588900
H	11.58681900	-1.36644000	11.45348400
H	10.42314000	-0.73975600	12.62092100
C	17.43044200	-1.51447700	9.29111200
H	18.24148700	-0.88083200	9.67792700
H	16.61561900	-1.46398300	10.02019000
H	17.79471700	-2.55179900	9.28327200
C	13.92659000	0.45128200	5.12607700
H	13.05709100	0.34319400	5.78165200
H	14.00162800	1.51515700	4.85534000
H	13.70125500	-0.10441800	4.20481100
H	16.50205400	-6.91250400	8.35001800
H	14.52055100	-3.34308500	9.71178300
H	13.23841100	-5.24529500	6.08747400
H	11.27664400	2.90116100	10.30536800
H	10.76266200	4.82745100	14.11298300
H	14.53248600	3.01236300	13.11953100

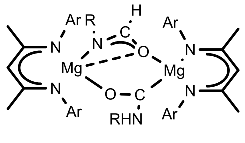

IRC-forward

Mg	15.54996200	0.50127500	7.42168800
N	17.28778400	-0.56539000	7.44929700
O	15.00753800	1.37528500	9.08926900
N	16.02270000	1.65300900	5.80905700

C	18.10384700	0.46428600	5.40608200
H	18.93393700	0.42407400	4.71027600
C	18.20065900	-0.43332300	6.48944500
C	17.12584000	1.43485000	5.09880000
C	19.45300500	-1.28283700	6.53440100
H	19.20751700	-2.35075000	6.55491900
H	20.03565400	-1.07449000	7.43941900
H	20.09209100	-1.09620200	5.67040600
C	17.39503000	2.27605800	3.86895700
H	18.31660100	1.97092200	3.37169200
H	17.48488400	3.33668200	4.13108200
H	16.57376900	2.19675500	3.14752400
Mg	12.29665800	-0.18434200	9.03352800
N	10.70789400	0.51480800	7.90251600
N	11.09281300	-0.66476600	10.65402400
C	9.03009900	0.04335500	9.60107900
H	7.96257800	0.10788600	9.77986700
C	9.44713600	0.49731000	8.33303700
N	13.80310700	2.28463100	10.66391700
C	9.78112300	-0.48961800	10.67541800
C	13.85904500	1.36315500	9.66654400
C	8.34397900	0.99820000	7.42265400
H	8.28408800	0.39574900	6.50869900
H	7.37221300	0.95918200	7.91697700
H	8.52670100	2.03191000	7.10749700
C	12.71526200	2.64962700	11.47877900
C	12.97930100	3.14614000	12.76168000
C	8.99460300	-0.85467600	11.91702500
H	9.31691100	-0.24544900	12.76963200
H	7.92418700	-0.69946600	11.77275800
H	9.15479000	-1.90220300	12.19598200
C	11.93520400	3.55164800	13.58761100
H	12.15689100	3.93839700	14.57858700
C	11.39268000	2.57676700	11.02857300
C	10.61472000	3.45584500	13.14976000
C	10.35350600	2.96488000	11.87096300
H	9.33227000	2.88904200	11.50902500
O	13.89321000	-0.60532200	7.25924800
N	12.92498000	-2.21460700	8.46408200
C	13.68379700	-1.86115800	7.47375000
C	12.66969100	-3.56983700	8.74207600
C	11.37108200	-3.94503300	9.11347500
C	11.08171500	-5.27471800	9.40361700
C	13.67464100	-4.54736900	8.69643900
C	12.07926300	-6.24844700	9.33724300
C	13.37562100	-5.87708300	8.98525000
H	10.06880800	-5.55265400	9.68313000
H	14.16495700	-6.62327700	8.94669800
H	14.13045800	-2.59701500	6.78864800
H	14.67891500	2.77741000	10.83179200

C	10.97041700	1.02012600	6.56275100
H	12.03261800	0.89703200	6.33715300
H	10.41581500	0.47912400	5.78203900
H	10.72864600	2.08833800	6.45194800
C	11.74664900	-1.19905700	11.83695500
H	11.40103700	-2.20925900	12.10025900
H	12.82368000	-1.27212800	11.65189300
H	11.61562300	-0.55944200	12.72152900
C	17.54274900	-1.49905400	8.53488400
H	18.45655100	-1.26199700	9.09853400
H	16.71321700	-1.46041800	9.24802300
H	17.63115200	-2.54191700	8.19481700
C	15.11903500	2.71362900	5.39062800
H	14.28767000	2.78210500	6.09901200
H	15.60047800	3.70169700	5.36805800
H	14.68273800	2.53656200	4.39673600
H	11.85016000	-7.28488300	9.56792000
H	14.69536200	-4.25844000	8.46020000
H	10.60305900	-3.17918700	9.16296300
H	11.17593800	2.22712600	10.02491800
H	9.79901200	3.76964900	13.79472700
H	14.00767700	3.20638600	13.11057900

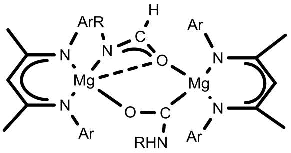

IRC-reverse

Mg	14.59606900	-0.64007400	7.65704800
N	16.03644800	-1.67649900	8.67841600
O	13.71921600	0.83373800	8.70875700
N	15.85536100	0.07614900	6.20983300
C	17.83767000	-0.82440500	7.28682500
H	18.91771800	-0.83990800	7.19586600
C	17.32864200	-1.55665000	8.37971000
C	17.17168800	-0.09933400	6.27467500
C	18.35755000	-2.25065000	9.24698200
H	19.37176200	-2.05624100	8.89581700
H	18.20156900	-3.33565000	9.25380300
H	18.28560300	-1.91487300	10.28790200
C	18.05489300	0.50098400	5.20140000
H	17.92320600	1.58754300	5.14195700
H	17.80132900	0.09705400	4.21440700
H	19.10913300	0.29548100	5.39117900
Mg	11.39165300	0.49517600	9.05144400
N	9.34583500	0.76736800	9.00130600
N	11.42630600	0.03578700	11.06348100
C	9.00080500	-0.05045900	11.25452200
H	8.21464500	-0.30469400	11.95643300
C	8.54864600	0.40831100	9.99768300
N	12.19396500	2.44401800	8.47137100
C	10.31041200	-0.19337000	11.75500500

C	13.43706000	2.06931700	8.46783300
C	7.04588500	0.48982100	9.82340300
H	6.72620700	1.52060600	9.63014800
H	6.71547600	-0.10876600	8.96667200
H	6.52138100	0.13367900	10.71131500
C	11.82627500	3.77539000	8.20553100
C	12.45931300	4.55151800	7.22306400
C	10.41088800	-0.63495600	13.20128300
H	10.90717000	0.12769300	13.81285200
H	9.42477000	-0.82064800	13.62920800
H	11.00411700	-1.55155700	13.29706800
C	12.05611000	5.86574200	6.99823700
H	12.55546100	6.45469900	6.23333400
C	10.76044400	4.32957600	8.92772900
C	11.00939200	6.41905500	7.73365800
C	10.36109500	5.64177100	8.69434600
H	9.53744300	6.06000200	9.26692200
O	11.61254000	-0.98104000	7.70586000
N	12.27507600	-2.56942600	6.34605800
C	12.62461100	-1.54829500	7.17681600
C	13.11207600	-3.39110000	5.57428500
C	14.44658600	-3.63944200	5.92076400
C	15.23384500	-4.45237400	5.10900100
H	16.26876100	-4.63007800	5.38839000
C	12.57937200	-3.99927600	4.42980800
C	14.70548500	-5.05145500	3.96631300
C	13.36962500	-4.82598500	3.63747400
H	12.93757000	-5.29037500	2.75521600
H	14.26172000	2.77612600	8.29559600
H	11.27199000	-2.69513600	6.22379900
C	15.30182800	0.80722600	5.08093800
H	15.51592000	0.32750600	4.11518400
H	14.21181700	0.85080700	5.17682500
H	15.66372100	1.84435600	5.01897500
C	15.65625300	-2.49023300	9.82371900
H	16.03658900	-2.09350900	10.77615500
H	14.56475500	-2.51774600	9.90198000
H	15.99748000	-3.53238700	9.74295800
C	12.69929600	-0.10635900	11.75529200
H	12.88429000	-1.13225100	12.10909800
H	13.50955900	0.15493300	11.07062000
H	12.78730000	0.55679800	12.62874400
C	8.74860000	1.23768200	7.76286600
H	8.08390800	2.10226800	7.90400200
H	9.54056200	1.55657300	7.07808100
H	8.16881100	0.45778900	7.24642800
H	15.32381400	-5.69311700	3.34566800
H	11.54274200	-3.81245100	4.15887100
H	14.86763600	-3.21580200	6.82679600
H	13.24880800	4.11498700	6.61717100

H	10.69339800	7.44219500	7.55160400
H	10.26081900	3.71472200	9.67047300

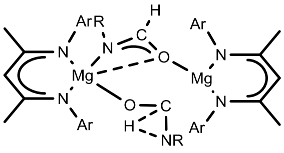

TS

Mg	14.77958700	-0.45856700	7.53853700
N	15.71787500	-1.76958700	8.79121400
O	14.11672400	1.24464000	8.27377500
N	16.36274000	-0.17938900	6.28066500
C	17.82491000	-1.61361100	7.58721100
H	18.84575400	-1.97668100	7.61855000
C	17.00164100	-2.08568400	8.63063200
C	17.54138300	-0.75126100	6.50581000
C	17.66091800	-3.02668800	9.61662000
H	18.71692800	-3.17112900	9.38498800
H	17.17369800	-4.00874200	9.61076800
H	17.58388500	-2.64250800	10.64003000
C	18.68937700	-0.48406700	5.55580500
H	18.91845400	0.58659200	5.50460400
H	18.43892300	-0.80420600	4.53779800
H	19.59360700	-1.00998000	5.86474100
Mg	11.35757800	0.52592500	9.12688200
N	9.31960400	0.46967600	9.28435800
N	11.70391700	0.44694400	11.14433200
C	9.35710200	0.10810700	11.68321900
H	8.69970700	-0.09113000	12.52188000
C	8.70536200	0.22336400	10.43530400
N	12.11668700	2.32082300	8.26927700
C	10.72028800	0.23638300	12.01787700
C	13.40717600	2.27948000	8.05740600
C	7.20076200	0.05584400	10.45556700
H	6.69834800	0.95921900	10.09064800
H	6.88929500	-0.76576200	9.80038600
H	6.83567100	-0.15358000	11.46198100
C	11.41513800	3.51140400	7.97131500
C	11.60718500	4.20569700	6.76822800
C	11.04900400	0.12960200	13.49242700
H	11.51185800	1.05262100	13.86058300
H	10.15458300	-0.06121500	14.08693200
H	11.76446000	-0.67911400	13.68103000
C	10.89262800	5.37306900	6.50634200
H	11.05312900	5.89956700	5.56918800
C	10.47190300	3.99283700	8.88990100
C	9.96600200	5.85453900	7.42887400
C	9.75659600	5.15528600	8.61820300
H	9.03288900	5.51863900	9.34299100
O	11.89924400	-0.94029900	7.91597200
N	12.18671400	-2.03405100	5.94625800
C	12.69067800	-1.27075100	6.97756800

C	13.10009000	-2.70764700	5.11236800
C	14.26337600	-3.31604900	5.60958100
C	15.12248800	-3.99129200	4.74164900
H	16.02036300	-4.45729300	5.13792300
C	12.79611900	-2.81886800	3.74489800
C	14.83003500	-4.07521400	3.38322700
C	13.66059200	-3.48755000	2.88919200
H	13.41957700	-3.56223500	1.83224100
H	13.91898700	3.19080100	7.70646900
H	12.72008600	-0.78048800	5.82566300
C	16.21271200	0.67911700	5.11714600
H	16.38118800	0.14913100	4.16883000
H	15.19014500	1.07089600	5.08493500
H	16.88512400	1.54909300	5.13399700
C	14.98534500	-2.35974200	9.90272100
H	15.36959400	-2.04155800	10.88258300
H	13.93722200	-2.05151400	9.85048400
H	14.99653300	-3.45897600	9.88760100
C	13.05564200	0.62943200	11.65358700
H	13.43296700	-0.24876400	12.19797700
H	13.73515100	0.81010900	10.81623000
H	13.14236400	1.49268100	12.32992900
C	8.52126500	0.57581700	8.07281300
H	7.77711700	1.38373000	8.12031400
H	9.17705100	0.79511200	7.22481000
H	7.98815000	-0.35480100	7.83132500
H	15.49654300	-4.61007400	2.71258300
H	11.87738700	-2.37094800	3.37715900
H	14.46972400	-3.28861500	6.67505600
H	12.29845700	3.81326400	6.02701600
H	9.40476500	6.76062700	7.21961700
H	10.31572300	3.44903300	9.81716300

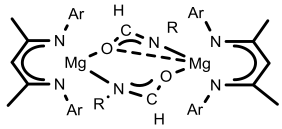

IRC-forward

Mg	15.81500200	0.40154600	8.07926400
N	17.24537200	-0.14096700	9.42198600
O	15.01146000	2.16220400	8.24841600
N	16.88729900	-0.07316300	6.41210900
C	18.70478400	-1.04247000	7.69998600
H	19.64404700	-1.56928300	7.57664200
C	18.35646800	-0.76527100	9.03983900
C	18.05836400	-0.70104700	6.49333600
C	19.35240300	-1.21398200	10.08782400
H	20.19213400	-1.74534400	9.63826000
H	18.88174200	-1.87556200	10.82391900
H	19.75074100	-0.35620200	10.64212400
C	18.78462500	-1.08266900	5.22130700
H	19.02834500	-0.19348000	4.62846100

H	18.16326800	-1.72522400	4.58696200
H	19.71414900	-1.61143900	5.43591100
Mg	12.03561100	0.17929200	8.39077300
N	10.03278800	0.45361300	7.93468600
N	11.64685300	-0.53542000	10.29146300
C	9.22937000	-0.39504000	10.05762100
H	8.31185900	-0.60273700	10.59650800
C	9.04729600	0.13826100	8.76127500
N	12.70388000	2.27154600	8.38841900
C	10.41023600	-0.69991800	10.76149900
C	13.91333800	2.78749300	8.36124600
C	7.61113200	0.36370200	8.33645900
H	7.43227500	1.42190100	8.11300000
H	7.37556000	-0.19884000	7.42560400
H	6.91194700	0.05615400	9.11548500
C	11.66039400	3.23546700	8.49281500
C	11.54369300	4.28525500	7.57148500
C	10.21333300	-1.25243000	12.15900600
H	10.71674800	-0.63000700	12.90771400
H	9.15523800	-1.30728900	12.41872200
H	10.63721500	-2.25947500	12.24893800
C	10.51943900	5.22399400	7.69171400
H	10.44704800	6.03358100	6.97003500
C	10.71678700	3.13148600	9.52352000
C	9.58921200	5.11970100	8.72334200
C	9.69209000	4.06694200	9.63461200
H	8.97080800	3.97358600	10.44220700
O	14.08253000	-0.61041200	8.10387100
N	12.43857200	-1.48973600	6.89197300
C	13.67698200	-1.51080200	7.25798400
C	11.91798600	-2.50398100	6.06860300
C	12.21277100	-3.85905300	6.28161000
C	11.67829700	-4.83582900	5.44429900
H	11.91416900	-5.88137300	5.62418600
C	11.05425200	-2.15470800	5.02201300
C	10.83421100	-4.48035500	4.39392900
C	10.52268300	-3.13539600	4.19071600
H	9.85961400	-2.84734800	3.37922000
H	13.99256200	3.88297300	8.46058000
H	14.40442800	-2.24172400	6.87877400
C	16.36360400	0.26554900	5.09698100
H	16.18092600	-0.61580500	4.46501300
H	15.40610800	0.78361700	5.21088600
H	17.02562900	0.93997000	4.53525600
C	17.04240600	0.12436200	10.83850600
H	17.84497500	0.73298100	11.27832500
H	16.11127300	0.68354600	10.97199200
H	16.95801900	-0.79337000	11.43869800
C	12.75963100	-0.88278200	11.16184800
H	12.72508700	-1.92469900	11.51303500

H	13.69517100	-0.76745400	10.60941300
H	12.82270000	-0.24157600	12.05461400
C	9.69533500	1.04663700	6.65224500
H	9.21020600	2.02791400	6.75427600
H	10.61197300	1.20814800	6.07453000
H	9.03715700	0.41117500	6.04202700
H	10.41341800	-5.24437200	3.74667400
H	10.81719700	-1.10693200	4.86819900
H	12.83709700	-4.14476100	7.12403900
H	12.25146100	4.35143700	6.74902500
H	8.78874100	5.84841700	8.81555400
H	10.79186400	2.30994500	10.22986700

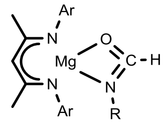

IRC-reverse

O	7.11788100	3.38654600	4.60650100
N	6.35066700	6.65679900	2.41807400
N	4.80757300	4.10187800	2.61293100
C	5.10509100	8.42143300	1.23580000
H	5.06597600	9.13022900	2.07113400
H	4.19147700	8.53973500	0.65134000
H	5.95477900	8.71997600	0.61142700
C	5.25667800	7.00097800	1.74121100
C	4.18029300	6.13554400	1.45755300
H	3.37246400	6.57917000	0.88639000
C	3.93816900	4.82144800	1.91582400
C	2.57855500	4.24419200	1.57906000
H	2.66698800	3.28257200	1.06071600
H	2.00215800	4.92291900	0.94856400
H	1.99969100	4.05578300	2.49076300
C	8.27673400	3.86475200	4.82438400
H	8.91438100	3.39868400	5.59309000
C	9.94477100	5.47534200	4.36759400
C	10.69979100	5.97367100	3.29308600
C	11.93409700	6.57885300	3.50662000
H	12.50133900	6.95167600	2.65749400
C	12.44611800	6.70519100	4.79809700
H	13.40945600	7.17849700	4.96431900
C	11.69794600	6.23009700	5.87435200
H	12.07409000	6.33960500	6.88841600
C	10.45732200	5.63265600	5.66776500
C	4.39671300	2.78293800	3.07832600
H	5.19262500	2.36128600	3.69441300
H	3.48748400	2.81511700	3.69494300
H	4.20131200	2.08238500	2.25132500
C	7.33202500	7.69726700	2.69709600
H	6.91143500	8.53373400	3.27350900
H	8.14693900	7.27376700	3.28598100
H	7.77602100	8.12321700	1.78461000

H	10.30312800	5.86883000	2.28711200
H	9.86564700	5.30978800	6.51998800
N	8.70290500	4.88272800	4.10760600
C	7.90250800	3.66557200	-1.04389300
C	6.85009100	3.92929700	0.04082400
B	8.80621500	3.19651500	0.97444700
O	8.98927100	3.02446200	-0.35823900
O	7.60428400	3.81732100	1.27681800
H	7.53783600	3.00690800	-1.83600600
H	8.27578000	4.58971700	-1.49889400
H	6.06219900	3.17224800	0.06247200
H	6.39034400	4.91813000	-0.00406200
Mg	6.74278100	4.72433500	3.06060800
H	9.57954800	2.85577400	1.80268100

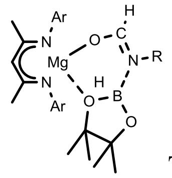

TS

O	7.09193400	3.78992500	4.48874200
N	5.98837700	6.50796000	2.33082000
N	4.38939600	3.94917200	2.55166900
C	4.62679000	8.37836600	1.49638500
H	4.79607600	9.02784100	2.36286900
H	3.62194700	8.57178700	1.11891900
H	5.34621500	8.68559800	0.72853100
C	4.80591500	6.92170800	1.86911300
C	3.67307300	6.10287800	1.69834400
H	2.80590200	6.60696500	1.28733200
C	3.46293800	4.74619000	2.03464300
C	2.06937000	4.20916500	1.78884700
H	2.09434500	3.32638100	1.13998000
H	1.42853200	4.95871700	1.32265500
H	1.59937800	3.89699700	2.72876000
C	8.30720600	4.18352200	4.60753400
H	8.78498600	4.03958200	5.59135900
C	10.23367500	5.28231400	3.90027500
C	11.31532500	5.04903900	3.03478500
C	12.55720000	5.62615200	3.28073100
H	13.38266300	5.42401600	2.60252200
C	12.75243700	6.45285500	4.38803000
H	13.72316300	6.90353000	4.57418700
C	11.68325200	6.70065800	5.24660400
H	11.81460600	7.35221600	6.10700300
C	10.43561900	6.12976600	5.00428000
C	4.02993000	2.58181000	2.89856300
H	4.89107500	2.08937900	3.35944600
H	3.20559200	2.52855700	3.62333100
H	3.73851900	1.98311900	2.02302300
C	7.04529000	7.49540500	2.51665100

H	6.78178500	8.26121900	3.26027100
H	7.94563200	6.98948200	2.87376900
H	7.30946200	8.02407100	1.58867700
H	11.17084800	4.39360300	2.18166200
H	9.59789500	6.35780500	5.65850700
N	8.97662600	4.71683100	3.61905300
C	9.10859800	4.46006400	-0.13796100
C	7.58605000	4.42168700	0.02551100
B	8.61384100	3.04975100	1.55510300
O	9.58569000	3.36831100	0.65772000
O	7.39487000	3.68139300	1.25704000
H	9.42820900	4.31957600	-1.17354500
H	9.54094700	5.38906000	0.24981700
H	7.08694600	3.86849000	-0.77610900
H	7.12716000	5.40679100	0.13108200
Mg	6.31484500	4.55630100	2.84949400
H	8.69769300	2.18272200	2.35507000

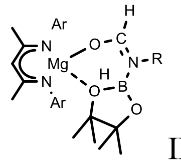

IRC-forward

O	7.17346800	5.25742800	4.22789300
N	5.13094900	7.02473500	2.12266400
N	4.50159300	4.06775700	2.34290600
C	3.14261200	8.36988000	1.59688900
H	3.26949300	9.06166700	2.43751900
H	2.07392200	8.23978200	1.42227200
H	3.57470100	8.85919500	0.71620700
C	3.82284200	7.04639400	1.87371400
C	2.99274600	5.90779500	1.84450200
H	1.95142600	6.11229200	1.62392200
C	3.29277400	4.54486200	2.05740400
C	2.12618600	3.58713200	1.94474700
H	2.30281900	2.84178000	1.16076500
H	1.19948400	4.11300100	1.71185600
H	1.98007800	3.03369000	2.87939000
C	8.42143700	5.18902700	4.39510200
H	8.83628200	5.51555000	5.35912500
C	10.66964400	4.79871800	3.80309100
C	11.49210900	3.68351100	3.60180900
C	12.84509200	3.75475600	3.91727600
H	13.47426900	2.88314700	3.75930600
C	13.39468300	4.92694500	4.43835900
H	14.45162800	4.97354000	4.68448300
C	12.57993100	6.03988600	4.62995300
H	12.99771100	6.96396100	5.02008700
C	11.22624300	5.98227600	4.30316100
C	4.65909000	2.63313600	2.54289300
H	5.70999900	2.40951400	2.74684700
H	4.07482700	2.25752700	3.39512200

H	4.36994900	2.04605900	1.65981000
C	5.86398200	8.28141600	2.12400500
H	5.51781800	8.97870100	2.90070300
H	6.92188000	8.08229300	2.32268200
H	5.81137000	8.81064800	1.16152000
H	11.06618400	2.77427400	3.19489300
H	10.60092500	6.86419400	4.41344700
N	9.28242000	4.72976200	3.50062900
C	9.78049800	4.81027400	0.32982000
C	8.33181600	5.28496500	0.20749700
B	8.70009400	4.00287800	2.18086300
O	9.71221700	3.71573300	1.21524700
O	7.79937500	5.02614500	1.50535000
H	10.20157400	4.49040500	-0.62965700
H	10.41593200	5.61588600	0.73440800
H	7.78154200	4.70102000	-0.54196600
H	8.23800100	6.34977300	-0.03105700
Mg	6.11179800	5.29284800	2.53804200
H	8.06773600	3.03311500	2.55883200

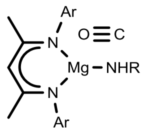

IRC-reverse

O	11.33386200	-1.84392300	8.37696500
Mg	11.11109400	0.83824700	9.29023600
N	9.33707900	0.73573200	8.32946400
N	10.50718100	-0.01695700	11.01962600
C	8.38522000	-0.69101200	10.04760400
H	7.49963400	-1.27463500	10.26996400
C	8.34614100	-0.01195300	8.80976900
C	9.34412700	-0.65922000	11.08614400
C	7.07445100	-0.16436100	8.00543400
H	7.28246100	-0.56251200	7.00585400
H	6.36674000	-0.83108900	8.49930900
H	6.58538500	0.80627600	7.86361000
C	8.98252900	-1.41265900	12.34671200
H	8.89960600	-0.72779400	13.19850400
H	8.03196900	-1.93648700	12.24003400
H	9.75367400	-2.14682500	12.60517900
C	10.60548200	-2.70519000	8.54488500
N	12.80222000	1.66802800	8.75153100
C	13.48768900	2.63834600	9.44577400
C	14.80471100	3.03448000	9.11481900
C	12.89171600	3.29674700	10.54857600
C	15.47475700	4.01066500	9.84279600
H	15.29874900	2.55551700	8.27064500
C	13.56988600	4.26846000	11.27417900
H	11.86590300	3.04697300	10.82826900
C	14.87179600	4.63925800	10.93354000
H	16.48742600	4.28287200	9.55273900

H	13.07049200	4.74807200	12.11343900
H	15.39918400	5.40116700	11.49931700
C	9.15534400	1.42515600	7.05891400
H	10.05572900	2.00557200	6.83647300
H	8.99410700	0.73518900	6.21840800
H	8.31107400	2.12779800	7.07588100
C	11.39448700	-0.03156300	12.17633200
H	11.77365700	-1.03805500	12.40385500
H	12.25975600	0.60634800	11.97502600
H	10.91177700	0.35271600	13.08470400
H	13.35599000	1.33006300	7.97254600

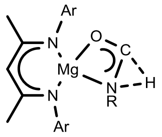

TS

O	12.77892500	-0.97987800	8.64687100
Mg	11.10642600	-0.02113400	9.29848400
N	9.44364800	0.18699000	8.16093800
N	10.16588600	-0.30931700	11.06778100
C	7.96277000	-0.06233800	10.07213800
H	6.91085500	-0.06622000	10.33282800
C	8.22686500	0.13998600	8.70170900
C	8.83740700	-0.27512800	11.15814000
C	7.01520100	0.30832300	7.81125400
H	6.98275900	-0.47093000	7.04109600
H	6.08884500	0.25785300	8.38455500
H	7.04123600	1.27049300	7.28700400
C	8.18279700	-0.48081700	12.50655200
H	8.52403500	0.26936500	13.22898000
H	7.09637300	-0.41685500	12.43650900
H	8.44073600	-1.46155600	12.92238300
C	13.56307400	0.01415400	8.66565000
N	12.86876300	1.21898400	9.07520200
C	13.58975900	2.28242600	9.68056300
C	13.10686800	3.59090600	9.54020900
C	14.76218400	2.05235300	10.41267800
C	13.79302800	4.65348700	10.11643600
H	12.19405300	3.75847800	8.97510400
C	15.44835600	3.12593200	10.97805700
H	15.11744700	1.03479300	10.53742300
C	14.96850400	4.42607500	10.83590900
H	13.40973100	5.66407200	10.00477900
H	16.35825000	2.94017200	11.54237200
H	15.50004800	5.25758600	11.28969900
C	9.56607900	0.38939100	6.72443400
H	10.62510300	0.39480500	6.44825900
H	9.08790700	-0.40846400	6.13932100
H	9.13917400	1.34562800	6.39016400
C	10.94625100	-0.55106500	12.27337500
H	10.71428600	-1.51623100	12.74467200

H	12.01045700	-0.56788100	12.01903000
H	10.80922800	0.23065400	13.03379600
H	13.48916600	1.10926100	7.96419900

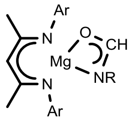

IRC-forward

O	12.72550000	-1.04041200	8.82904800
Mg	11.07496200	-0.00580000	9.47872700
N	9.47750100	0.36115700	8.28895000
N	10.03202100	-0.43480100	11.16055900
C	7.88784000	-0.14040800	10.05701600
H	6.82235000	-0.19842700	10.24670300
C	8.23126100	0.22918100	8.74007100
C	8.70075400	-0.44035500	11.17030200
C	7.07351000	0.48726300	7.80059700
H	7.11566000	-0.17947300	6.93173300
H	6.11487000	0.33718400	8.29854500
H	7.10048000	1.51261900	7.41412200
C	7.97175800	-0.78569100	12.45042500
H	8.22155700	-0.07654300	13.24807100
H	6.89033600	-0.77125000	12.31005900
H	8.25733500	-1.78060100	12.81047500
C	13.44850600	-0.00420000	9.00411300
N	12.89448100	1.09564700	9.47481400
C	13.63600100	2.25193000	9.75116900
C	13.02574400	3.50177700	9.56604300
C	14.95190100	2.21387200	10.24158700
C	13.71874000	4.67722400	9.83543100
H	12.00352000	3.53277200	9.19869800
C	15.64302600	3.39491400	10.50046400
H	15.42363700	1.25682000	10.44687500
C	15.03424500	4.63240600	10.29803600
H	13.22818600	5.63485400	9.68159700
H	16.66068900	3.34470800	10.87907100
H	15.57404100	5.55082600	10.50955500
C	9.68637600	0.75124100	6.90193200
H	10.76017600	0.83284900	6.70751700
H	9.28504300	0.01827200	6.18774700
H	9.23999500	1.72616400	6.65992600
C	10.74669900	-0.75538100	12.38788100
H	10.55735700	-1.78096700	12.73534900
H	11.82321200	-0.66858800	12.21168300
H	10.50121500	-0.07487100	13.21512400
H	14.51569000	-0.04208400	8.73616500

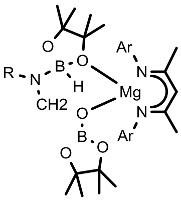

IRC-reverse

N	9.58404000	0.71538900	9.35105200
N	10.47658100	-1.29230400	11.43770100
C	8.21633300	-0.63445000	10.83640600
H	7.18053600	-0.83536700	11.08518000
C	8.40265600	0.32171200	9.81403300
C	9.15554600	-1.38143400	11.57594400
C	7.13916000	0.91606500	9.22825100
H	7.11644900	2.00406300	9.35981400
H	7.07970000	0.72639900	8.15035400
H	6.24713500	0.49839900	9.69691700
C	8.57915900	-2.34617800	12.59111600
H	8.93649900	-2.11852900	13.60224000
H	7.48899000	-2.31090400	12.60056800
H	8.88486900	-3.37584100	12.37214700
C	9.62364500	1.70970400	8.28976200
H	10.66569900	1.93914000	8.04689300
H	9.14498000	1.36342900	7.36215500
H	9.14588300	2.65859500	8.57337800
C	11.32200400	-2.14338900	12.25975600
H	11.16078700	-3.21556000	12.07377200
H	12.37307900	-1.93585700	12.03539900
H	11.18539300	-1.97563000	13.33849000
Mg	11.34131600	-0.07083600	10.04155500
O	12.43296100	1.34324100	11.04845600
O	14.03695300	2.95593700	11.57959900
O	12.69743700	-0.73392400	8.82899300
O	11.80135800	-2.60410700	7.57224900
O	14.05005200	-2.10912800	7.40281700
N	14.70101700	1.04714400	10.05519000
C	12.35568800	1.60260200	12.45435200
C	13.05762200	2.95225300	12.59386100
C	14.64978200	0.63368000	8.83739400
H	15.24352700	-0.20478100	8.48631000
H	14.07036500	1.21165600	8.12866400
C	12.38401500	-3.64920300	6.81089900
C	13.79589800	-3.13812300	6.45815900
C	15.43873300	0.29455900	11.02253300
C	15.38959900	-1.10336700	11.01136900
C	16.12991200	-1.82340400	11.94642800
H	16.09107300	-2.90911800	11.93813000
C	16.90998200	-1.15643100	12.89006500
C	16.94392000	0.23809500	12.90060100
H	17.55091700	0.76192700	13.63367400
C	16.20676800	0.97136800	11.97403800
B	13.57646600	2.14334700	10.49696300
B	12.82416500	-1.75000700	7.98712500
H	17.48672400	-1.72107700	13.61716100
H	16.21204500	2.05470600	11.97440800
H	14.76002600	-1.60733600	10.28419800
H	12.88594100	0.81653600	13.00764100

H	11.30763400	1.61445800	12.76788700
H	12.34287400	3.77788900	12.44935000
H	13.52913000	3.07600200	13.57578800
H	11.77525900	-3.85541600	5.92375200
H	12.42475200	-4.56637200	7.41558500
H	14.56088100	-3.91903000	6.53387500
H	13.83470400	-2.72173700	5.44158600
H	13.26874000	2.69701100	9.45752200

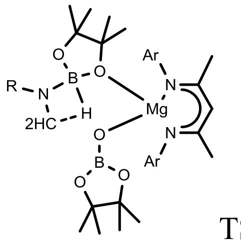

TS

N	9.76419900	0.91829600	9.09738500
N	9.26970800	-1.26120100	11.11948900
C	7.71686300	0.41280900	10.29680700
H	6.68736700	0.73863900	10.39542400
C	8.48770800	1.15200200	9.37274200
C	8.05936000	-0.71430500	11.07156300
C	7.76327400	2.27558300	8.65969200
H	8.25054700	3.24024600	8.84405500
H	7.76935300	2.12028800	7.57453200
H	6.72491400	2.35150800	8.98582200
C	6.93392700	-1.32404800	11.88269800
H	7.19144300	-1.37315900	12.94722500
H	6.01210600	-0.74944100	11.78081000
H	6.73019600	-2.35178100	11.55933400
C	10.42759800	1.74399900	8.10352300
H	11.47589400	1.43795100	8.02383500
H	9.98804300	1.64437200	7.09968200
H	10.42488600	2.81429600	8.35894000
C	9.45698000	-2.47021900	11.90206800
H	8.81342100	-3.29964400	11.57375400
H	10.49011800	-2.81406800	11.78974400
H	9.26875600	-2.32474000	12.97868000
Mg	10.88430500	-0.51983200	10.06813900
O	13.52442500	-1.61785700	12.71211000
O	13.75397300	0.57592100	13.38834400
O	11.91759100	-1.83550600	9.11975600
O	11.73096500	-3.23497700	7.14580700
O	13.78457000	-2.32517500	7.68928200
N	14.86634000	-0.11485100	11.24903900
C	12.82645800	-1.51708000	13.96396200
C	12.77821400	-0.00447200	14.27024500
C	14.32399000	-0.50071600	10.12356100
H	14.75614500	-0.15094600	9.18799200
H	13.50612700	-1.22233400	10.06604200
C	12.66929100	-3.80285100	6.25406600

C	13.94786100	-2.95885000	6.43379000
C	16.05415200	0.68319800	11.27108800
C	17.11368900	0.32402300	10.43259200
C	18.26756300	1.10222900	10.40997300
H	19.09072100	0.81943600	9.76068500
C	18.37178800	2.22359700	11.23103100
C	17.31645300	2.56387300	12.07669100
H	17.39247900	3.43779900	12.71694000
C	16.15331000	1.79939400	12.10586000
B	13.96862100	-0.36493900	12.43092500
B	12.41875100	-2.42660600	8.05811700
H	19.27532300	2.82586400	11.21777300
H	15.32535800	2.06811900	12.75088400
H	17.04391400	-0.57510800	9.82684700
H	13.38711400	-2.08352500	14.71541000
H	11.83152700	-1.95426500	13.85626700
H	11.80309500	0.43029500	14.03066700
H	13.04383500	0.23625300	15.30282000
H	12.28658200	-3.77288700	5.22697500
H	12.84296000	-4.85691900	6.51766900
H	14.85998800	-3.56824200	6.42658000
H	14.04399200	-2.19929300	5.64301700
H	12.29515600	0.28188200	11.01287600

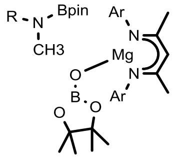

IRC-forward

N	10.77644300	0.42297700	9.73393400
N	9.26289500	-1.75268600	11.18273000
C	8.83247700	0.63577200	11.17770900
H	8.13369600	1.37495500	11.55340400
C	9.82183100	1.14891800	10.30260200
C	8.51145500	-0.69969800	11.50060500
C	9.72414200	2.62738800	9.99242800
H	10.68491100	3.13084900	10.14455000
H	9.44625900	2.78526600	8.94367600
H	8.97366600	3.11679900	10.61490900
C	7.20865500	-0.91211900	12.24162900
H	7.35524600	-1.50267900	13.15314900
H	6.74567100	0.03696400	12.51536500
H	6.49713400	-1.46714400	11.61898400
C	11.64932900	1.04876300	8.74641500
H	12.25486800	0.27610800	8.26461600
H	11.08856200	1.55881400	7.95160100
H	12.33411200	1.78382200	9.19282800
C	8.76190300	-3.09272700	11.45864900
H	7.79058000	-3.28929500	10.98436100
H	9.46892600	-3.82537000	11.05924300
H	8.64562000	-3.29679900	12.53428200
Mg	11.08488400	-1.52159200	10.28614300

O	12.40952800	-1.21975600	12.01113500
O	13.11004900	0.70114800	13.07277300
O	11.85611400	-2.94230200	9.33754200
O	12.61308900	-4.41485600	7.57894200
O	12.95447300	-2.14323300	7.34194400
N	14.42938000	-0.10785900	11.11206600
C	11.74912900	-1.17115800	13.30006300
C	11.89338400	0.29153600	13.70425700
C	14.67155300	-1.27645000	10.26077200
H	15.72959200	-1.55626600	10.31049100
H	14.07285800	-2.11649900	10.61021200
C	13.39724800	-4.24582200	6.41335400
C	13.33748600	-2.73492000	6.11181800
C	15.29207800	1.00392000	10.92805700
C	16.08703200	1.09312700	9.77379600
C	16.94578000	2.17319900	9.57989400
H	17.54551700	2.21251100	8.67438200
C	17.03683500	3.18968600	10.52495400
C	16.25132300	3.10609200	11.67376800
H	16.30998100	3.88402300	12.43070200
C	15.39279500	2.03207100	11.88182200
B	13.36361200	-0.16803100	12.03814400
B	12.42617500	-3.16149800	8.17021900
H	17.70821900	4.02959100	10.37286000
H	14.80302900	1.98703600	12.78781300
H	16.03515900	0.32659100	9.01010200
H	12.28209900	-1.84656300	13.97858200
H	10.71565100	-1.49784100	13.17946800
H	11.06156000	0.89452400	13.32237300
H	11.97993500	0.43145100	14.78500600
H	12.99777600	-4.85462400	5.59385900
H	14.42808000	-4.57768200	6.60777500
H	14.29978100	-2.33080400	5.77616700
H	12.58711000	-2.50546500	5.34123100
H	14.40130100	-1.09826900	9.21509400

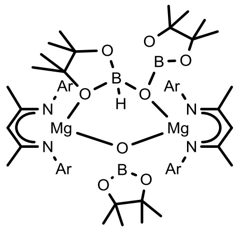

IRC-reverse

O	0.40797900	14.27176200	18.70340200
N	2.66431400	16.67669200	17.70459200
N	1.50708800	17.17354000	20.46392900
C	4.92568400	17.60358400	17.45752700
H	5.36761500	16.67199600	17.08573600
H	5.67884400	18.12565100	18.04925700
H	4.69755500	18.21774700	16.57860500
C	3.67713700	17.32829500	18.26872400
C	3.68803200	17.80367000	19.59737100

H	4.60305500	18.30528000	19.89109300
C	2.70193900	17.74063100	20.60478800
C	3.06741600	18.38142100	21.92726300
H	2.37321400	19.19165600	22.17898500
H	4.07666000	18.79473000	21.90445300
H	3.01439700	17.65319400	22.74474700
B	1.34382900	13.36962500	19.05571600
O	1.37874400	12.06158800	18.60120600
O	2.40066100	13.65935800	19.90885600
C	3.26826500	12.53040800	19.91244700
C	2.42556200	11.39401600	19.29658500
Mg	-1.37898000	13.56755800	18.04588800
O	-2.47745100	15.30512700	18.13947600
N	-2.11657300	12.31018500	19.47971800
N	-1.68081300	12.29806100	16.46953800
C	-3.27392400	10.29320900	20.27040900
H	-4.04938100	10.84332200	20.81577200
H	-3.71984200	9.38181300	19.86961600
H	-2.51285500	10.00489700	21.00512000
C	-2.67444700	11.14559200	19.17156300
C	-2.75098200	10.61366500	17.86720200
H	-3.23513500	9.64593800	17.80090200
C	-2.28366200	11.12276100	16.63735900
C	-2.50512300	10.22746500	15.43529500
H	-1.55369100	9.96694100	14.95757100
H	-3.00825700	9.30144500	15.71688800
H	-3.11423300	10.72983900	14.67505200
B	-3.55772900	15.74726300	18.83865900
O	-3.78938800	17.06252600	19.18122400
O	-4.51669900	14.88293000	19.31781300
C	-5.54223200	15.66920100	19.91567200
C	-4.94928600	17.09383300	20.00808200
H	-4.65320900	17.35282600	21.03251500
H	-5.63928200	17.86256700	19.64580500
H	-6.43575400	15.63531300	19.28043800
H	-5.80211900	15.25511100	20.89522700
H	3.59287000	12.31372300	20.93511200
H	4.15722400	12.75578900	19.30894100
H	1.99076600	10.74305300	20.06616000
H	2.99564500	10.77042500	18.60069500
C	-2.09896000	12.73207800	20.86966300
H	-3.10562900	12.92443700	21.26783600
H	-1.61221600	12.00500000	21.53640400
H	-1.53397500	13.66606100	20.95421500
C	-1.22855200	12.65297700	15.13236300
H	-0.46914100	11.95829800	14.74416200
H	-2.04694400	12.69172200	14.39912000
H	-0.77823600	13.64869500	15.15556700
C	2.78576800	16.25510400	16.31743000
H	2.95883600	17.09343100	15.62722200

H	3.59745200	15.52922400	16.16005600
H	1.85592600	15.77058600	16.00421300
C	0.58964400	17.18371000	21.59184000
H	0.96662100	16.61391200	22.45396300
H	0.35221900	18.19743300	21.94578200
H	-0.35726800	16.72361400	21.29167400
C	-1.09969300	18.36667800	17.85449200
C	-1.87719400	18.35915300	16.53691900
O	-0.67748200	17.00377700	17.98018400
O	-2.43524100	17.06970700	16.44655500
H	-0.22544600	19.02473400	17.84787100
H	-1.75078000	18.62214500	18.69726300
H	-2.67015100	19.11609100	16.52495400
H	-1.20328300	18.55523200	15.68663300
Mg	0.98340100	16.19113300	18.75454300
B	-1.64027200	16.15817600	17.20101600
H	-0.98918100	15.32481700	16.56072900

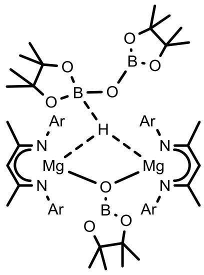

TS

O	0.97296900	13.74180100	19.19311800
N	2.98706700	16.15044600	17.51884600
N	1.78896400	17.02444700	20.15463500
C	4.91298600	17.52950300	16.86283700
H	5.61793900	16.69470100	16.77461800
H	5.46483900	18.40393200	17.21052600
H	4.54196300	17.73839600	15.85278800
C	3.77840000	17.18202700	17.80422200
C	3.65270500	18.00162700	18.94453300
H	4.38122400	18.80166100	19.01074900
C	2.75805800	17.92482800	20.03452100
C	2.95674800	18.95363700	21.12760000
H	2.04001600	19.52795900	21.30258300
H	3.75647800	19.65211800	20.87715300
H	3.21298600	18.46988400	22.07738900
B	1.75281300	12.80054300	19.74475300
O	1.43269800	11.45375400	19.83234200
O	3.00250500	13.07752600	20.29206300
C	3.60730400	11.83874400	20.63751900
C	2.45404800	10.81365400	20.58485500
Mg	-0.87693200	13.77944200	18.34244100
O	-2.73417300	15.66012200	18.81931000
N	-1.99493400	12.49709300	19.47649300
N	-1.43537700	12.96692500	16.54927100
C	-3.59502700	10.67518900	19.85457300

H	-4.23002700	11.26222400	20.52741700
H	-4.23142700	9.99795900	19.28259600
H	-2.93545500	10.06867100	20.48618600
C	-2.79073800	11.58010300	18.94495800
C	-2.95041100	11.35894200	17.55810400
H	-3.64618000	10.56829700	17.30014100
C	-2.32553500	11.98042500	16.45961900
C	-2.71890600	11.45930300	15.09284700
H	-1.84322500	11.09630300	14.54255500
H	-3.43584700	10.64048100	15.17024900
H	-3.16956800	12.25130100	14.48320100
B	-4.03074800	15.59401100	19.23292800
O	-4.96395900	16.55944100	18.93141600
O	-4.51851400	14.57003900	20.00448800
C	-5.92585000	14.77672500	20.12425400
C	-6.15436400	16.22574200	19.64333300
H	-6.27849300	16.92436500	20.48004600
H	-7.01911800	16.32207400	18.98014700
H	-6.44734900	14.04673300	19.49354000
H	-6.23264300	14.62050100	21.16284500
H	4.06688300	11.90943200	21.62908000
H	4.39703100	11.60391200	19.91099000
H	2.07306100	10.57628500	21.58747500
H	2.74307400	9.87516900	20.09993200
C	-1.90740800	12.59156100	20.92454600
H	-2.85076700	12.92469700	21.37804700
H	-1.61387600	11.64414500	21.39712600
H	-1.13987600	13.32549300	21.19015700
C	-0.87031100	13.50937500	15.32465200
H	-0.25913500	12.77664700	14.77535400
H	-1.63238800	13.89001000	14.62967600
H	-0.22399600	14.35537500	15.57734600
C	3.25514800	15.37481100	16.31831800
H	3.19257800	15.97101900	15.39597600
H	4.24412400	14.89393100	16.33224300
H	2.51331200	14.57461100	16.23151600
C	0.95233100	17.04671400	21.34466600
H	1.52945100	16.92988800	22.27307200
H	0.35776200	17.96665300	21.43044900
H	0.24217700	16.21559600	21.30156000
C	-1.26354500	18.79866500	17.92003100
C	-1.54990900	18.15320900	16.54367900
O	-1.60337000	17.79213000	18.87549500
O	-2.32029400	16.98896000	16.83533500
H	-0.21237000	19.07524300	18.04297900
H	-1.88523800	19.68310400	18.10310800
H	-2.11790300	18.80804000	15.87594300
H	-0.62609500	17.84877600	16.03810600
Mg	1.42289900	15.61824900	18.72089800
B	-2.19196300	16.76669400	18.17978400

H	-0.25902100	15.54789800	17.88746800

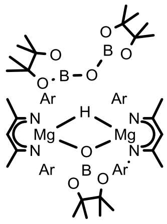

IRC-forward

O	1.55633800	13.50709100	18.85549000
N	3.26367000	16.21762500	17.35281700
N	2.08928900	16.79466000	20.08114100
C	4.98257400	17.85028900	16.70723800
H	5.77469400	17.11164400	16.53821100
H	5.44565100	18.76267600	17.08534600
H	4.54098300	18.07075300	15.72855600
C	3.94181800	17.31864700	17.66947300
C	3.77271100	18.05175700	18.86208000
H	4.40340800	18.92912800	18.94883600
C	2.94515200	17.80733000	19.97942300
C	3.07818300	18.78474000	21.12808200
H	2.10786700	19.21864200	21.39408900
H	3.76269700	19.59775200	20.88227700
H	3.45672600	18.28214000	22.02599100
B	2.34035500	12.55513400	19.38635700
O	1.99163700	11.21469800	19.45522500
O	3.59380000	12.80336000	19.92805900
C	4.17351000	11.54829200	20.26350300
C	3.00094700	10.54533600	20.20118700
Mg	-0.30888200	13.49504700	18.11315200
O	-3.94810200	16.55655800	20.03496700
N	-1.86220800	12.92701900	19.29890900
N	-0.86884400	12.41264000	16.48090100
C	-3.97979400	11.78137900	19.77423200
H	-4.49526400	12.68305600	20.12376200
H	-4.70541900	11.13766100	19.27439300
H	-3.61706200	11.25540400	20.66523300
C	-2.83327000	12.13733000	18.85277400
C	-2.88624600	11.58407900	17.55381600
H	-3.75255400	10.96084800	17.36342200
C	-1.99151900	11.69466600	16.47157500
C	-2.36694200	10.92620700	15.22207100
H	-1.58552900	10.20621100	14.95279700
H	-3.30093700	10.37905600	15.35721600
H	-2.48729500	11.59929500	14.36524200
B	-5.23975200	16.19491000	19.83161900
O	-6.14523900	16.90471800	19.08295600
O	-5.76884700	15.07380300	20.43001700
C	-7.11244700	14.94490600	19.96862500
C	-7.41500400	16.27769700	19.24463600
H	-8.06365500	16.93277500	19.83891900
H	-7.87908800	16.13028900	18.26454800

H	-7.17626800	14.08424200	19.29228500
H	-7.77694100	14.76501700	20.81952700
H	4.63263200	11.60231400	21.25604300
H	4.95926400	11.30575200	19.53568400
H	2.61399000	10.30552200	21.20042600
H	3.27137900	9.60712900	19.70567600
C	-1.92918800	13.40894100	20.67145000
H	-2.80891500	14.03680700	20.86101500
H	-1.93045300	12.59267300	21.40811400
H	-1.04686000	14.02232200	20.87961300
C	-0.03867900	12.42500000	15.28791900
H	0.36736200	11.43376200	15.03758600
H	-0.56580600	12.79990700	14.39853300
H	0.81776300	13.08748500	15.45084800
C	3.56460500	15.54838400	16.09744400
H	3.36975200	16.17771100	15.21667600
H	4.60737900	15.20533700	16.03409500
H	2.93262100	14.65961000	16.00175900
C	1.31713600	16.64870800	21.30714700
H	1.94658200	16.60368300	22.20650900
H	0.58249800	17.45358300	21.44667300
H	0.75372700	15.71165700	21.26733200
C	-1.31977000	18.52866400	18.67695700
C	-1.99616300	17.97036100	17.40396800
O	-1.99261700	17.89024400	19.76506900
O	-3.17374200	17.30858200	17.87273200
H	-0.25316700	18.29145300	18.72195300
H	-1.44425100	19.61430000	18.77301500
H	-2.27871300	18.75447200	16.69445800
H	-1.36003100	17.23783200	16.89652000
Mg	1.80217700	15.46577800	18.56075200
B	-3.07811900	17.23875700	19.23755200
H	0.05677300	15.33937700	17.83349400

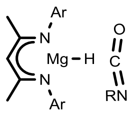

IRC-reverse

O	15.74693900	3.39122300	9.38224600
Mg	12.90777300	-0.45634500	8.60238100
N	12.59685100	-1.46226500	6.86053200
N	11.32211100	-1.25696000	9.58988200
C	10.55849500	-2.51357200	7.65647500
H	9.76453300	-3.17851900	7.33636500
C	11.54304600	-2.25698000	6.68058900
C	10.45762000	-2.07489200	8.99388900
C	11.36149400	-2.95838300	5.35197300
H	12.20072300	-3.63337000	5.14727400
H	10.44286600	-3.54600100	5.33304900
H	11.32629000	-2.23947900	4.52549700
C	9.27471300	-2.60126400	9.77728500
H	8.65674100	-1.78025100	10.15843000

H	8.64477200	-3.24642400	9.16367700
H	9.60680800	-3.17747500	10.64853600
C	14.57367700	3.37300100	9.40141100
N	13.37455600	3.48982800	9.54224800
C	12.20310100	3.05692000	8.93904100
C	12.19417700	2.44034500	7.67559400
C	10.99488100	3.26366100	9.61379900
C	10.98552600	2.03612000	7.10943600
H	13.12991500	2.31004000	7.13965800
C	9.79518200	2.85478200	9.03731700
H	11.01488700	3.74742000	10.58499200
C	9.78235400	2.23756600	7.78708900
H	10.98830200	1.56333900	6.13166800
H	8.86369500	3.02277500	9.57061500
H	8.84521800	1.91917300	7.34105500
C	13.56058200	-1.31478700	5.77921200
H	14.37484100	-0.66269500	6.11151300
H	14.01570400	-2.26906100	5.47936300
H	13.12667200	-0.86135200	4.87584600
C	11.10793800	-0.90194700	10.98586300
H	11.11501400	-1.77486400	11.65362800
H	11.91350900	-0.23802000	11.31461600
H	10.16141500	-0.36768600	11.14820100
H	14.30490800	0.42394900	9.12653400

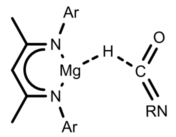

TS

O	15.72015500	2.48773200	9.64047800
Mg	12.67140400	-0.10241600	8.46856600
N	12.54043100	-1.28516900	6.82283400
N	11.24137100	-1.05143700	9.54939600
C	10.62764200	-2.50027300	7.69668600
H	9.91309000	-3.26781500	7.42267900
C	11.58501100	-2.20635400	6.70419500
C	10.47945800	-1.99958200	9.00787700
C	11.49718600	-3.02222900	5.43305300
H	12.41068700	-3.60863300	5.28048600
H	10.65327500	-3.71256200	5.45952100
H	11.38527200	-2.37720000	4.55408500
C	9.37352300	-2.62072000	9.83301100
H	8.68020400	-1.85712600	10.20322000
H	8.80381900	-3.34801300	9.25339300
H	9.78096600	-3.13184500	10.71304700
C	14.55568700	2.57985000	9.47351500
N	13.46217000	3.17760000	9.48911000
C	12.25431000	2.87081400	8.90624100
C	12.15342900	2.35632600	7.59354400
C	11.07013800	3.10989200	9.62470800
C	10.89900300	2.08340700	7.03431900

H	13.05611600	2.25886600	6.99624200
C	9.83130100	2.83465000	9.05660400
H	11.14909400	3.51732000	10.62763300
C	9.73534100	2.31189700	7.76424100
H	10.84201300	1.70021900	6.01947000
H	8.92872800	3.02778400	9.63007700
H	8.76452700	2.09722900	7.32862200
C	13.49613200	-1.11817900	5.73745400
H	14.22698800	-0.35202200	6.01594300
H	14.06249900	-2.03485500	5.52227900
H	13.02583800	-0.79510000	4.79716400
C	11.00961400	-0.66892700	10.93531600
H	11.11351600	-1.51230000	11.63189900
H	11.74742300	0.08386700	11.22879100
H	10.01607200	-0.22696200	11.09388100
H	14.08534600	0.81249500	9.02781200

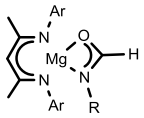

IRC-forward

O	14.39077200	0.06050900	8.27334100
Mg	12.46246800	-0.64312100	8.29649600
N	11.76800900	-1.96094700	6.92463600
N	11.54719700	-1.46237000	9.90586400
C	10.52994400	-3.23035000	8.58624500
H	9.88697900	-4.09739300	8.68375300
C	10.97580500	-2.97333400	7.27305000
C	10.78304500	-2.54690300	9.79421500
C	10.49887000	-3.93854100	6.20980400
H	11.34537600	-4.41979900	5.70669900
H	9.86712700	-4.71954000	6.63456400
H	9.92417200	-3.41838800	5.43480300
C	10.12350900	-3.11769900	11.03070300
H	9.46756500	-2.37840800	11.50461200
H	9.52678500	-3.99922300	10.79357000
H	10.87174700	-3.40266800	11.77933800
C	13.96111800	1.24331600	8.06360700
N	12.66162500	1.45280400	7.99122600
C	12.11352400	2.73161800	7.82710200
C	12.68696500	3.88479700	8.38849100
C	10.91911500	2.86220500	7.10251700
C	12.09830300	5.13191700	8.19451000
H	13.58007100	3.79963800	9.00153100
C	10.33175300	4.10973700	6.92040400
H	10.46692500	1.96947900	6.67889400
C	10.92053500	5.25420400	7.45910200
H	12.55747500	6.01228700	8.63679800
H	9.40830600	4.18854800	6.35259600
H	10.46026300	6.22771200	7.31755100
C	12.15548500	-1.81911500	5.52852300
H	12.78963900	-0.93427300	5.41754200

H	12.73351800	-2.67744900	5.15798200
H	11.29519900	-1.68620800	4.85718600
C	11.72226700	-0.85598500	11.21788500
H	12.19996900	-1.53276300	11.94025800
H	12.36751100	0.02306500	11.12620100
H	10.77560900	-0.51463600	11.65973600
H	14.68527000	2.06063000	7.92346300

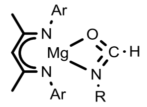

(+HBpin) IRC-reverse

O	7.11788100	3.38654600	4.60650100
N	6.35066700	6.65679900	2.41807400
N	4.80757300	4.10187800	2.61293100
C	5.10509100	8.42143300	1.23580000
H	5.06597600	9.13022900	2.07113400
H	4.19147700	8.53973500	0.65134000
H	5.95477900	8.71997600	0.61142700
C	5.25667800	7.00097800	1.74121100
C	4.18029300	6.13554400	1.45755300
H	3.37246400	6.57917000	0.88639000
C	3.93816900	4.82144800	1.91582400
C	2.57855500	4.24419200	1.57906000
H	2.66698800	3.28257200	1.06071600
H	2.00215800	4.92291900	0.94856400
H	1.99969100	4.05578300	2.49076300
C	8.27673400	3.86475200	4.82438400
H	8.91438100	3.39868400	5.59309000
C	9.94477100	5.47534200	4.36759400
C	10.69979100	5.97367100	3.29308600
C	11.93409700	6.57885300	3.50662000
H	12.50133900	6.95167600	2.65749400
C	12.44611800	6.70519100	4.79809700
H	13.40945600	7.17849700	4.96431900
C	11.69794600	6.23009700	5.87435200
H	12.07409000	6.33960500	6.88841600
C	10.45732200	5.63265600	5.66776500
C	4.39671300	2.78293800	3.07832600
H	5.19262500	2.36128600	3.69441300
H	3.48748400	2.81511700	3.69494300
H	4.20131200	2.08238500	2.25132500
C	7.33202500	7.69726700	2.69709600
H	6.91143500	8.53373400	3.27350900
H	8.14693900	7.27376700	3.28598100
H	7.77602100	8.12321700	1.78461000
H	10.30312800	5.86883000	2.28711200
H	9.86564700	5.30978800	6.51998800
N	8.70290500	4.88272800	4.10760600
C	7.90250800	3.66557200	-1.04389300
C	6.85009100	3.92929700	0.04082400
B	8.80621500	3.19651500	0.97444700

O	8.98927100	3.02446200	-0.35823900
O	7.60428400	3.81732100	1.27681800
H	7.53783600	3.00690800	-1.83600600
H	8.27578000	4.58971700	-1.49889400
H	6.06219900	3.17224800	0.06247200
H	6.39034400	4.91813000	-0.00406200
Mg	6.74278100	4.72433500	3.06060800
H	9.57954800	2.85577400	1.80268100

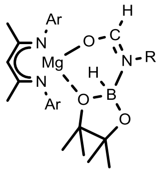

TS

O	7.09193400	3.78992500	4.48874200
N	5.98837700	6.50796000	2.33082000
N	4.38939600	3.94917200	2.55166900
C	4.62679000	8.37836600	1.49638500
H	4.79607600	9.02784100	2.36286900
H	3.62194700	8.57178700	1.11891900
H	5.34621500	8.68559800	0.72853100
C	4.80591500	6.92170800	1.86911300
C	3.67307300	6.10287800	1.69834400
H	2.80590200	6.60696500	1.28733200
C	3.46293800	4.74619000	2.03464300
C	2.06937000	4.20916500	1.78884700
H	2.09434500	3.32638100	1.13998000
H	1.42853200	4.95871700	1.32265500
H	1.59937800	3.89699700	2.72876000
C	8.30720600	4.18352200	4.60753400
H	8.78498600	4.03958200	5.59135900
C	10.23367500	5.28231400	3.90027500
C	11.31532500	5.04903900	3.03478500
C	12.55720000	5.62615200	3.28073100
H	13.38266300	5.42401600	2.60252200
C	12.75243700	6.45285500	4.38803000
H	13.72316300	6.90353000	4.57418700
C	11.68325200	6.70065800	5.24660400
H	11.81460600	7.35221600	6.10700300
C	10.43561900	6.12976600	5.00428000
C	4.02993000	2.58181000	2.89856300
H	4.89107500	2.08937900	3.35944600
H	3.20559200	2.52855700	3.62333100
H	3.73851900	1.98311900	2.02302300
C	7.04529000	7.49540500	2.51665100
H	6.78178500	8.26121900	3.26027100
H	7.94563200	6.98948200	2.87376900
H	7.30946200	8.02407100	1.58867700
H	11.17084800	4.39360300	2.18166200
H	9.59789500	6.35780500	5.65850700
N	8.97662600	4.71683100	3.61905300

C	9.10859800	4.46006400	-0.13796100
C	7.58605000	4.42168700	0.02551100
B	8.61384100	3.04975100	1.55510300
O	9.58569000	3.36831100	0.65772000
O	7.39487000	3.68139300	1.25704000
H	9.42820900	4.31957600	-1.17354500
H	9.54094700	5.38906000	0.24981700
H	7.08694600	3.86849000	-0.77610900
H	7.12716000	5.40679100	0.13108200
Mg	6.31484500	4.55630100	2.84949400
H	8.69769300	2.18272200	2.35507000

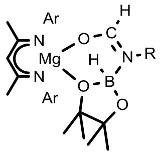

IRC-forward

O	7.17346800	5.25742800	4.22789300
N	5.13094900	7.02473500	2.12266400
N	4.50159300	4.06775700	2.34290600
C	3.14261200	8.36988000	1.59688900
H	3.26949300	9.06166700	2.43751900
H	2.07392200	8.23978200	1.42227200
H	3.57470100	8.85919500	0.71620700
C	3.82284200	7.04639400	1.87371400
C	2.99274600	5.90779500	1.84450200
H	1.95142600	6.11229200	1.62392200
C	3.29277400	4.54486200	2.05740400
C	2.12618600	3.58713200	1.94474700
H	2.30281900	2.84178000	1.16076500
H	1.19948400	4.11300100	1.71185600
H	1.98007800	3.03369000	2.87939000
C	8.42143700	5.18902700	4.39510200
H	8.83628200	5.51555000	5.35912500
C	10.66964400	4.79871800	3.80309100
C	11.49210900	3.68351100	3.60180900
C	12.84509200	3.75475600	3.91727600
H	13.47426900	2.88314700	3.75930600
C	13.39468300	4.92694500	4.43835900
H	14.45162800	4.97354000	4.68448300
C	12.57993100	6.03988600	4.62995300
H	12.99771100	6.96396100	5.02008700
C	11.22624300	5.98227600	4.30316100
C	4.65909000	2.63313600	2.54289300
H	5.70999900	2.40951400	2.74684700
H	4.07482700	2.25752700	3.39512200
H	4.36994900	2.04605900	1.65981000
C	5.86398200	8.28141600	2.12400500
H	5.51781800	8.97870100	2.90070300
H	6.92188000	8.08229300	2.32268200
H	5.81137000	8.81064800	1.16152000
H	11.06618400	2.77427400	3.19489300

H	10.60092500	6.86419400	4.41344700
N	9.28242000	4.72976200	3.50062900
C	9.78049800	4.81027400	0.32982000
C	8.33181600	5.28496500	0.20749700
B	8.70009400	4.00287800	2.18086300
O	9.71221700	3.71573300	1.21524700
O	7.79937500	5.02614500	1.50535000
H	10.20157400	4.49040500	-0.62965700
H	10.41593200	5.61588600	0.73440800
H	7.78154200	4.70102000	-0.54196600
H	8.23800100	6.34977300	-0.03105700
Mg	6.11179800	5.29284800	2.53804200
H	8.06773600	3.03311500	2.55883200

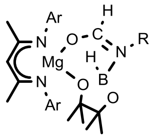

(+HBpin) IRC-reverse

Mg	5.25277800	5.94493600	3.48308000
O	4.86812500	5.66794200	5.48852600
O	7.18646900	6.08233400	4.08688000
O	8.80143500	7.13054900	5.41434000
N	5.60675900	6.70774500	1.56776100
N	4.37251700	4.22584700	2.71868100
N	6.83779900	6.11653500	6.57422400
C	5.51358300	6.78098900	-0.89004000
H	5.11790200	7.80227700	-0.93335500
H	5.08686200	6.21335000	-1.71823700
H	6.59500600	6.85727200	-1.05329600
C	5.21246700	6.12740000	0.44337800
C	4.51500200	4.90005400	0.38565800
H	4.26406300	4.56207700	-0.61352900
C	4.16509600	4.00830700	1.42147400
C	3.51198000	2.71547800	0.97574100
H	4.11156200	1.84945400	1.27882900
H	3.39405100	2.68241200	-0.10824400
H	2.52337700	2.58690300	1.43139900
C	7.56726400	5.95278200	7.78162400
C	7.58975400	4.71722600	8.43908500
C	8.27491800	4.57405300	9.64448000
H	8.28235500	3.60922000	10.14436000
C	8.96305900	5.65344800	10.19247500
C	8.96308200	6.87816800	9.52313600
H	9.50494300	7.72389900	9.93768500
C	8.27044800	7.03339100	8.32718100
C	8.46989200	5.69488500	3.61096600
C	9.38965800	6.77185200	4.18939500
B	7.38136300	6.93623000	5.31383500
H	6.68589700	7.94234800	5.20753300
H	8.72888200	4.69968500	3.99857500
H	8.46183400	5.65832900	2.51659100

H	9.44407300	7.63617700	3.50529800
H	10.41043600	6.40529400	4.35259600
C	3.98196200	3.17987800	3.65589600
H	4.17750200	3.52699600	4.67208200
H	2.91244700	2.92861900	3.59798300
H	4.54279300	2.24513500	3.50706100
C	6.33943400	7.96218700	1.48191200
H	5.73718900	8.78320600	1.06282100
H	6.65248300	8.26586500	2.48449200
H	7.25000700	7.88866100	0.86975000
C	5.60504900	5.63577600	6.50786500
H	5.20339200	5.19384600	7.43149800
C	2.61746100	7.38644800	2.52315200
C	1.26534600	7.67819700	3.18505900
B	2.89896900	7.92380600	4.72640600
O	3.53784900	7.33939300	3.64256500
O	1.59434300	8.20708500	4.47828900
H	3.44595400	8.17381200	5.74452800
H	2.65932600	6.43719100	1.98642800
H	2.94267300	8.18870600	1.85477000
H	0.66441400	6.77213500	3.32184900
H	0.67268000	8.41315500	2.63435900
H	8.27514100	7.98285600	7.80424200
H	9.50470600	5.54027000	11.12724300
H	7.09223000	3.86152200	7.99076300

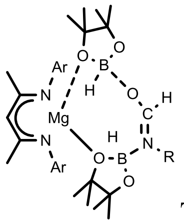

TS

Mg	5.33955200	6.39009800	3.19937600
O	4.82345700	6.51962300	5.24353700
O	7.21973700	6.22494600	3.95225100
O	9.01119300	6.62238000	5.39285300
N	5.68580600	7.01406500	1.26325500
N	4.61035200	4.54678700	2.62463400
N	6.75282200	6.40270300	6.45153100
C	5.36622600	6.97457200	-1.17253300
H	4.94041900	7.98334500	-1.21729500
H	4.88041300	6.36312400	-1.93422200
H	6.42595300	7.06759800	-1.43751800
C	5.20448900	6.37371400	0.20828100
C	4.54435000	5.12247700	0.26409000
H	4.21779400	4.73818000	-0.69559900
C	4.32422500	4.25824000	1.35497700
C	3.72411800	2.90942300	1.01463300
H	4.40653100	2.09665600	1.28942600
H	3.51297200	2.82566900	-0.05223800
H	2.79117300	2.73536300	1.56320600

C	7.33240300	6.12038900	7.72156000
C	6.91207200	5.00945500	8.45988300
C	7.45679700	4.75892000	9.71827500
H	7.12285700	3.89325600	10.28343600
C	8.43510000	5.60244200	10.23786800
C	8.86918000	6.69737900	9.48907000
H	9.63786300	7.35562200	9.88401400
C	8.32557800	6.96042000	8.23649700
C	8.27070000	5.31953200	3.61238800
C	9.49792100	5.97743900	4.24078200
B	7.63264200	6.95837600	5.18908500
H	7.34492400	8.13958900	5.09096300
H	8.07460600	4.33354900	4.05581800
H	8.32716400	5.21630900	2.52468800
H	9.94527000	6.70302500	3.54157700
H	10.26895500	5.24646300	4.51237400
C	4.39883300	3.51522600	3.62891900
H	4.66117000	3.91373100	4.61233600
H	3.35433500	3.17428800	3.69021300
H	5.01942000	2.62234100	3.46212000
C	6.38147400	8.27611000	1.06126200
H	5.72029100	9.07039500	0.68472900
H	6.78993600	8.62107600	2.01592000
H	7.22635800	8.19345900	0.36302900
C	5.44771700	6.37044700	6.34978600
H	4.85727400	6.22349600	7.26114400
C	2.39170800	7.20061900	2.89228300
C	1.69128100	6.47592700	4.04253800
B	3.37619800	7.72692900	4.91337600
O	3.61287700	7.67519100	3.49118100
O	2.15981600	7.14007000	5.21409000
H	3.78602300	8.62941900	5.57765700
H	2.62748000	6.55691000	2.04088700
H	1.81747300	8.06766800	2.54453800
H	1.97219900	5.41517900	4.07242700
H	0.60061200	6.54975700	3.99298300
H	8.66700300	7.80611800	7.65116600
H	8.86530200	5.40351500	11.21516900
H	6.17938200	4.32752900	8.03675200

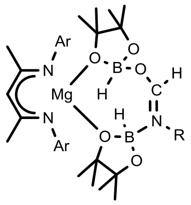

IRC-forward

Mg	5.12883500	6.09075100	3.03698800
O	5.03912400	7.36795200	4.91895600
O	6.70416100	5.39713200	4.14487800
O	8.75871900	5.63304700	5.24348700
N	5.63789400	7.27007800	1.44692400

N	4.67841800	4.40722200	1.93440300
N	6.79374400	6.81466300	6.22946100
C	5.86422200	7.76938900	-0.94522100
H	5.23907000	8.66926800	-0.91924500
H	5.72359800	7.28471600	-1.91219100
H	6.90514600	8.10611300	-0.87975700
C	5.52145700	6.83734400	0.19800600
C	5.09325200	5.54199900	-0.17667000
H	5.05817100	5.37417400	-1.24694400
C	4.71450500	4.43179800	0.60162700
C	4.32956100	3.18787600	-0.17309700
H	4.98384400	2.34514900	0.07936400
H	4.39303800	3.35365100	-1.24916700
H	3.30585000	2.87520800	0.06341600
C	7.33916100	6.72152000	7.54368500
C	6.57496600	6.21100300	8.59727500
C	7.10932100	6.15947900	9.88334100
H	6.50855600	5.76258600	10.69671400
C	8.40994000	6.59767000	10.11953700
C	9.17728900	7.08380500	9.06034100
H	10.19584800	7.41838500	9.23509700
C	8.65038100	7.14716900	7.77460000
C	7.37483600	4.13553400	4.12989400
C	8.83151300	4.50966100	4.39978000
B	7.58923300	6.37532200	4.88352100
H	7.74080300	7.38934300	4.22822700
H	6.97503700	3.49408300	4.92746400
H	7.21568500	3.64949100	3.16287300
H	9.34463300	4.75463300	3.45521000
H	9.38678700	3.70326700	4.89273100
C	4.27809600	3.17287200	2.58868200
H	4.32521300	3.30417600	3.67551000
H	3.24878300	2.86607100	2.34671000
H	4.92947800	2.31924800	2.34785800
C	6.09145300	8.63509600	1.67372400
H	5.41028500	9.38413200	1.24508400
H	6.14629700	8.82279400	2.74868200
H	7.09349200	8.82398900	1.26342100
C	5.59064200	7.28899300	6.08090200
H	5.01132900	7.62769800	6.94165100
C	2.11748800	5.88288600	4.11137500
C	2.23603400	5.93928500	5.63391900
B	3.45888900	7.67836200	4.75532900
O	3.19920700	6.70230100	3.66584500
O	2.72583800	7.24316300	5.89465400
H	3.38206600	8.84794100	4.48319500
H	2.21367700	4.87832900	3.69116500
H	1.17114700	6.32094100	3.76960500
H	2.93620700	5.16886500	5.99956600
H	1.27708600	5.79368400	6.14295400

H	9.24603400	7.51392100	6.94683200
H	8.82832800	6.55121300	11.12076200
H	5.57382700	5.83375500	8.40742100

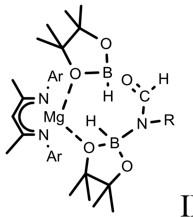

IRC-reverse

O	4.92914900	4.74734100	6.02228600
O	7.53845700	4.00380400	5.01489000
O	9.11619000	5.51855500	5.74099700
N	5.66524800	7.28658400	1.12762800
N	5.13137200	4.44053800	1.98788700
N	6.91839000	5.61288200	6.83472000
C	5.03413000	7.70955100	-1.21007700
H	4.48278100	8.62900000	-0.98250800
H	4.53248700	7.20488700	-2.03710800
H	6.02959600	8.01529900	-1.55303200
C	5.13373800	6.81448700	0.00756400
C	4.65324700	5.49605500	-0.15303500
H	4.24241200	5.27817600	-1.13246000
C	4.68846100	4.39902000	0.73181400
C	4.18491500	3.08653500	0.16566900
H	4.97722700	2.32897600	0.16634300
H	3.83064600	3.20505300	-0.85918700
H	3.36367600	2.68072600	0.76755100
C	7.43550000	6.47725500	7.85709400
C	7.30099100	6.12057400	9.20043900
C	7.76333300	6.97813600	10.19775200
H	7.65372900	6.69686400	11.24139700
C	8.37556600	8.18185900	9.85581400
C	8.51974500	8.52817700	8.51183700
H	8.99634700	9.46590000	8.23985500
C	8.04574400	7.68506200	7.51074400
C	8.66240900	3.84409100	4.14703800
C	9.78050800	4.69722300	4.78159000
B	7.81571900	5.09215000	5.80174600
H	8.91682900	2.78206900	4.08008700
H	8.39188200	4.20943200	3.14938900
H	10.28774600	5.33223500	4.04906200
H	10.53179400	4.08762500	5.29823800
C	5.15199300	3.19998200	2.75475700
H	5.52291000	3.40123900	3.76231400
H	4.15602000	2.74080800	2.85195100
H	5.80938100	2.43868600	2.30873800
C	6.17882800	8.64688800	1.14470400
H	5.39248300	9.40379100	1.00191100
H	6.64484500	8.83711200	2.11674300
H	6.94701800	8.82629800	0.37859400

C	5.56314300	5.38706400	6.84502600
H	5.07125500	5.87992200	7.70496100
C	2.78057900	5.82220400	4.02138300
C	2.07349600	6.28957700	5.30300400
B	3.69511700	7.77705700	4.78571800
O	3.88149700	6.75558700	3.87011000
O	2.62283600	7.58692400	5.58957300
H	4.40464600	8.72531900	4.83491500
H	3.18924400	4.81698400	4.11132000
H	2.15661900	5.89667600	3.12669700
H	2.29439400	5.62754500	6.14429800
H	0.99079500	6.38156600	5.18286900
H	8.14219600	7.94574700	6.46205400
H	8.74199100	8.84696600	10.63267300
H	6.84795900	5.16702500	9.45802000
Mg	5.74473300	6.19961600	2.87769100
H	7.02471700	6.62852100	4.01663100

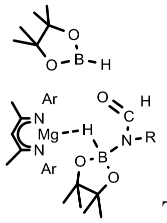

TS

O	4.93159100	4.73890300	6.02647000
O	7.54098100	4.00163100	5.01693700
O	9.11794800	5.51617300	5.74500800
N	5.66034600	7.29075900	1.12566200
N	5.13424300	4.44240800	1.98322700
N	6.91936300	5.61001600	6.83679700
C	5.02282700	7.71562100	-1.20995900
H	4.47022400	8.63366600	-0.97977300
H	4.52015100	7.21110000	-2.03645000
H	6.01683900	8.02380500	-1.55494600
C	5.12718400	6.81907700	0.00619500
C	4.64911400	5.49985800	-0.15513500
H	4.23654700	5.28247000	-1.13394100
C	4.68860300	4.40165600	0.72810500
C	4.18655700	3.08889700	0.16126900
H	4.98053500	2.33308300	0.15896200
H	3.82959500	3.20811000	-0.86257200
H	3.36764200	2.68042200	0.76450800
C	7.43487300	6.47718900	7.85766300
C	7.30127400	6.12249000	9.20161400
C	7.76202200	6.98267600	10.19740700
H	7.65314000	6.70296200	11.24154500
C	8.37173100	8.18707100	9.85332500
C	8.51497600	8.53143800	8.50874700
H	8.98959700	9.46968400	8.23511500
C	8.04255500	7.68565600	7.50915300
C	8.66539800	3.84291000	4.14940800

C	9.78290600	4.69612100	4.78489900
B	7.81786700	5.08875900	5.80536300
H	8.92028400	2.78103300	4.08195000
H	8.39499900	4.20881700	3.15196300
H	10.28978500	5.33218700	4.05305400
H	10.53451000	4.08652400	5.30107700
C	5.15915700	3.20094600	2.74847200
H	5.53181500	3.40164900	3.75553600
H	4.16440000	2.73951200	2.84729200
H	5.81720200	2.44161000	2.30007500
C	6.17109000	8.65212400	1.14344000
H	5.38271500	9.40757700	1.00419400
H	6.63954600	8.84178300	2.11441900
H	6.93664200	8.83442100	0.37538500
C	5.56434500	5.38200500	6.84741900
H	5.07165300	5.87628900	7.70609100
C	2.78550700	5.81634600	4.02433500
C	2.07806600	6.28288900	5.30611100
B	3.69735000	7.77275900	4.78813400
O	3.88465600	6.75171900	3.87226600
O	2.62596400	7.58084900	5.59274000
H	4.40540900	8.72213300	4.83689300
H	3.19612900	4.81195000	4.11465100
H	2.16105700	5.88931500	3.12987700
H	2.29979300	5.62100900	6.14729600
H	0.99524900	6.37371100	5.18611400
H	8.13828800	7.94475700	6.45999400
H	8.73690600	8.85424200	10.62900200
H	6.85016300	5.16847900	9.46085800
Mg	5.74640900	6.20145100	2.87387000
H	7.02789300	6.62888200	4.01145700

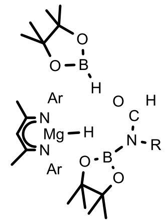

IRC-forward

O	4.93022400	4.73511400	6.03144600
O	7.53793100	3.99608800	5.02148500
O	9.11811800	5.50673200	5.75051800
N	5.66145400	7.29094300	1.12158500
N	5.13220300	4.44374800	1.98131500
N	6.91854800	5.60744500	6.83938600
C	5.02123300	7.71551100	-1.21334800
H	4.47080300	8.63475900	-0.98278000
H	4.51622500	7.21147800	-2.03871200
H	6.01527200	8.02155600	-1.56016800
C	5.12591800	6.81946200	0.00314300
C	4.64551200	5.50093200	-0.15679300
H	4.23113600	5.28371600	-1.13487400

C	4.68456200	4.40308100	0.72692300
C	4.17954200	3.09085300	0.16150800
H	4.97217400	2.33363200	0.15879500
H	3.82159900	3.21003100	-0.86199400
H	3.36059300	2.68422700	0.76595600
C	7.43435500	6.47717300	7.85796500
C	7.29994500	6.12644000	9.20287200
C	7.76092900	6.98915600	10.19636000
H	7.65141700	6.71254700	11.24125900
C	8.37163700	8.19211800	9.84904300
C	8.51565600	8.53251800	8.50353800
H	8.99103900	9.46963900	8.22739000
C	8.04302500	7.68419000	7.50619900
C	8.66275100	3.83412200	4.15501200
C	9.78181400	4.68493000	4.79097300
B	7.81717700	5.08208200	5.81039100
H	8.91509400	2.77158500	4.08839900
H	8.39401600	4.20017100	3.15720200
H	10.29052300	5.31974800	4.05933400
H	10.53173000	4.07377400	5.30775200
C	5.15629900	3.20269400	2.74722200
H	5.53033100	3.40334900	3.75381700
H	4.16105400	2.74262500	2.84742700
H	5.81283200	2.44222000	2.29852500
C	6.17448300	8.65147500	1.13780800
H	5.38715400	9.40816500	0.99938900
H	6.64476400	8.84091900	2.10794900
H	6.93913800	8.83206900	0.36846300
C	5.56316600	5.38075600	6.85013300
H	5.07067000	5.87868700	7.70682500
C	2.78967900	5.82002500	4.02657500
C	2.08262500	6.28994700	5.30734800
B	3.70426400	7.77651800	4.78708300
O	3.89008700	6.75370600	3.87291100
O	2.63267200	7.58741500	5.59208200
H	4.41366100	8.72498700	4.83427500
H	3.19901200	4.81530200	4.11901800
H	2.16546600	5.89207300	3.13188500
H	2.30283800	5.62901000	6.14966600
H	1.00001100	6.38248400	5.18681700
H	8.13928500	7.94022000	6.45632600
H	8.73697400	8.86126400	10.62294100
H	6.84800600	5.17352400	9.46468600
Mg	5.74913300	6.20214100	2.86992500
H	7.03270300	6.62621800	4.00618000

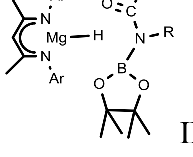

IRC-reverse

Mg	10.46889100	-1.76241100	10.24751900
N	9.60566000	-1.05098900	8.52092900
N	9.09098100	-0.86373200	11.48339500
C	8.04876200	0.39698800	9.69106800
H	7.29941800	1.16002300	9.51236300
C	8.65513900	-0.12295900	8.52765100
C	8.21985500	0.03864200	11.04486300
C	8.14669600	0.43288100	7.21331000
H	7.68812700	-0.35569000	6.60501800
H	7.40092900	1.21316000	7.37224600
H	8.96472700	0.85553600	6.61853300
C	7.31568000	0.74291600	12.03526800
H	7.89823700	1.23593800	12.82183200
H	6.69604200	1.49523400	11.54495000
H	6.65020200	0.02893100	12.53486800
H	11.20310500	-3.35679300	10.48626000
C	9.12534000	-1.17488400	12.90259800
H	9.45248100	-0.32461300	13.52106400
H	8.15429300	-1.50950600	13.29549200
H	9.83477400	-1.99189000	13.07131900
C	10.10908900	-1.53109500	7.24472800
H	9.32225800	-1.94764700	6.59910900
H	10.62788400	-0.75101200	6.66663100
H	10.83029000	-2.33521100	7.42467500
O	15.95518800	1.34668800	11.15537700
O	13.78692000	1.69761800	10.47175500
O	12.28250100	-0.64978500	10.49741500
N	14.54785200	-0.64765700	10.88939800
C	15.85195200	2.75475000	10.91848800
C	14.35108300	3.00136800	10.64642000
C	13.33555800	-1.25199700	10.69940400
H	13.35413700	-2.35238100	10.73887500
C	15.65847900	-1.51058800	11.19563700
C	15.58562200	-2.36851100	12.29405800
C	16.64516800	-3.23061700	12.57071000
H	16.58118400	-3.90090900	13.42278100
C	17.78192700	-3.22431600	11.76571800
C	17.85515600	-2.35579900	10.67668100
H	18.73799300	-2.34767900	10.04403600
C	16.79428700	-1.50351200	10.38483000
B	14.74264300	0.79994900	10.83517600
H	18.60906400	-3.89283600	11.98569400
H	16.84115000	-0.83473600	9.53151100
H	14.70687500	-2.35381000	12.93264200
H	16.48140300	3.01488600	10.05989100
H	16.22121600	3.29458600	11.79513000
H	13.84859800	3.49083700	11.48797700
H	14.17236900	3.59292900	9.74411900

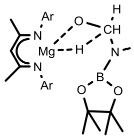

TS

Mg	11.16623300	-1.00027100	10.69779500
N	10.43262900	-0.48563700	8.81827500
N	9.39047300	-0.46439500	11.61858400
C	8.47256600	0.65100100	9.66127600
H	7.63933300	1.27008900	9.34778600
C	9.33642800	0.23908300	8.62684200
C	8.46281500	0.27969800	11.02304800
C	8.93687000	0.64650300	7.22321200
H	8.67834500	-0.23354000	6.62241100
H	8.07228600	1.31224000	7.23260900
H	9.75724200	1.15259700	6.70124200
C	7.27320300	0.76995000	11.82373000
H	7.59323300	1.33702100	12.70518000
H	6.62498300	1.40846700	11.22178200
H	6.67416400	-0.07125600	12.19150600
H	12.35097000	-2.36894300	10.34065400
C	9.17505600	-0.83858300	13.01072300
H	9.11628000	0.03106400	13.68179600
H	8.25450500	-1.42351500	13.15190400
H	10.01511800	-1.44626600	13.34992100
C	11.17294000	-0.95346600	7.65749400
H	10.55332600	-1.53413200	6.95902300
H	11.63101600	-0.13721100	7.07420200
H	11.97636000	-1.61161400	8.00225700
O	14.98680900	1.09118300	10.41823500
O	12.74527000	0.63570800	10.64452000
O	12.25369900	-1.13992800	12.58900500
N	14.32913200	-1.00254800	11.58407400
C	14.29529900	2.22344800	9.87747800
C	12.81781900	1.79516300	9.79448300
C	13.25823100	-1.71149500	12.13073700
H	13.49736500	-2.76046600	12.34207100
C	15.60242200	-1.64459700	11.52652500
C	16.14363100	-2.19692000	12.69139200
C	17.36986400	-2.85613000	12.64573800
H	17.78224100	-3.28399000	13.55502200
C	18.07128500	-2.94975000	11.44532600
C	17.53613200	-2.38642300	10.28723200
H	18.07541100	-2.45847400	9.34699700
C	16.30219200	-1.74342000	10.32090500
B	14.04556600	0.23711700	10.91185700
H	19.03119600	-3.45676000	11.41198200
H	15.87644100	-1.32151300	9.41675000
H	15.61089900	-2.09093900	13.63238900
H	14.71841700	2.47095400	8.90018500
H	14.44261800	3.07666700	10.54891100

H	12.12451100	2.55114000	10.16894400
H	12.51249300	1.50130000	8.78684900

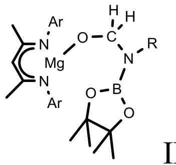

IRC-forward

Mg	11.14585400	-0.95733900	10.95245300
N	10.55995400	0.99094600	10.79264500
N	9.41681500	-1.74039500	10.20753500
C	8.51072700	0.43365300	9.61608300
H	7.65850300	0.89433500	9.12962800
C	9.43846000	1.34241000	10.16953100
C	8.47174900	-0.97660100	9.66617600
C	9.08926200	2.80946500	10.03778100
H	9.89128100	3.36747500	9.54130700
H	8.16961300	2.95162900	9.46872600
H	8.95213900	3.26661900	11.02458400
C	7.24392600	-1.62737500	9.06556500
H	6.67516200	-2.16785900	9.83122500
H	6.58188400	-0.88846400	8.61230100
H	7.51721700	-2.36126300	8.29891900
H	13.22541700	-3.20334700	11.23630400
C	9.22113200	-3.18255200	10.24428100
H	8.30705300	-3.47637500	10.77898200
H	9.17482300	-3.63483000	9.24246900
H	10.06123300	-3.64552100	10.77086700
C	11.40035900	2.02889600	11.37316900
H	11.79847700	2.72861200	10.62329300
H	10.87584800	2.62742600	12.13135000
H	12.25552400	1.56116900	11.86970300
O	14.68612400	-0.32527000	8.88547100
O	12.63335100	-1.22432900	9.42738500
O	12.32493100	-1.65305300	12.25830500
N	14.41608300	-1.49820600	11.08203400
C	13.76435100	0.12742300	7.89617100
C	12.53428700	-0.77281200	8.06409000
C	13.44371100	-2.32496000	11.87742800
H	14.02449500	-2.70151300	12.73422700
C	15.60036800	-1.04408800	11.71238100
C	15.57571600	-0.67554400	13.06599800
C	16.73864700	-0.24230800	13.69630000
H	16.70205600	0.03864100	14.74573700
C	17.93749500	-0.15121800	12.98959000
C	17.96078100	-0.50390400	11.64138000
H	18.88841400	-0.44183200	11.07818400
C	16.80665900	-0.95660800	11.00628200
B	13.98119400	-1.02988100	9.83423800
H	18.84211700	0.19161300	13.48371200
H	16.83327400	-1.24588300	9.96093200

H	14.63497500	-0.71258200	13.60761700
H	14.21757400	0.04164100	6.90455700
H	13.52268000	1.18138600	8.08171000
H	11.58497100	-0.25059700	7.92552000
H	12.56614300	-1.64857000	7.40653100

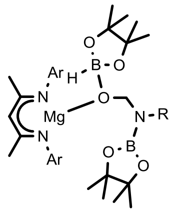

IRC-reverse

N	10.82921700	1.34533300	10.49361500
N	9.24742100	-1.23870600	10.63660200
C	9.00399200	0.60188000	9.07014900
H	8.39189500	0.92984700	8.23767500
C	9.96191400	1.54485900	9.50421400
C	8.62394400	-0.64206500	9.62785100
C	9.95818700	2.87299900	8.77702600
H	10.94461200	3.10361100	8.35878500
H	9.22998900	2.88202600	7.96498100
H	9.71000600	3.68995600	9.46442900
C	7.40458100	-1.29648100	9.01397900
H	6.58654500	-1.34729800	9.74194500
H	7.04824000	-0.74169200	8.14493400
H	7.61561000	-2.32644700	8.70572300
H	13.07839100	-2.63363900	12.49364000
C	8.70429900	-2.48156500	11.17241900
H	7.65744000	-2.38147600	11.49019400
H	8.74938000	-3.30787500	10.44712700
H	9.28501200	-2.77597300	12.05121900
C	11.70324900	2.43900500	10.89570700
H	12.36309700	2.78307900	10.08581700
H	11.14608000	3.31406400	11.25875800
H	12.34485200	2.10361500	11.71528900
O	14.39726800	-0.92961500	9.13222500
O	12.39464200	-1.47229800	10.13797100
O	12.36639100	-0.73470500	12.83479600
N	14.32483900	-1.19954700	11.61542000
C	13.39321900	-0.84280600	8.11721500
C	12.16537500	-1.54260100	8.71331400
C	13.45739400	-1.62710900	12.69922800
H	14.03680200	-1.65625500	13.62589700
C	15.54634000	-0.54240900	11.93466500
C	15.62541300	0.29557200	13.05508100
C	16.82661500	0.92533200	13.37125200
H	16.87356100	1.57119600	14.24388500
C	17.95378700	0.74582300	12.57115800
C	17.86995100	-0.07798600	11.44989800
H	18.74203200	-0.23116700	10.81983600
C	16.67936000	-0.72806800	11.13496400

B	13.76637500	-1.20213100	10.31664800
H	18.88731400	1.24277800	12.81865500
H	16.62170900	-1.38138400	10.27110600
H	14.74317400	0.46644600	13.66533100
H	13.75473600	-1.32624800	7.20568000
H	13.19352800	0.21324400	7.90224200
H	11.22142600	-1.04567100	8.47800300
H	12.10670300	-2.59877800	8.43002000
Mg	10.94759700	-0.45376000	11.44683000
O	10.97691500	-2.43619100	13.87411200
O	11.67840700	-0.67425100	15.23946700
C	10.79561300	-2.83391400	15.21741000
C	11.67096300	-1.85654100	16.01312800
B	11.30138900	-1.01808400	13.90241700
H	11.09194500	-3.88238200	15.34721000
H	9.73746800	-2.73920300	15.51230700
H	11.27619400	-1.65396600	17.01679400
H	12.69352400	-2.25477300	16.12948500
H	10.36717100	-0.28612900	13.45365800

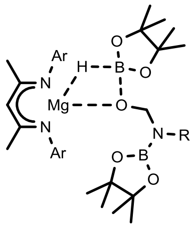

TS

N	11.18788800	1.21848900	10.77182600
N	9.14996000	-0.97119100	11.12419800
C	9.57622300	0.38848900	9.15751100
H	9.21576700	0.56157300	8.14941200
C	10.60702400	1.25773400	9.57740500
C	8.88250500	-0.61322200	9.87395400
C	11.04542900	2.30250300	8.57277900
H	12.12438500	2.24268200	8.39218200
H	10.52384500	2.18598200	7.62127900
H	10.84345200	3.31295600	8.94665800
C	7.75342300	-1.29394400	9.12819400
H	6.79687900	-1.14099200	9.64102600
H	7.65780600	-0.91012200	8.11139300
H	7.91121400	-2.37714900	9.07314200
H	13.25242800	-3.09371700	11.50050900
C	8.32822700	-1.99972500	11.74810000
H	7.27485500	-1.69772000	11.84391800
H	8.34896000	-2.95163000	11.19831800
H	8.70735200	-2.20169600	12.75301600
C	12.19797400	2.21934000	11.09084600
H	13.09963000	2.12068300	10.47179700
H	11.82425700	3.24732400	10.98284700
H	12.50763000	2.09654100	12.13242800
O	13.88544200	-0.31139400	8.85312500
O	12.75375200	-2.25326100	9.36286400

O	12.11831200	-1.81230900	12.61012800
N	13.95524300	-1.17632600	11.19837000
C	13.37890200	-0.82050900	7.61503800
C	12.37660400	-1.91272100	8.02541800
C	13.41614100	-2.17673700	12.06765400
H	14.09704500	-2.35670600	12.90234400
C	14.84830100	-0.20890700	11.74094000
C	14.75201000	0.18328000	13.08317100
C	15.65459700	1.10513300	13.61121400
H	15.56032400	1.39412600	14.65449300
C	16.65364600	1.66110400	12.81695700
C	16.74698700	1.27455800	11.48068300
H	17.52546500	1.68975900	10.84598900
C	15.86239600	0.34334200	10.94547600
B	13.54467200	-1.22514700	9.82469300
H	17.35069800	2.38326800	13.23201600
H	15.95219200	0.04428800	9.90801900
H	13.97389700	-0.21689500	13.72393800
H	14.21479300	-1.22225500	7.02841900
H	12.91416100	-0.00803800	7.04992600
H	11.34846800	-1.53386100	8.03799600
H	12.43081300	-2.80359000	7.39280100
Mg	10.70226400	-0.16471900	12.20136600
O	10.65359300	-2.56741000	14.37130400
O	12.78332000	-1.89488900	14.95297700
C	10.66118200	-2.45743200	15.79508800
C	12.15478000	-2.34118200	16.15937300
B	11.83794000	-2.00005900	13.96074100
H	10.17760200	-3.33567500	16.23158100
H	10.10725900	-1.55598700	16.08258400
H	12.34001200	-1.61575200	16.95643700
H	12.59077600	-3.30491800	16.44978400
H	10.86464500	0.10961900	13.94943400

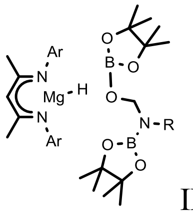

IRC-forward

N	11.20713800	1.23644100	10.74315900
N	9.14761300	-0.90747600	11.19695800
C	9.61089400	0.33169100	9.15815300
H	9.26088900	0.45212900	8.13865500
C	10.63664500	1.22267000	9.54466600
C	8.90011500	-0.61943700	9.92387400
C	11.07461900	2.22764200	8.49988200
H	12.15770500	2.18291300	8.34221000
H	10.57282600	2.05696500	7.54567300
H	10.84447600	3.24969300	8.82264500
C	7.77090700	-1.32852700	9.20453100

H	6.81027100	-1.13831000	9.69668200
H	7.69057600	-1.00020300	8.16715300
H	7.91823100	-2.41462700	9.21037100
H	13.25314200	-3.08089200	11.45505100
C	8.28811100	-1.87362600	11.86818200
H	7.24285900	-1.53634000	11.93003200
H	8.28693400	-2.85749300	11.37704500
H	8.64755700	-2.02668900	12.88826900
C	12.20225100	2.26081100	11.03221100
H	13.11366100	2.14440700	10.43053200
H	11.81986300	3.27898500	10.87294000
H	12.49525300	2.18620900	12.08311000
O	13.88748500	-0.28391000	8.83921500
O	12.78455800	-2.24805200	9.32591000
O	12.12250400	-1.83528200	12.61035600
N	13.93507600	-1.15313900	11.18319400
C	13.41186000	-0.80092800	7.59183600
C	12.42009600	-1.90881900	7.98452700
C	13.41959900	-2.17442800	12.03774500
H	14.11333100	-2.36488600	12.85943000
C	14.81051800	-0.17856400	11.74209300
C	14.68498100	0.20899300	13.08286800
C	15.56987900	1.13572500	13.63132400
H	15.45198100	1.42313900	14.67253600
C	16.58008100	1.70040300	12.85745900
C	16.70189100	1.31922500	11.52186100
H	17.48902100	1.74270800	10.90357600
C	15.83451300	0.38325100	10.96695700
B	13.54585800	-1.20494700	9.80296100
H	17.26339700	2.42679000	13.28767700
H	15.94507900	0.08802900	9.93031400
H	13.89164100	-0.19438000	13.70240100
H	14.26479300	-1.18938500	7.02099700
H	12.94479100	0.00415600	7.01829600
H	11.38660400	-1.54463200	7.98619300
H	12.49556400	-2.79665800	7.34987400
Mg	10.71485700	-0.07351100	12.24594000
O	10.61186600	-2.45950500	14.36410000
O	12.83717300	-2.19148100	14.90680800
C	10.67193700	-2.41864200	15.79310000
C	12.16968200	-2.58645300	16.11230500
B	11.87020500	-2.12546400	13.93588700
H	10.05078200	-3.21727200	16.20715600
H	10.29157600	-1.44746800	16.12904300
H	12.50306700	-1.94971800	16.93598500
H	12.43473700	-3.62631400	16.33864200
H	10.92580300	0.37201300	13.94351500

References

1 A. P. Dove, V. C. Gibson, P. Hormnirun, E. L. Marshall, J. A. Segal, A. J. P. White, D. J. Williams, *Dalton. Trans.* 2003, 3088.

2 S. J. Bonyhady, C. Jones, S. Nembenna, A. Stasch, A. J. Edwards, G. J. McIntyre, *Chem.-Eur. J.*, 2010, **16**, 938.

3 DENZO-SCALEPACK Z. Otwinowski and W. Minor, "Processing of X-ray Diffraction Data Collected in Oscillation Mode", Methods in Enzymology, Volume 276: Macromolecular Crystallography, part A, p.307-326, **1997**,C.W. Carter, Jr. & R. M. Sweet, Eds., Academic Press.

4 L. J. Bourhis, O. V. Dolomanov, R. J. Gildea, J. A. K. Howard, H. Puschmann, *Acta Cryst. A* 2015, **71**, 59-75.

5 C. Barnes, *J. Appl. Cryst*. 1997, **30**, 568.

6 G. M. Sheldrick, *Acta Cryst. A*, 2008, **64**, 112-122.

7 (1) Gaussian 09, Revision D.02, M. J. Frisch, G. W. Trucks, H. B. Schlegel, G. E. Scuseria, M. A. Robb, J. R. Cheeseman, G. Scalmani, V. Barone, B. Mennucci, G. A. Petersson, H. Nakatsuji, M. Caricato, X. Li, H. P. Hratchian, A. F. Izmaylov, J. Bloino, G. Zheng, J. L. Sonnenberg, M. Hada, M. Ehara, K. Toyota, R. Fukuda, J. Hasegawa, M. Ishida, T. Nakajima, Y. Honda, O. Kitao, H. Nakai, T. Vreven, J. A. Montgomery, Jr., J. E. Peralta, F. Ogliaro, M. Bearpark, J. J. Heyd, E. Brothers, K. N. Kudin, V. N. Staroverov, R. Kobayashi, J. Normand, K. Raghavachari, A. Rendell, J. C. Burant, S. S. Iyengar, J. Tomasi, M. Cossi, N. Rega, J. M. Millam, M. Klene, J. E. Knox, J. B. Cross, V. Bakken, C. Adamo, J. Jaramillo, R. Gomperts, R. E. Stratmann, O. Yazyev, A. J. Austin, R. Cammi, C. Pomelli, J. W. Ochterski, R. L. Martin, K. Morokuma, V. G. Zakrzewski, G. A. Voth, P. Salvador, J. J. Dannenberg, S. Dapprich, A. D. Daniels, O. Farkas, J. B. Foresman, J. V. Ortiz, J. Cioslowski, D. J. Fox, Gaussian, Inc., Wallingford CT, 2009.

8 A. D. Becke, *J. Chem. Phys*. 1993, **98**, 5648.

9 J. P. Perdew, Y. Wang, *Phys. Rev. B* 1992, **45**, 13244.

10 (a) A. D. McLean, G. S. Chandler, *J. Chem. Phys.* 1980, **72**, 5639. (b) W. J. Hehre, R. Ditchfield, J. A. Pople, *J. Chem. Phys.* 1972, **56**, 2257.

11 (a) C. Gonzalez, H. B. Schlegel, *J. Chem. Phys*. 1989, **90**, 2154. (b) C. Gonzalez, H. B. Schlegel, *J. Phys. Chem.* 1990, **94**, 5523.

12. S. Grimme, S. Ehrlich, L. Goerigk, *J. Comp. Chem.* 2011, **32**, 1456.
